# Modulation of the cytoskeleton for cancer therapy

**DOI:** 10.3389/fcell.2025.1681065

**Published:** 2026-08-24

**Authors:** Alexandre Matov

**Affiliations:** DataSet Analysis LLC, San Francisco, CA, United States

**Keywords:** microtubule dynamics, solid tumors, drug resistance, resistance disease, sensitizing tumors

## Abstract

**Introduction:**

All degenerative diseases are associated with impairment in intracellular trafficking. The highly dynamic organization and remodeling of the cellular cytoskeleton are dysfunctional in pathology and often lead to drug resistance. Successful analyses of the mechanisms of drug action require statistical analysis of large-scale readouts of molecular interactions at nanometer-scale resolution. The focus of our work is the automated extraction of unbiased information from time-lapse microscopy image series of the response of cytoskeletal meshworks, intracytoplasmic membranous networks, and vesicle trafficking to *ex vivo* drug treatment in cancer. We present the computer vision algorithms we have developed in this regard.

**Methods:**

We have performed cell biological profiling of cellular interactions and molecular mechanisms of pathogenesis and drug resistance. We are developing a platform for multifaceted analyses of intracellular and intercellular dynamics in patient-derived cultures. We aim to correlate our analyses with patterns of genetic and epigenetic variations in order to anticipate drug resistance and unfavorable treatment outcome. While we have established organoid cultures from solid tumor samples, we will perform preclinical and clinical analyses in a clinically-relevant model system in kidney organoids obtained from patient urine samples for which we will perform sequencing of long and small RNAs and will measure the expression levels of microtubule (MT) regulators and other cytoskeletal modulators. We label MT ends and will label other cellular components, such as actin, E-cadherin, cytoplasmic dynein, mitochondria, lysosomes, or exosomes, depending on the tumor type and disease stage. Our technology allows us to build a medical digital twin.

**Results:**

We report new function of the MT-stabilizing drug paclitaxel and the MT-destabilizing drug vinorelbine, which elucidate mechanisms beyond the canonical function of these tubulin inhibitors. We present new analysis results on patient stratification using non-invasive biomarkers, such as urinary small RNA. We identified dysregulated MT-regulating genes in colorectal cancer organoids that can be linked to spindle rotation during mitosis and resistance to MT-stabilizing drugs. The paper provides new results from our work with patient-derived cells cultured as organoids and the analysis of cancer vulnerabilities in the context of precision medicine. We report real-time computer vision analysis and lattice light-sheet live-cell organoid imaging before drug treatment and will extend this work after treatment with low drug doses of MT, GSK3β, or/and tropomyosin inhibitors, and small molecules that induce ferroptosis or inhibit Rho GTPase activity.

**Conclusion:**

Our biomedical computer vision approach allows us to elucidate mechanisms of drug action and uncover molecular interactions inaccessible by sequencing methods alone. After clinical validation, it may contribute to anticipating drug resistance and identifying sensitizing regimens that lead to complete response with minimal toxicity and the elimination of residual disease.

## Introduction

### Mitotic spindle

The cell cycle is an ordered set of events, culminating in cell growth and division. A particularly critical moment during cell division is the replication and symmetric distribution (Schaede, 1929) of the cell’s genome into the two daughter cells (Inoué, 1959). To achieve this, cells build specialized machinery, the mitotic spindle, consisting of a scaffold of MTs and molecular motor proteins (Vale et al., 1985). In normal physiology, the mitotic spindle is a bipolar structure to which the sister chromatids containing the symmetrically replicated genes bind (Heath, 1980). The mitotic spindle of higher metazoans consists of MTs emanating from the two spindle poles and MT-associated proteins (Rieder et al., 1986). MTs are polar, highly dynamic cytoskeletal fibers composed of tubulin subunits (Borisy and Taylor, 1967; Kirschner, 1978). There are two main classes of MTs, those attached to kinetochores and those that are not (Salmon et al., 1984). The non-kinetochore MTs can further be divided into inter-polar MTs, astral MTs, and possibly other types. A peculiar feature of the spindle apparatus in vertebrate cells is the poleward flux of MTs resulting from polymerization at the MT distal end and depolymerization at the MT ends proximal to the pole. Flux regulation by motor activity and/or MT-associated proteins is thought to control the mechanics and forces exerted on the chromatid pairs (Dietz, 1972; Inoué and Salmon, 1995). Some MTs have a propensity to get organized in bundles. They show two types of non-equilibrium dynamics, treadmilling and dynamic instability. Treadmilling (Hamaguchi et al., 1987; Oestergren, 1945) is the poleward flux of MTs toward their respective poles that occurs at the same rate as minus end depolymerization (Inoué and Ritter, 1975; Mitchison, 1989). The MTs also undergo alternating phases of growth and shrinkage at their plus end, termed dynamic instability (Mitchison and Kirschner, 1984). During metaphase, dynamic instability and flux create the delicate force balance that aligns chromosomes at the metaphase plate. During anaphase, chromosomes get segregated (Koshland et al., 1988). The equal distribution of DNA during division is an extremely crucial part of the cell cycle and its failure is a hallmark of disease. With the objective to inhibit cell division, the mitotic spindle is commonly targeted in oncology and our focus has been on the development of software for real-time image analysis (Matov, 2024j; Matov, 2024k) of high-resolution microscopy time-lapse series at different stages of the cell cycle.

### Spindle mechanics

The objective of our work has been to enhance the understanding of spindle mechanics (Klein, 1878) by mapping spatial and temporal variations of the flux patterns using quantitative fluorescent microscopy (Matov, 2024i; Matov, 2024j). These quantifications are used as a readout to make a distinction between the roles of different spindle-associated proteins in flux and, thus, force regulation (Matov, 2024f). Specifically, our contribution consists in developing a flow tracker (Matov, 2024k), together with computer vision and statistical tools for analyzing polymerization dynamics of filamentous actin (F-actin) (Adams et al., 2004; Ponti et al., 2003) and MTs during mitosis (Brennan et al., 2008; Matov et al., 2011). Fluorescent speckle microscopy (FSM) (Waterman-Storer and Salmon, 1998) allows the real-time analysis of subcellular dynamics as it relies on a low-labeling ratio of actin and tubulin dimers. In the mitotic spindle, clusters of four to seven fluorophores within the diffraction limit of the imaging system (Airy, 1835; Rayleigh, 1891), termed speckles, form a punctuate pattern on the MT bundles. The speckles serve as fiduciary marks for polymer flux (Matov, 2025b; Matov and Danuser, 2004; Matov et al., 2005; Ponti et al., 2003). The formation of speckles is a stochastic event based on the random incorporation of labeled and unlabeled subunits in the polymer lattice (Waterman-Storer and Salmon, 1998). Hence, to obtain reliable measurements of MT flux, one needs to analyze a vast amount of data and rely on a statistical representation. However, the high number of speckles, their anti-parallel motion during cell division, combined with low image acquisition rates due to photosensitivity of the fluorophores and the cells, makes the interpretation of such image time-lapse series difficult.

### Spindle analysis

To identify speckles among the numerous bright areas (blobs) that are convolved with imaging noise, we are mindful that the image signal frequencies are limited by the optical point spread function (Airy, 1835). For analysis, we retains features collected with a band-pass difference of Gaussians (Weierstrass, 1885) filter (Wilson and Giese, 1977), adapted to the camera specifications to satisfy the Nyquist criterion (Shannon, 1940), with a high spatial frequencies cut-off reflective of the microscope optics to suppress noise components (Matov et al., 2010; Ponti et al., 2005) and further exclude non-specific background fluorescent labeling associated with low-frequency noise components. The filtering and feature selection is complemented with least squares fitting (Holland and Welsch, 1977) of an *a priori* unknown number of blobs in the raw image against an average to remove outliers based on shape and intensity (Matov, 2024c). The software performs an automated extraction of speckle tracks and subsequent statistical analysis of the data (Matov, 2024f). The confounding problem in MT flux images is the anti-parallel speckle flow combined with high speckle proximity. We use graph theory – optimizing the network flow through the graph while minimizing the cost of linking – to obtain tracks forming coherent speckle flows (Matov, 2024c; Matov et al., 2011). This approach brings two advantages: (i) speckles can be tracked subject to certain (imposed by a self-adaptable cost function) rules of motion smoothness, resulting from the particularities of MT mechanics; (ii) speckles can be tracked under variable topological conditions, that is, in the case of sudden speckle appearance and disappearance, instantaneous change of flux direction or changes in the number of speckle flows (Gatlin et al., 2010; Matov, 2024f). This allows the evaluation of various micro-molecular and pharmacological treatments as well as the testing of specific biological hypotheses (Matov, 2024j).

### Interdigitated motion analysis

Speckles appear and disappear at any given moment, their number is not constant, and many of them are present in only one time frame. The only prior knowledge there is at hand is the fact that they all lay on MTs or MT bundles and an estimation of the approximate speckle speed (Maddox et al., 2003). Because of the complexity of the speckle patterns, we decided to consider three time frames at a time and take advantage of the knowledge of speckle coherent motion, that is, we apply a local motion model (Sethi and Jain, 1987). During the assignment process, each speckle is matched with potential candidates in the two following frames, thus creating speckle triplets that lay within a certain maximal search radius – to reduce computational complexity. Each link can be attributed a cost that represents the likeness of the match based on preservation of similarity between speckles in speed, motion directionality, and intensity. Because of the high number of short-living speckles, the key to speckle flow extraction is not to track all detected speckles in the images, but the optimal number of speckles. After forming all possible candidate triplets, a trade-off optimization procedure is to be used in order to maximize the number of links (Ahuja and Orlin, 1993) while minimizing the overall cost of those links at the same time (graph pruning), thus minimizing false positive assignments. Vector directional clustering allows determining the number of speckle flows and improving the tracks via an iterative, data-driven procedure based on a global motion model and spatial regularization for each flux direction (Matov, 2024c; Matov et al., 2011).

Extensive calibration and validation has been done of all tracking software modules by (i) manual benchmarking, (ii) benchmarking with known pharmacological perturbations, and (iii) artificial (synthetic) datasets. To compare automated speckle tracking with the gold standard of image analysis and interpretation, manual tracking, we developed a “manual tracker” module that has a graphical user interface (GUI) for user navigation. It allows manual tracking of speckles from appearance to disappearance by clicking on the computer screen while the program is saving the lists of selected image coordinates (see Materials and Methods for a download link and information on the manual tracking software). The users click on speckles in each frame of a time-lapse sequence, linking them to those in the previous. The software stores all data and compares it to the automatic tracking results. To validate our results, we also use image series of wild type cells and cells subjected to drugs and antibodies perturbing polymer dynamics with known effects. Some of these perturbations are performed, for instance, in the context of measuring slower flux and stopping flux by AMP-PNP (Brady, 1985) or monastrol (Mayer et al., 1999), which inhibit motor protein activity, and blocking dynamic instability by paclitaxel (Jordan and Wilson, 2004). Each software module is further benchmarked using artificial (or synthetic) image datasets for which the ground truth is known and the level of tracking difficulty can be modulated during data generation.

### Linear motion with occlusions analysis

Our contribution has been dedicated also to developing a technique (Yang et al., 2005) that reliably tracks a large number of image features undergoing dense linear motion. Such techniques are essential to the plethora of applications in fluorescence cellular and molecular imaging for reliable and accurate analysis of dynamic cellular functions. The basic tracking algorithm of this technique integrates motion models at feature local and global levels. It establishes correspondence between particles based on state similarity and resolves correspondence conflicts using bipartite graph assignment. A statistical and robust approach for algorithm parameter setting has been developed through establishing the equivalence of the algorithm to a Kalman (Swerling, 1959) filtering-based tracker under assumptions that are biologically supported. The linear motion tracking algorithm depends critically on computing the global vector field of feature flows, which is carried out iteratively using the optimal-flow minimum-cost graph algorithm described in the previous paragraph (Matov, 2024j; Matov et al., 2011). The computed spatially regularized instantaneous flow vectors also play an important role in reliably initiating full trajectories (Cameron et al., 2006; Yang et al., 2008; Yang et al., 2007; Yang et al., 2005).

Further, we created tracking software to extract information regarding the motion of MT plus ends by tracking the end binding protein 1 (EB1) (Slep and Vale, 2007), which recognizes conformational changes at the end of polymerizing MTs and, when fluorescently tagged (Piehl and Cassimeris, 2003), forms bright blobs, termed comets (Matov et al., 2010). Solving the MT end motion correspondence problem between frames in time-lapse series of about two hundred frames, each containing approximately two thousand comets, is a challenging task because the comets disappear during pausing and depolymerization. Our software allows mapping of polymerization and depolymerization events relying on comet appearance and disappearance only based on our observation that collinear comet trajectories likely belong to the same MT (Matov, 2006; Matov, 2009). This way, we can combine the extracted complete visible trajectories with an intensity fluctuation during comet appearance and disappearance as it relates to local polymer turnover (Matov et al., 2005) by making use of the fact that, in a lattice configuration, flux and turnover are interconnected. Again, we benchmark the polymerization and depolymerization maps under application of drugs with known effects on MT turnover and against manual tracking results.

## Results

### Microtubule minus ends

**FIGURE 1:**
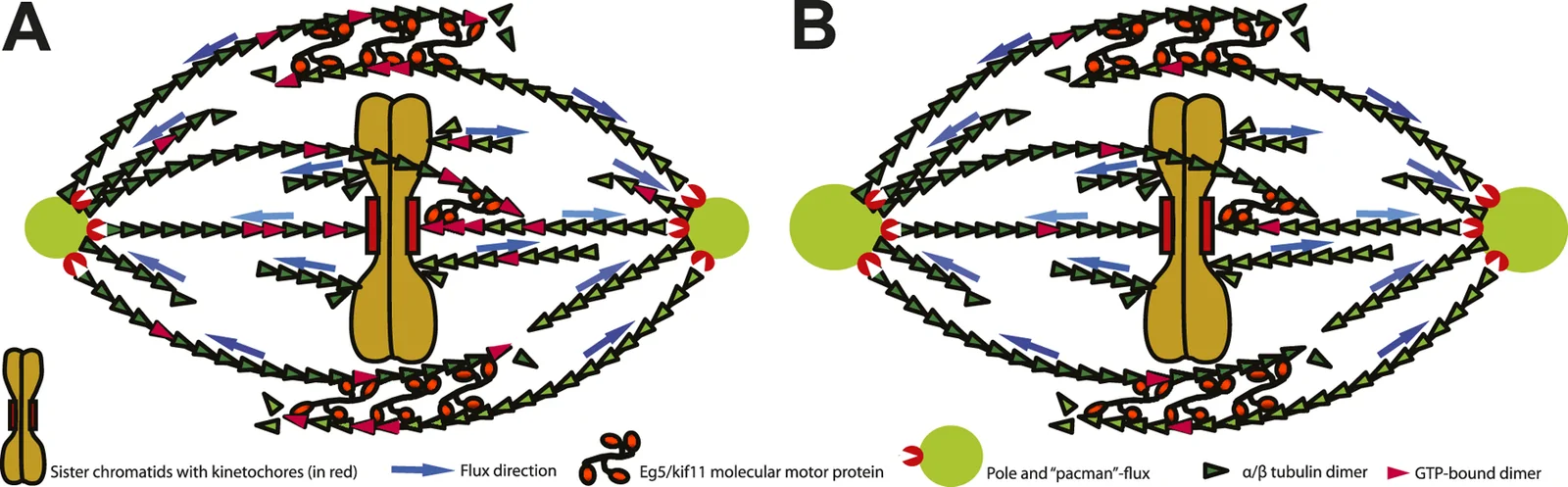
Differences in the dynamic organization of the mitotic spindle in regard to spindle pole size and frequencies of the GTP-remnants along the MT lattice and at the MT plus tips, that is, differences in the number of MTs that have a GTP cap. During metaphase, the sister chromatids are aligned at the metaphase plate by MT bundles attached to the kinetochores. Conversely, interpolar MT bundles overlap and are interconnected via plus end-directed molecular motor proteins. MTs have their minus ends toward the spindle poles, where they remove dimers, that is, depolymerize. MTs have their plus ends toward the metaphase plate, where they switch between states of polymerization and depolymerization, a process known as “dynamic instability”. **(A)** The figure depicts a cell in normal physiology for which some GTP remnants are present along the MT lattice and at MT plus tips (such MTs are termed GTP-capped) near the metaphase plate. The GTP remnants are often the sites of MT rescue, that is, the sites where depolymerizing MTs switch to a phase of polymerization. **(B)** The figure depicts a cancer cell in which fewer MTs are GTP-capped and there are less GTP remnants along the MT lattice. The spindle poles are unfocused and occupy a larger area. In such cells, the MTs are more likely to switch to depolymerization and less likely to pause or switch back to polymerization, which would result in segregation errors and improper chromosome segregation.

Spindle poles, where cytoplasmic and cortical dynein, and other proteins bring the MT minus ends together (Gatlin et al., 2009), become unfocused and enlarge their size in cancer. The centrosomes and proteins such as NuMA, dynactin (Gaglio et al., 1997), importin β (Ciciarello et al., 2004), severing enzymes (Zhang et al., 2007), and Aurora A (Ye et al., 2015) are located in the area of the spindle poles and contribute to the shortening of polar MTs (Figure 1). Inhibition of the pac-man mechanism (Cassimeris et al., 1987; Mitchison and Salmon, 1992; Mota, 1956) by the combined MT depolymerization and overall shortening at the MT minus ends can sensitize cells to tubulin inhibitors binding to individual dimers, such as vinorelbine (Mertens et al., 2023). Obtaining an approximation of the size of spindle poles by solving systems of linear Diophantine equations (Heger, 1858) for the pair-wise line intersections formed by the MT end trajectories head-to-tail (Houghtaling et al., 2009) can deliver metrics relating to the changes in regulation in cancer and drug susceptibility (see Materials and Methods for a download link and information on the spindle pole size calculation software).

We have identified as tentative drug targets multiple MT-regulating proteins that are dysregulated in prostate cancer (PC) and most of them are involved in the regulation of the MT minus ends (Tektin-2, MAP2, Op18, TACC3, CLIP170, TPX2, MAP1B, and MAP4) (Gao et al., 2014; Matov, 2025a). Our measurements (Gatlin et al., 2009; Matov, 2025a) indicate the importance of the activity of the minus end-directed molecular motor dynein and suggest the utilization of a dynein inhibitor (Höing et al., 2018) in addition to mitotic inhibitors in the clinic to inhibit secondary mechanisms of spindle assembly in patient tumor cells undergoing cell division. Further, the upregulation in cancer of half a dozen proteins (CLIP170, MAP1B, KIFC1, MAP4, CLASP2, and Aurora A) involved in the regulation of the MT minus ends might predispose tumor cells to necroptosis, similarly to the effects of the upregulation of Rac1 and Rho in cell lines (Matov, 2024g; Zhu et al., 2021), which suggests novel approaches to targeting resistant tumors.

### Models of pathogenesis and drug resistance

#### GTP remnants in the spindle

When MTs switch to a state of depolymerization, rescue events (or moments when they switch back to polymerization) occur predominantly at the sites of their lattice where incomplete GTP hydrolysis has occurred (Cassimeris, 2009; Dimitrov et al., 2008; Thoma et al., 2010). MT polymerize by adding GTP-bound tubulin dimers at their ends, which is followed by hydrolysis and the lattice consists of GDP-bound dimers (Hyman et al., 1992; Purich and Kristofferson, 1984). When we consider this scenario in the mitotic spindle during cell division, it is conceivable that the absence of GTP remnants (Figure 1; MT bundle formation is depicted in Supplementary Figure S1) on kinetochore MTs interferes with the function of the chromosome segregation machinery at the onset of anaphase. We have shown (Thoma et al., 2010) that in interphase MTs there are less frequent GTP remnants along the MT lattice and fewer MT ends are GTP-capped in cancer cells. When such cells undergo division that would result in partial impairment of the search and capture (Kirschner and Mitchison, 1986) or other mechanisms for chromosome attachment (Gudimchuk and McIntosh, 2021) and likely in aneuploidy, as some of the MT bundles have a plethora of depolymerizing at their plus ends MTs (Mitchison and Salmon, 1992) that pull and bring both sister chromatids to one of the poles.

Similarly, the inability of spindle MTs to switch back to a state of polymerization will create chromosomal instability and multipolar spindles (Matov et al., 2011), well-known hallmarks of cancer cells (Jallepalli and Lengauer, 2001; Therman and Timonen, 1950). A reduction in the frequency of GTP remnants in proximity to the plus ends of kinetochore MTs (see Figure 1) will inevitably lead to failure to align the kinetochores at the metaphase plate. Factors lowering the overall GTPase rate may increase the occurrence of GTP-tubulin caps at MT ends and GTP remnants along the MT lattice (Thoma et al., 2010), thus reducing the chances of improper chromosome segregation and aneuploidy. Factors increasing the overall GTPase rate may increase the changes of chromosome segregation errors and aneuploidy (Cuevas-Navarro et al., 2021), which predisposes to treatment with mitotic checkpoint inhibitors (Cohen-Sharir et al., 2021). The small GTPases are several fold smaller in size than the resolution limit of a spinning disk confocal microscope but live-cell imaging of labeled GTPases, such as RIT1 (Cuevas-Navarro et al., 2021), at super-resolution (Bond et al., 2024) will allows us to quantify the changes in subcellular localization and elucidate mechanisms of drug resistance.

Decades ago, Rap1, a small GTPase very similar to RAS, was observed to suppress oncogenic RAS phenotype and the proposed reason had been competition between RAS and Rap1 for a common, unidentified target (Kitayama et al., 1989). Further, in the context of metastasis, Rap1 and RhoA GTPase signaling reduces the size and inhibits the sliding of focal adhesions (FAs) in PC cells (Spanjaard et al., 2015), which mimic the effects of inhibition of RhoA-mediated cytoskeletal contractility, and promotes metastasis (Bailey et al., 2009). Cancer cells can switch between Rho/ROCK-mediated (Matov, 2024j; Matov, 2025b) contractility-based motility and Rac1-mediated (Matov, 2025a) mesenchymal migration. MTs locally modulate the activity of Rho GTPases, which initiate the polarization of the MT cytoskeleton in migrating cells (Wittmann and Waterman-Storer, 2001), while Rac1 has been shown to decrease MT catastrophe frequency and regulate MT dynamics at the leading edge (Wittmann et al., 2003). CLASPs preferentially bind the MT plus ends in the cell body, but bind along the entire MT lattice – mechanism regulated by Rac1 – in the lamella and lamellipodium (Wittmann and Waterman-Storer, 2005), which inhibits MT catastrophe at the leading edge. Our analysis has demonstrated that the density of polymerizing MTs is highest in the adhesion zone and reaches its peak at 3.5 µm from the cell edge. At distances of less than 3.5 µm to the cell edge, MT density is reduced (Matov et al., 2005) and their rates of polymerization are attenuated with 3.2 μm/min (Matov et al., 2006). Loss of Rap1GAP suppresses ROCK-mediated contractility and, instead, Rac1-mediated contractility enhances cell migration velocity and directionality (Tsygankova et al., 2013). FAs (Couchman and Rees, 1979) are tension sensitive structures that orchestrate cell migration (Winograd-Katz et al., 2014). Their size and maturation are controlled by vinculin at the ventral surface of living cells, which we quantified (Adams et al., 2004), and the contractility of the connected acto-myosin cytoskeleton. When contractility is reduced by partial inhibition of myosin II activity, the FAs shrink (Spanjaard et al., 2015) and eventually – once contractility is inhibited – disappear (Pasapera et al., 2010).

#### GTP to GDP hydrolysis

The cellular cytoskeletal filaments form as helical assemblies of subunits that self-associate, using a combination of end-to-end and side-to-side protein contacts. Both tubulin and actin subunits polymerize head-to-tail, thus creating polar filaments. Cytoskeletal protein complexes can be several dozens of microns long and reach opposite sides of a cell. However, the molecular subunits of the filaments are in the nanometers range. For this reason, cellular structures can undergo rapid structural reorganizations by depolymerizing filaments at one site while polymerizing elsewhere in the cell. Actin filaments are polymers of actin monomers, and MTs are made of α/β tubulin dimers. The non-equilibrium intracellular dynamics of both F-actin and MTs are largely determined by energy derived from ATP or GTP hydrolysis, and polymerizing MTs consist of GTP-bound tubulin dimers at their growing ends; hydrolysis follows, and the MT lattice consists of GDP-bound tubulin.

Resistance to tubulin inhibitors in lung cancer cells has been associated with increased MT dynamics (Gonçalves et al., 2001), which suggests dysregulation of MT-associated proteins (Matov, 2024e) and the presence of less GTP-bound tubulin (Thoma et al., 2010) or increased GTPase activity. RanGAP1 induces GTPase activity of Ran, but not Ras, while the mutant RanQ69L, which is unable to hydrolyze bound GTP as it is insensitive to RanGAP1, is locked in the active state (Bischoff et al., 1994). RanBP2/Nup358 and RanGAP1 are involved in the disassembly of nuclear export complexes in the cytoplasm (Ritterhoff et al., 2016), whereas a pro-oncogenic noncanonical activity of a Ras-GTP:RanGAP1 complex has been shown to facilitate nuclear protein export (Tripathi et al., 2024).

*Ras* genes are well established as the most frequently mutated oncogenes in human cancer, but difficult to target (Stephen et al., 2014). Almost one in five of all human cancers were shown to harbor a RAS alteration and *KRas* represents the majority (more than 80%) of mutated RAS in solid tumors, yet with the approved KRAS^G12C^‐GDP inhibitors only 12% of *KRas*-mutated tumors can be directly targeted and 36% of patients treated with sotorasib, for instance, experience either primary resistance or early disease progression after less than 3 months (Singhal et al., 2024). *Ras* is activated by GTP loading and deactivated upon GTP hydrolysis to GDP, that is, (ON) and (OFF) states, respectively. In epithelial tumor cells, the concentration of GTP is between 0.1 and 1 mM (Kopra et al., 2023), while the affinity of RAS for GTP is within the pM range (Bos, 2018). Therefore, a strategy of limiting the available pool of GTP molecules is applicable when the GTP concentration is significantly decreased, for example in quiescent cells that are starved (Matov, 2024e).

To this end, drug combinations that maintain MT polymerization (like the taxanes and epothilones) and inhibit switches to depolymerization or pausing could be utilized together with compounds that induce an activity of MT severing enzymes (the ATPases katanin, spastin, and fidgetin) (Kuo and Howard, 2021). MT severing proteins catalyze tubulin exchange along the MT lattice and, thus, patch up the new-formed GDP-bound MT ends with GTP-bound tubulin (Vemu et al., 2018). Phosphorylation of Op18 (Cassimeris, 2002; Tournebize et al., 1997) and/or the inhibition of KIF2C (Manning et al., 2007) would reduce the frequency of catastrophe as well as increase the sensitivity to taxane treatment (Mistry and Atweh, 2006; Pao et al., 2025). Treatment with the histone deacetylase trichostatin A (TSA) would increase the MT polymerizing plus pausing times (that is, the overall time MTs spend before switching to depolymerization) (Matov, 2025a). This way, by considering the combined effects on MT dynamics induced, we could identify a drug combination that maximizes the availability of polymerizing MT ends recruiting GTP molecules and minimize their availability to the RAS GTPases. The quantitative live-cell imaging approaches for the evaluation of MT dynamics we present here can, thus, help refine and personalize the treatment regimen based on the *ex vivo* analysis of patient-derived cells.

### Analysis of microtubules in drug resistance

#### Analysis of the cytoskeleton

We use our computational approach to measure MT dynamics signatures (a panel of a dozen parameters that comprehensively describe MT dynamics) (Applegate et al., 2011; Matov, 2024e; Matov, 2024h; Matov, 2024j; Matov et al., 2010; Matov et al., 2013) and correlate changes in the signatures that result from patient treatment with therapeutics. Traditionally, MT dynamics (Brouhard and Rice, 2018) have been evaluated by means of manual tracking (recording x,y coordinates in XLS files for each frame over the time-lapse series) and kymograph analysis, where a band of pixels around the desired MT end is stitched onto a single panel, thus aggregating the evolution of image pixel intensities over the time-lapse sequence (see Material and Methods for software for automated kymograph analysis). Manual analysis normally covers about 10-20 MTs and because of the high density of the MT network, MTs are selected from a very narrow region of the cellular lamellipodia proximal to the cell edge, where MT dynamics do not represent the overall behavior of MTs in the cell (Burakov et al., 2021; Komarova et al., 2009; Kumar et al., 2009; Matov et al., 2010; Mimori-Kiyosue et al., 2005; Vorobjev et al., 1999). In contrast, our software allows measurement of MT dynamics throughout the cell body and delivers statistically large datasets in the hundreds of thousands of quantitative readout points. These large datasets increase the sensitivity of the assay, allowing us to detect very small, yet significant changes in the MT dynamics signatures. Furthermore, real-time image analysis and instant quantitative feedback will significantly speed up research work, allow precise calibration of the imaging setup, and successful optimization of the drug regimens. To achieve this, we have developed real-time computer vision software (Matov, 2024j; Matov, 2024k) that can connect to the microscope’s camera, process multiple image frames per second, and instantly display and store statistical readouts in parallel to image acquisition. Our software is optimized for resource-constrained computing and can be installed even on microscopes without internet connectivity (Matov, 2024k). We will extend its functionality to deliver additional morphology and motion metrics (Matov, 2024b).

#### Tubulin inhibitors

The MT cytoskeleton is often targeted during treatment of solid tumors (Jordan and Wilson, 2004; Mukhtar et al., 2014) – for instance, in PC (Martin et al., 2014) – and the MTs are one of the most validated targets in oncology (Jordan and Wilson, 2004; Rao et al., 2012) due to the efficacy of taxanes, vinca alkaloids, and other tubulin inhibitors in a wide range of cancers. Some tubulin inhibitors bind to individual tubulin dimers and block polymerization, other bind to the lattice of MTs and block the possibility of the MTs to depolymerize, like the taxanes. For some cancers several tubulin inhibitors are used clinically (e.g., taxanes and vinca alkaloids), suggesting that targeting the MT cytoskeleton is impactful, yet there is large variability in patient response. A plethora of models for the mechanics of drug resistance have been proposed (Kavallaris, 2010; Orr et al., 2003), the most common of which are: (i) resistance due to membrane-pump overexpression (Robert, 1999), (ii) resistance due to tubulin-isotype composition (Haber et al., 1995; Kanakkanthara et al., 2011; Ranganathan et al., 1998), (iii) resistance due to mutations in drug binding sites (Giannakakou et al., 2000a; Giannakakou et al., 1997) as well as (iv) resistance due to changes in regulatory microRNAs (Kanakkanthara and Miller, 2012) or other types of cytoskeletal modulation (Wang V. E. et al., 2017). Evidence for these models stems largely from work with tissue culture cancer cell lines. Because these lines fail to adequately represent the genetic diversity found in the patient population, it is unclear how these findings relate to resistance in patient tumors (Matov et al., 2016).

Our work aims to elucidate the links between the effects of tubulin inhibitors on their targets’ dynamics in patient-derived cells and the efficacy of treatment of solid tumors. We have focused our investigation on 70 MT-associated proteins that regulate various stages of MT homeostasis (Lyle et al., 2009a; Lyle et al., 2009b). Variations in these genes’ expression levels have been linked to which tubulin inhibitors affect cells and whether or not a drug is able to kill the cancer cells (Albrethsen et al., 2014; Berges et al., 2014; Devred et al., 2008; Le Grand et al., 2014; Li and Febbo, 2013; Li et al., 2014; Malesinski et al., 2015; O'Rourke et al., 2014; Thomas et al., 2015; Xie et al., 2015).

#### IC_50_ analysis

**FIGURE 2:**
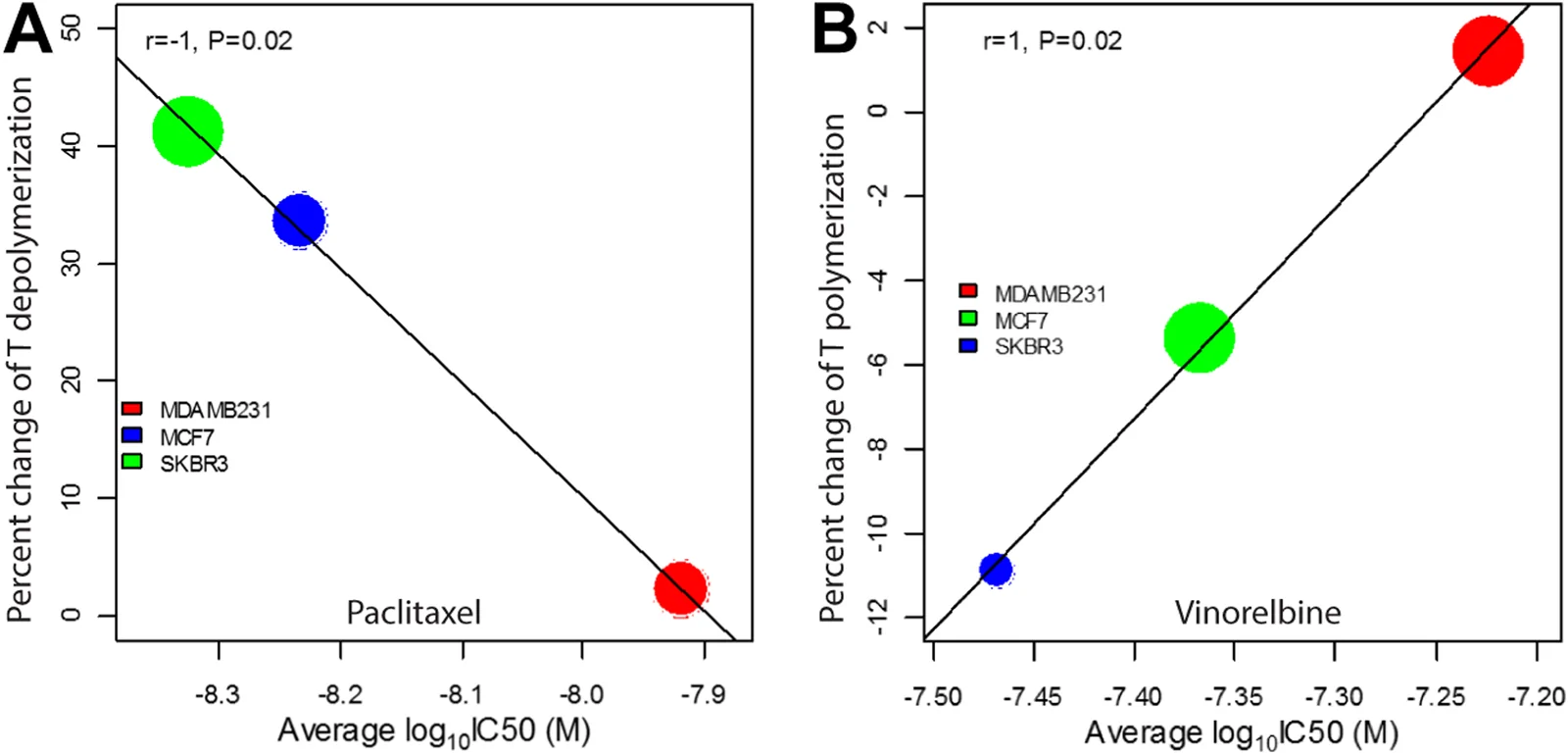
Correlation between IC_50_ values and MT dynamics parameters in BC cells. Three BC cell lines (triple-negative cells (MDA-MB-231), ER-positive cells (MCF-7), and HER2-positive cells (SK-BR-3)) were treated with a taxane (0.5 nM paclitaxel) and a vinca alkaloid (0.1 nM vinorelbine), and parameters of MT dynamics were measured (Matov et al., 2010) for 20 cells/movies per condition. For the same cell lines and drugs, three to six IC_50_ experiments were performed to obtain a representative value. **(A)** Reverse correlation between the increase in resistance to paclitaxel with the percent change in the relative time MTs spend depolymerizing. For the sensitive SK-BR-3 and MCF-7 cells, the increase in this parameter is coupled with a decrease in the pausing times (without changes in other related parameters), indicating increased frequencies of switching from pausing to polymerization (Matov, 2024h). **(B)** Correlation between the increase in resistance to vinorelbine with the percent of change in the time MTs spend polymerizing. For the sensitive SK-BR-3 and MCF-7 cells, the decrease in this parameter is coupled with an increase in the probability of switching to depolymerization at the end of a polymerization track (rather than to pausing), indicating an increase in the catastrophe frequencies. While the time spend polymerizing did not change for the resistant MDA-MB-231 cells, the probability of switching to depolymerization at the end of a polymerization track (conversely) increased, indicating a decrease in the catastrophe frequencies (Matov, 2024h). These new results demonstrate a proof-of-principle that MT dynamics can predict resistance to tubulin inhibitors in pre-clinical data.

We sought to validate the ability of our approach (Matov, 2024h) to anticipate resistance to tubulin inhibitors in comparison to established methods, such the IC_50_ value. Our measurements in breast cancer (BC) cells demonstrate the utility of MT dynamics in determining drug resistance. The receptor triple-negative (MDA-MB-231) cells were the most resistant, the ER-positive (MCF-7) cells had intermediate IC_50_ values, and the HER2-positive (SK-BR-3) cells were the most sensitive. For a taxane (Figure 2A), an increase in the IC_50_ values (that is, the cancer cells being more resistant to treatment with 0.5 nM paclitaxel) correlates in the two sensitive cell lines with an increase in the relative time MTs spend depolymerizing. Since the times of pausing were decreased as well, while the remaining parameters relating to the state switching frequencies were not affected, the changes in these two parameters indicate increased frequencies of switching from pausing to polymerization. These new results suggest that not the canonical rescue frequency, but the increased frequency of switching from pausing to polymerization is a MT dynamics parameter predictive of paclitaxel efficacy, which reveals a novel function of paclitaxel.

For a vinca alkaloid (Figure 2B), an increase in the IC_50_ values (that is, the cancer cells being more resistant to treatment with 0.1 nM vinorelbine) correlates with an increase in MT polymerization times, which is reversely correlated with the frequency of switch to depolymerization (termed catastrophe frequency). For the sensitive SK-BR-3 and MCF-7 cells, the time MTs spend polymerizing decreased. This change was coupled with increased probability of switching to depolymerization at the end of a polymerization track, rather than to pausing. The combined changes in these parameters indicate increased catastrophe frequency. Conversely, for the resistant MDA-MB-231 cells, the time MTs spend polymerizing did not change (Matov, 2024h). This change was coupled with decreased probability of switching to depolymerization at the end of a polymerization track, rather than to pausing. The combined changes in these parameters indicate decreased catastrophe frequency. These new results suggest that the increased catastrophe frequency is a MT dynamics parameters predictive of vinorelbine efficacy, but also demonstrate a novel function of vinorelbine.

Taken together, our analysis results demonstrate a proof-of-principle that MT dynamics can predict resistance to tubulin inhibitors in pre-clinical data.

In colorectal cancer (CRC) cells, paclitaxel treatment has been shown to, by attenuating MT polymerization rates, lead to overcoming chromosomal instability in tumor cells and successfully progressing through mitosis (Ertych et al., 2014). We elucidate in this contribution a new function of paclitaxel related to an increased frequency of switching from MT pausing to polymerization, which – rather than the attenuation of MT polymerization rates – could be utilized to predict paclitaxel efficacy. In BC cells, our measurements of MT dynamics (Matov, 2024h) have indicated that receptor-negative tumors, for which there are no efficient treatment options (Maqbool et al., 2022), exhibit tumor-specific MT dynamics (Matov, 2024g) and might be tentatively susceptible to vinorelbine rather than paclitaxel during treatment of BC (Matov, 2024h).

Doses and schedules of oncology drugs are sometimes inadequately characterized and the “more is better” paradigm is still used for dose selection, despite the recognition of a need for dose optimization (Shah et al., 2021). Our hypothesis is that treatment with low drug doses can reveal shifts in the values of specific MT parameters (Matov, 2024h) indicative of drug efficacy.

Vinorelbine has previously been shown to attenuate MT polymerization rates, increase MT polymerization times, and reduce the MT depolymerization times (Ngan et al., 2000). We elucidate in this contribution a new function of vinorelbine related to decreasing MT catastrophe frequencies, which – rather than parameters related to MT destabilization – could be utilized to predict vinorelbine efficacy. Our work demonstrates that, at low doses (0.1 nM), it increases the rates of MT turnover and decreases the catastrophe frequencies in all, but ER-positive cells (Matov, 2024h). Furthermore, treatment with 0.1 nM vinorelbine reduces the percent time MTs spend depolymerizing in receptor-negative BC cells (Matov, 2024h), which increases the frequency of MT rescue. These new result suggests that, despite vinorelbine’s classification as a MT-destabilizing drug, it also exhibits properties intrinsic to the taxanes, which can be the basis for a clinical investigation of vinorelbine as a first-line chemotherapy in receptor triple-negative BC (TNBC).

Vinorelbine (and partially docetaxel) has been shown to be selectively toxic to *BRAF*-like CRC cells, but not to *KRas-BRAF* CRC cells, that exhibit a reduced kinetochore MT polymerization (Vecchione et al., 2016). In addition, suppression of RanBP2/Nup358 (Joseph et al., 2004) in *BRAF*-like CRC cells perturbs spindle formation, prolongs the time spent in mitosis, triggers multiple mitotic defects, and, ultimately, induces cell death during or immediately after cell division (Vecchione et al., 2016). The off-label use of another vinca alkaloid, vinblastine, in resistant *BRAF*-like CRC has been shown to induce long-term complete remission (Linn et al., 1994). The size of Nups is several folds smaller than the resolution limit of a spinning disk confocal microscope, whereas super-resolution live-cell imaging (Bond et al., 2024) of labeled Nups will allows us to quantify the changes in bidirectional trafficking and elucidate mechanisms of drug resistance.

Vinorelbine has also been shown to be consistently effective across a panel of more than 25 different CRC patient-derived organoids, independent of RAS mutational status. Unlike vinorelbine alone (which was effective mainly during mitosis), its combination with EGFR and MAPK inhibition induced apoptosis at all stages of the cell cycle – but predominantly during G1 phase – and showed tolerability and effective anti-tumor activity *in vivo*, setting the basis for a clinical trial to treat patients with metastatic RAS-mutant CRC (Mertens et al., 2023). Quantitative live-cell imaging of MT dynamics in patient organoids can elucidate mechanisms of acquired resistance to vinorelbine.

#### Drug discovery

Our computational approach has revealed novel organizational and regulatory patterns in MT homeostasis in cancer (initially in renal cell carcinoma (Thoma et al., 2010)) and has begun to impact studies on a wide range of topics in cancer biology (Braun et al., 2014; Ertych et al., 2014; Galletti et al., 2014; Garcez et al., 2015; Harkcom et al., 2014; Kwon et al., 2015; Long et al., 2013; McGrail et al., 2015; Patel and Chu, 2014; Sironi et al., 2011; Smole et al., 2014; Tegha-Dunghu et al., 2014; Wu et al., 2011). Our objective is to elucidate the various mechanisms of response to drug action by (i) classifying the MT dynamics signatures based on patient treatment outcome to investigate individual mechanisms of resistance to tubulin inhibitors (Matov, 2024h; Matov, 2024j). Furthermore, by measuring the signatures before and after drug treatment, we could (ii) identify the most affected parameter of MT dynamics and analyze the corresponding patient-specific pattern of differentially expressed MT-modulating genes to identify novel personalized targets for sensitizing resistant tumors to MT-targeting therapies (Matov, 2024e; Matov, 2024f). Our goal is the establishment of patient-specific MT dynamics signatures as indicators of oncogene-driven resistance in metastatic tumors, and the identification of personalized tumor-associated predictors of treatment response. Such an evidence-based strategy to analyze drug action at the cellular level will offer a quantitative readout of cell susceptibility to drug action, which, in turn, will allow to discover mechanisms of drug resistance and contribute to drug discovery. Elucidating individual mechanisms of resistance to therapy has the potential to improve cancer treatment by establishing a personalized medicine approach.

### Microtubule regulation in pathogenesis and drug toxicity

#### Regulation in disease

During pathogenesis, or as a result of drug treatment in disease, cells change their intracellular organization, rearrange their internal components as they grow, divide, and adapt mechanically to a hostile environment (Matov, 2024e). These functions depend on protein filaments (cytoskeletal polymers and FAs), which provide the cell shape and its capacity for directed movement. In this context, our focus has been on the automated analysis of cytoskeletal dynamics, predominantly those of MTs (Brennan et al., 2008; Gatlin et al., 2010; Gatlin et al., 2009; Harkcom et al., 2014; Houghtaling et al., 2009; Matov et al., 2013) and F-actin (Matov, 2024f; Ponti et al., 2005), to study the cellular effects of tubulin, actin, and tropomyosin inhibitors and post-translational modifications as well as modulation of FAs (Adams et al., 2004; Spanjaard et al., 2015), motor proteins (Gatlin et al., 2009; Houghtaling et al., 2009; Matov, 2024f), and the ectopic activation of GSK3β (Kumar et al., 2009). This kinase regulates the cytoskeleton and multiple MT-associated proteins (Hajka et al., 2021), and numerous drugs targeting it are under development by the pharmaceutical industry for the treatment of a wide variety of diseases (Kazi et al., 2018; Kingwell, 2018; Wood, 2022). Our goal is to identify disease-specific measurable alterations in subcellular regulation that could serve as cues for informed decisions for personalized medical treatment by anticipating pathogenesis or drug resistance at the cellular level as well as offer novel strategies for targeting therapies to sensitize resistant disease.

#### Exceptional responders

It is not understood why traditional tubulin inhibitors fail in the clinic so often. We are interested in elucidating cellular mechanisms that lead to complete response to drug treatment after which tumors recede completely. In some cases, treatment leads to a complete and lasting remission after only a few doses (Linn et al., 1994), which also cannot be predicted. Signaling between tumor cells may result in a domino effect, termed a bystander effect in radiotherapy (Mitchison, 2012), causing tumors to recede significantly after a minimal intervention. Dissecting the mechanisms of drug action with quantitative detail will allow the design of novel drugs that impede relapse and the selection of optimal dose regimens with minimal harmful side effects by exploiting disease-specific cellular aberrations. Our hypothesis is that mutations in cancer-causing genes can be linked to a particular set of deviations in MT (and subcellular) regulation and dynamics. Therefore, candidate drug regimens could be tested *ex vivo* for their ability to revert the concrete defects, neutralize the disrupting impact of the disease, and sensitize it to a therapy regimen with low toxicity. Validation as both a prognostic and a predictive assay can be done by comparing the *ex vivo* tumor growth kinetics and treatment predictions to outcome of patients with similar genetic profiles in clinical trials (Matov, 2024j). Sequencing analysis will also be matched to available tumor tissues from databases with genomic data. We hypothesize that we can (i) identify an exceptional responder per each molecular subtype (that is, a regimen resulting in a complete response) and (ii) sensitize every tumor to one of the exceptional response regimens by modulating cytoskeletal dynamics and cellular organization.

Based on *in vitro* experiments, nuclear localization sequences, EB1, and CKAP5/XMAP215 are required for MT assembly (Groen, 2008). Three nucleation factors, γ-tubulin, TPX2, and CKAP5/XMAP215 are required for MT polymerization and spindle assembly around chromatin beads (Groen et al., 2009). Depletion of TPX2 (which mediates new nucleation on top of pre-existing MTs (Petry et al., 2013)) is partially rescued by adding EB1 or CKAP5/XMAP215 (Gard and Kirschner, 1987) or by inhibiting the mitotic centromere-associated kinase KIF2C/MCAK (a kinesin-13, regulating MT depolymerization rates (Linda, 2005; Wang W. et al., 2017)). Depletion of γ-tubulin can also be partially rescued by adding CKAP5/XMAP215, suggesting it has a particularly important role in spindle assembly. We investigated the functionality of the MT polymerase CKAP5/XMAP215 using artificial MT organizing centers encapsulated in droplets of *Xenopus egg* extracts and measured that the MT polymerization rates (Supplementary Video S1) scale linearly with cytoplasmic volume. Interestingly, the increase of the concentration of CKAP5/XMAP215 allowed large spindles to form in very small volumes (Milunović-Jevtić et al., 2015). Cancer cells with large spindles are often associated with aggressive disease and drug resistance (Matov et al., 2015), and are often targeted with tubulin inhibitors that lead to harmful side effects in off-target tissues.

Treatment with all tubulin inhibitors, but colchicine, leads to neutropenia (low neutrophil count) as a side effect and new variations of these compounds are required – drugs that combine the pharmacokinetics of colchicine with the pharmacodynamics of traditional tubulin inhibitors. Drugs like colchicine, which has almost no side effects at low doses (Dinarello et al., 1976) and is suitable for lifelong treatment, are accumulated in the liver and kidneys (Thomas et al., 1989) (as opposed to the accumulation in neutrophils and other leukocytes of most traditional tubulin inhibitors), because of the very low dissociation constant and unusual pharmacokinetics, making kidney organoids obtained from patient urine samples (Gijzen et al., 2021; Yousef Yengej et al., 2020) a suitable system for disease assessment and toxicity testing. Our computational approach will provide high-content analyses and functional secondary screening of novel compounds that are in the process of approval or at a preclinical stage of development and new combination therapies based on FDA-approved tubulin inhibitors (Matov, 2024i; Matov, 2024j; Matov, 2024k).

### Targeting cancer cell-specific state and vulnerabilities

#### Urinary biomarkers

Our analysis of healthy donor samples and drug-naïve lung cancer patients’ samples has identified urinary biomarkers demonstrating that small RNAs can pass through the filtration by the kidneys. We have provided a proof-of-principle that it is possible to perform non-invasive health monitoring by sequencing of urinary small RNAs and that traces of neoplastic transformation originating in organs that are not adjacent to the urinary tract, like the lungs, can also be detected in urine (Matov, 2024l). We performed longitudinal analysis of urinary microRNAs with the objective of monitoring gradual changes in their regulation and abundance levels toward disease values (Matov, 2024b). Therefore, monitoring of microRNA levels in healthy individuals and patients undergoing disease treatment can provide valuable datasets for the pharmaceutical industry (Jurj et al., 2024) as well as for practicing physicians (Martino et al., 2025), ultimately allowing them to select the most efficacious treatment sequence and drug combinations for each patient (Chen, 2023).

**FIGURE 3:**
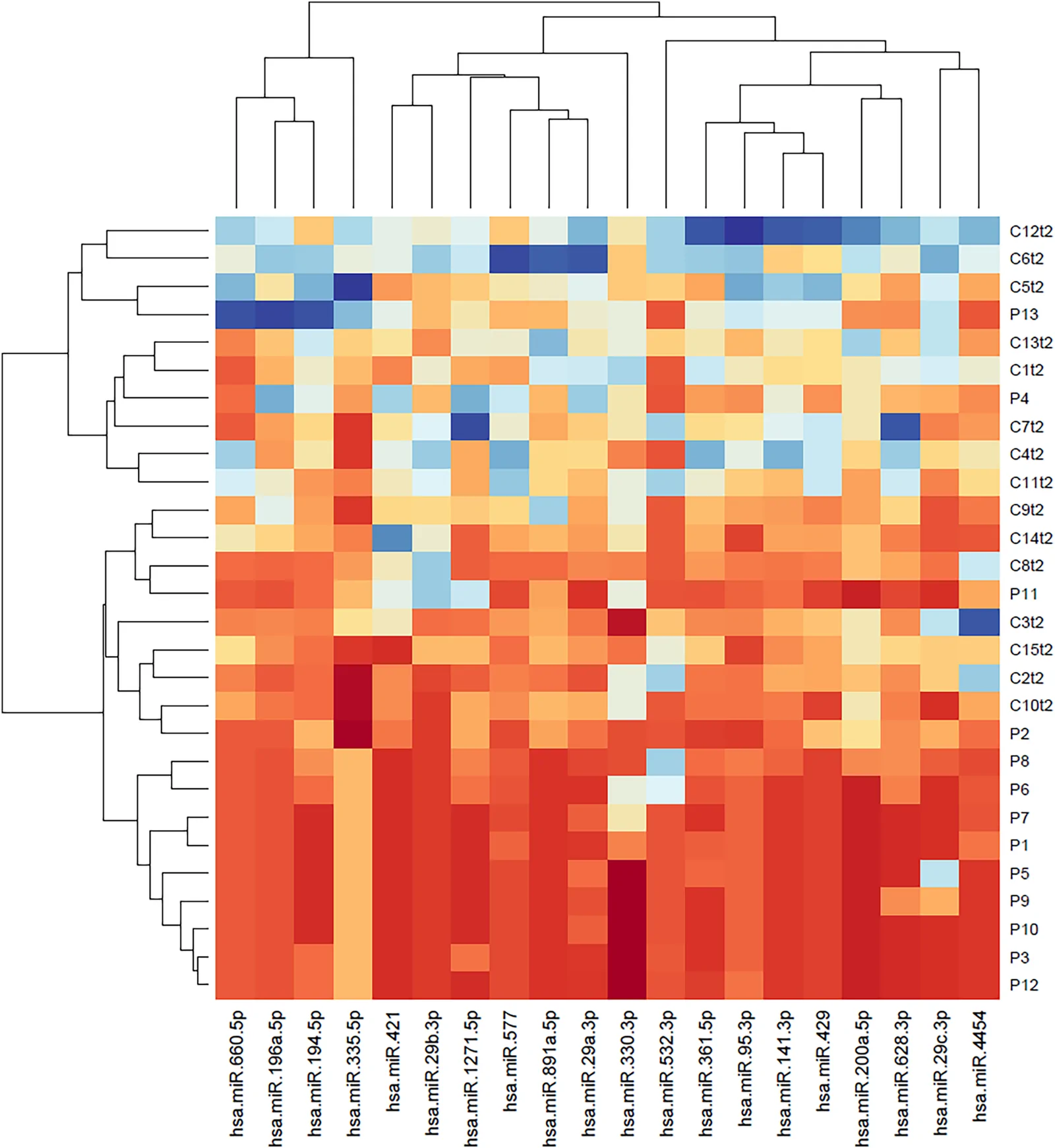
Hierarchical clustering dendrogram of urinary microRNAs in samples from lung cancer patients and healthy individuals. A cohort of 28 urine samples was analyzed by computation of abundance levels‘ Kullback-Leibler divergence relative entropy (Matov, 2024l). This analysis allowed for the identification of 20 microRNAs (out of the 947 microRNAs we detected by next-generation sequencing in urine) to discriminate between 13 stage IV drug-naïve lung cancer patients (P1-P13) and 15 healthy or control individuals (C1t2-C15t2, where t2 indicates time point 2). All 20 microRNAs have previously been published based on analyses of primary lung tumors and blood from lung cancer patients. In addition, some of the biomarker have been implicated in MT and pVHL regulation, and the modulation of mitochondrion function.

Patient urine samples can also deliver additional biomarkers for non-invasive detection of disease and drug resistance (Kim and Croce, 2023). We identified 20 urinary microRNAs as biomarkers (Matov, 2024l), which can be utilized for patient stratification (Figure 3) and can serve as tentative targets for lung cancer treatment. Some of the biomarkers have been implicated in MT and pVHL regulation, and the modulation of mitochondrion function (Bao et al., 2018; Wan and Zheng, 2021; Wang et al., 2014). The most discriminative of these 20 biomarkers, microRNA-532-3p (for which we measured low abundance levels in urine samples from lung cancer patients), has a role during tumor growth and has been shown to have a tumor suppressor role in lung cancer (Jiang et al., 2019), and could be targeted during treatment. Our results demonstrate the possibility of performing non-invasive testing for lung cancer (which is the most lethal cancer and is very difficult to detect in its early stages, unless a dedicated screening based on longitudinal patient data is performed (Matov, 2024d), because of the lack of symptoms) and we have performed work in the context of establishing lung organoids.

**FIGURE 4:**
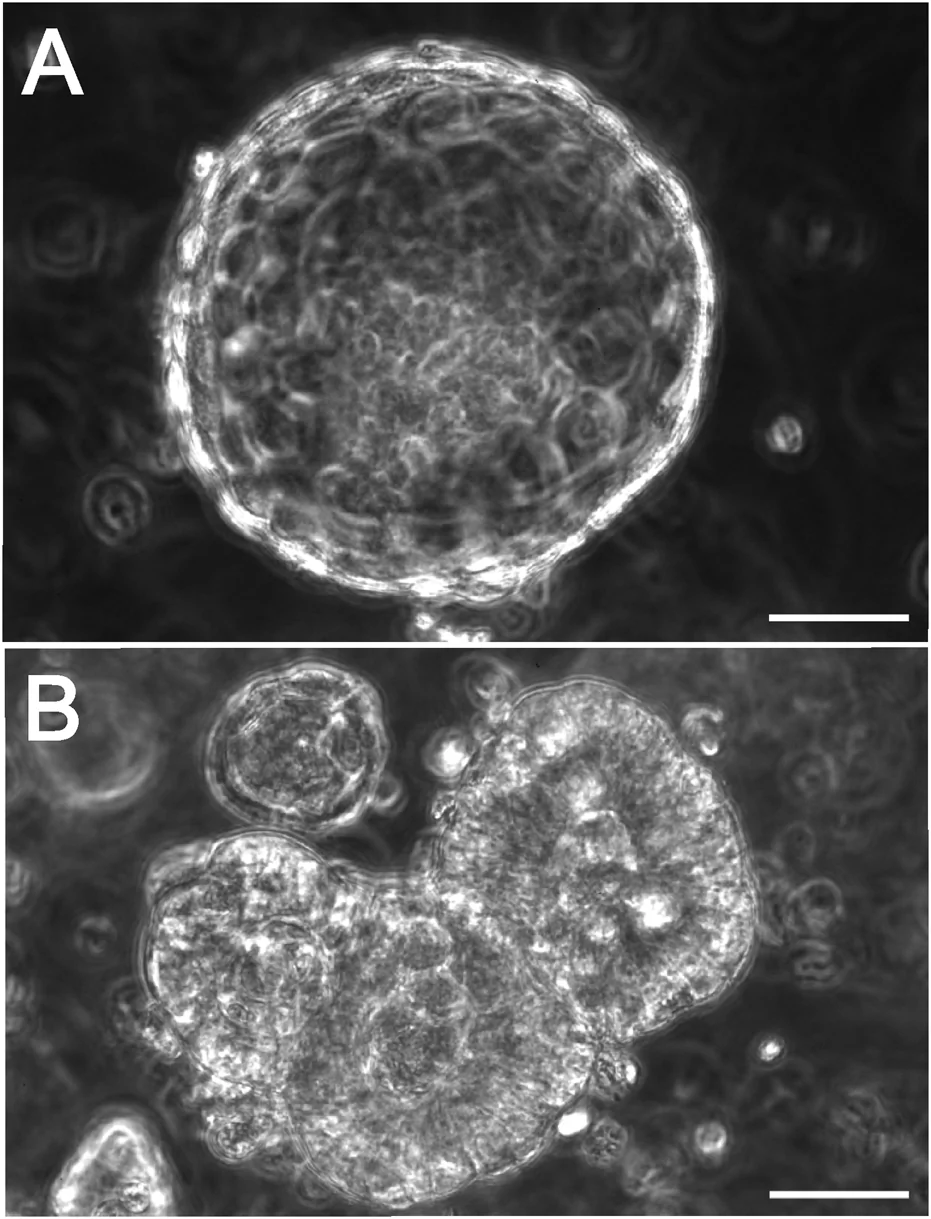
Organoids derived from a 70 mg chest-wall resection of a patient with metastatic rectal cancer. Day 25. **(A)** Lung organoid. **(B)** Rectal organoids; form crypts. Magnification 20×, phase contrast microscopy. Scale bars equal 50 µm.

Figure 4 shows a lung organoid derived from a chest-wall resection of metastasized rectal tumor protruding from the sternum. The organoid culture system allows the development of organ-specific models of disease progression and drug resistance (Sachs and Clevers, 2014). When tissues from different organs mix, organoids from both types of cells can be cultured (in this case, also rectal organoids). The combined utilization of small and long RNA sequencing with the quantitative imaging of live-cell intracellular dynamics (Matov, 2025a) and growth kinetics (Matov, 2024j) of patient organoids can provide a very detailed assessment in regard to disease prognosis and drug susceptibility. While tubulin inhibitors are not approved in lung cancer, it is likely that a subset of patients is susceptible (Figure 3).

Clinical data from RMC-6291 (Schulze et al., 2023), a KRAS^G12C^(ON)-only inhibitor that depends on a tri-complex formation with cyclophilin A, has recently reported an objective response rate of 50% in patients with non-small cell lung cancer undergoing recent prior KRAS^G12C^(OFF)-only inhibitor treatment (Jänne et al., 2023). Acquired drug resistance limits the clinical impact of targeting RAS with tri-complex inhibitors that recruit cyclophilin A to the active (ON) conformation of RAS. One of the resistance mechanisms is acquired MEK1 mutations (Sang et al., 2025). The activity of MEK1 destabilizes interphase MTs and reduces mitotic spindle defects (Tolg et al., 2010). Furthermore, cyclophilin A binds cytoplasmic dynein and co-localizes with MTs (Galigniana et al., 2004), suggesting that quantitative live-cell microscopy analysis of MT dynamics can facilitate the optimization of lung cancer therapy.

#### Drug-tolerant cells

Even when tubulin inhibitors are successful in reducing the tumor burden, a fraction of the tumor cells change their metabolic state and become quiescent to avoid treatment. We envisage using drug-tolerant tumor cells (persister cells) derived from patient organoids as a drug discovery platform to identify therapeutic agents that can eliminate residual disease remaining after initial successful cytotoxic drug treatment (Matov, 2024b). One such example is targeting the change of cell state in drug-tolerant persisters (He et al., 2024; Russo et al., 2024). Ferroptosis is known to have a dual role in cancer – in tumor initiation, which depends on inflammation-associated immunosuppression triggered by damage from ferroptosis (Chen et al., 2021), and later in tumor suppression. Analysis of cfDNA in patient blood samples identified dysregulation in the antioxidant program as a possible biomarker of subclinical and early-stage CRC (Matov, 2024a). The inability to tolerate lipid hydroperoxide accumulation renders cancer cells with a disabled antioxidant program susceptible to ferroptosis (Hangauer et al., 2017). Mitochondria have an antioxidant system that counteracts the accumulation of reactive oxygen species (Dixon and Stockwell, 2014) and prevents ferroptosis (Garcia-Bermudez and Birsoy, 2021), which might lead to new anticancer therapies (Mao et al., 2021). About half of the mitochondria are stationary or pausing, that is, not moving (Matov, 2024j). The motile mitochondria spend about 70% of the time pausing if they undergo anterograde motion and 73% of the time pausing if they undergo retrograde motion along axons on MTs. Importantly, as fusion and fission of mitochondria is impaired in disease (Nabi et al., 2022), morphology and texture analysis (Murphy et al., 2003) can be applied during *ex vivo* drug susceptibility testing (Matov, 2024j).

#### Drug selection

Our previous work with cancer cell lines has demonstrated that we can associate the alterations in particular parameters of MT dynamics to drug resistance (Galletti et al., 2014; Matov, 2024e; Matov, 2024h; Matov et al., 2013). Moreover, these analyses demonstrate a proof-of-principle that it is possible to pinpoint the MT dynamics signatures that reveal tubulin inhibitors’ efficacy. We can now start identifying the proteins (and pathways) that regulate them and investigate their relative expression in patient-derived organoids, as cultures with high clinical relevance (Matov, 2024j; Matov et al., 2016). Because different tubulin inhibitors affect distinct parameters of MT dynamics, it is conceivable that we can match each drug with an individual tumor-specific MT dynamics signature to maximize therapeutic efficacy (Matov, 2024e; Matov, 2024h; Matov et al., 2013). To achieve this goal, we have used this approach to match existing and predict new genotypes-phenotypes associations in renal cell carcinoma (Thoma et al., 2010), PC (Galletti et al., 2014), and BC (Matov, 2024h), demonstrating it is suitable for our search for drug-disease associations in solid tumor oncology. Our algorithm can further be applied for evaluation of drug candidates’ ability to exploit cancer-specific defects in MT homeostasis. Such computational studies have the potential to impact clinical decision-making by equipping physicians with a computer-aided tool (Matov, 2024j) allowing the design of an effective personalized medical treatment. Our long-term goal is to tailor therapy to each individual patient according to the tumor-specific alterations in regulation in order to transform metastatic cancer into a chronic, manageable disease.

### Tumor sensitizing and drug susceptibility

#### von Hippel-Lindau mutations

Point mutations in the *VHL* gene lead to various patterns of MT dynamics signatures (Thoma et al., 2010). pVHL regulates the activity of HIF1α (Genbacev et al., 2001; Kaelin and Ratcliffe, 2008; Maxwell et al., 2001; Staller et al., 2003) and *VHL* mutations lead to a variety of malignant and benign tumors of the eye, brain, spinal cord, kidney, pancreas, and adrenal glands. Hypoxic microenvironment drives the selection of aggressive cancer cells through F-actin cytoskeletal polarization, downregulation of E-cadherin, and epithelial-mesenchymal transition (Buart et al., 2021). Mutations in *VHL* lead to an increased GTP hydrolysis (Thoma et al., 2010) and dysregulated MT dynamics (Barry and Krek, 2004), a decreased drug susceptibility, and increased oncogenic activity. Dysfunctional pVHL tumor suppressor leads, for instance, to the upregulation of the MT-associated protein MAP1, which has been implicated in pathogenesis (Czaniecki et al., 2019; Fifre et al., 2006) as well as drug resistance in several cancers (Laks et al., 2018) and, thus, represents a target within the MT transcriptome to sensitize resistant disease. We demonstrated (Thoma et al., 2010) that it is possible to associate dysregulation of specific aspects of MT dynamics to point mutations occurring within a particular domain of pVHL. Some MT-targeting agents bind to individual tubulin dimers and block tubulin polymerization (MT-destabilizing agents); others bind on the luminal side of the MT lattice and inhibit MTs’ ability to depolymerize (MT-stabilizing agents). Mutations in the MT-interacting/HIF1α-interacting domain are more often linked to changes in MT polymerization and depolymerization rates (such as the *VHL* type 2A mutant pVHL30(Y98H)), and therefore, such disease could be treated with MT-destabilizing agents. *VHL* mutations in the Elongin C-interacting domain are more often linked to changes in the catastrophe and rescue frequencies (such as the *VHL* type 2B mutation pVHL30(R161P)), and therefore, such disease could be treated with MT-stabilizing agents. In *VHL* ^*−/−*^ cells, targeting the S-phase kinase associated protein 2 (Skp2) attenuates MT polymerization rates and leads to mitotic arrest and cell death (Pu et al., 2025), which indicates a clinical utility of Skp2 inhibitors, such as dioscin (Jiang et al., 2020).

#### Targeting cholesterol uptake

PC extracellular vesicles mediate intercellular communication with the bone marrow and promote metastasis in a cholesterol-dependent manner (Henrich et al., 2020). The accumulation of cholesterol may protect tumors from elevated lipid peroxidation in their microenvironment and ferroptosis (Zhao et al., 2023). Exosome uptake depends on cholesterol-rich membrane microdomains called lipid rafts, and can be blocked by depletion of plasma membrane cholesterol. High-density lipoproteins (HDL) are dynamic natural nanostructures that function to sequester, transport, and deliver cholesterol. Scavenger receptor type B-1 (SR-B1), found in lipid rafts, is a receptor for cholesterol-rich HDL. We demonstrated that a synthetic nanoparticle (NP) mimic of HDL removes cholesterol through SR-B1 and also inhibits cellular exosome uptake (Plebanek et al., 2015). Morphology and motion analysis suggested that HDL-like NPs bind SR-B1 in lipid rafts leading to clustering and arrested movement of SR-B1. In some malignant cells, *de novo* cholesterol synthesis genes are transcriptionally silent or mutated, meaning that cholesterol uptake from lipoproteins is required for survival. HIF1α drives resistance to ferroptosis in solid tumors by promoting lactate production (Yang et al., 2023) and a decrease in HIF1α induces ferroptosis in clear cell renal carcinoma (Green et al., 2022). In cholesterol-addicted lymphoma cells, targeted reduction of cholesterol uptake blocks turnover of oxidized lipids in the cell membrane and selectively causes tumor ferroptosis (Rink et al., 2021). It is conceivable that HDL NPs may have translational relevance in a range of cholesterol-addicted cancers that are sensitive to cell death by ferroptosis following GPX4 depletion.

#### Neutralizing oncogenic activity

RAS-driven cancers have proven very difficult to treat (Hong et al., 2020; Stephen et al., 2014; Zhang et al., 2018), and the most common *KRas* p. G12C mutation can be targeted via proteins fused to the pVHL E3 ligase (Bery et al., 2020; Bond et al., 2020). Decades ago, Rap1, a small GTPase very similar to RAS, was observed to suppress oncogenic RAS phenotype and the proposed reason had been competition between RAS and Rap1 for a common, unidentified target (Kitayama et al., 1989). Further, in the context of metastasis, Rap1 and RhoA GTPase signaling reduces the size and inhibits the sliding of FAs in PC cells (Spanjaard et al., 2015). *Rap1* is often mutated in PC (*Rap1GAP* mutations) and can promote Rho signaling to recruit myosin to the cell edge (Rothenberg et al., 2023). Unfortunately, the Rap1 suppression action on oncogenic RAS via inhibitors does not appear likely (Nussinov et al., 2020) and indirect methods for RAS inhibition might be worth testing. RAS proteins are binary switches, cycling between (ON) and (OFF) states during signal transduction. These switches are normally tightly controlled, but mutations in the *Ras* genes or their regulators render RAS proteins persistently active (Simanshu et al., 2017). RAS is activated by GTP loading and deactivated upon GTP hydrolysis to GDP, that is, (ON) and (OFF) states, respectively. Therefore, healthy MTs compete with oncogenic RAS for GTP, and we could indirectly limit RAS activity by restoring the normal functioning of the MT cytoskeleton (Basseville et al., 2015), which is dysregulated in cancer (Ertych et al., 2014; Galletti et al., 2014; Gonçalves et al., 2001; Harkcom et al., 2014; Thoma et al., 2010). We showed (Harkcom et al., 2014) that NAD^+^ can restore MT dynamics to normal ranges in a BC cell line model and protect MTs in degenerating rat neurons. We will further test the efficacy of different organoid media components, such as nicotinamide and ROCK inhibitors (Nissley et al., 2024), in the context of combination therapies neutralizing the disrupting impact of oncogenic activity. The affinity of RAS for GTP is within the pM range (Bos, 2018) and the strategy of limiting the available pool of GTP molecules is applicable only to quiescent cells that are starved (Matov, 2024e) and terminally differentiated (Af Hällström et al., 2014).

To this end, drug combinations that maintain MT polymerization (like the taxanes and epothilones) and inhibit switches to depolymerization or pausing could be utilized together with compounds that induce an activity of MT severing enzymes (the ATPases katanin, spastin, and fidgetin) (Kuo and Howard, 2021). MT severing proteins catalyze tubulin exchange along the MT lattice and, thus, patch up the new-formed GDP-bound MT ends with GTP-bound tubulin (Vemu et al., 2018). Phosphorylation of Op18 (Cassimeris, 2002; Tournebize et al., 1997) and/or the inhibition of KIF2C (Manning et al., 2007) would reduce the MT frequency of catastrophe as well as increase the sensitivity to taxane treatment (Mistry and Atweh, 2006; Pao et al., 2025). Treatment with the histone deacetylase TSA would increase the MT polymerizing plus pausing times (that is, the overall time MTs spend before switching to depolymerization) (Matov, 2025a). This way, by considering the combined effects on MT dynamics induced, we could identify a drug combination that maximizes the availability of polymerizing MT ends that recruit GTP molecules and minimize their availability to the RAS GTPases. Quantitative live-cell image analysis of MT dynamics will, thus, facilitate the measurements of the cellular effects of BBO-10203 (Simanshu et al., 2025; Sinkevicius et al., 2025) and other novel therapies targeting both the active (ON) and inactive (OFF) conformations of KRAS, such as BBO-8520 (monotherapy or in combination with pembrolizumab) (Maciag et al., 2025; Solomon et al., 2025) and BBO-11818 (Espinosa et al., 2025), in the context of describing and anticipating the emerging mechanisms of acquired drug resistance in patient-derived cells.

#### Rho GTPase inhibitors

**FIGURE 5:**
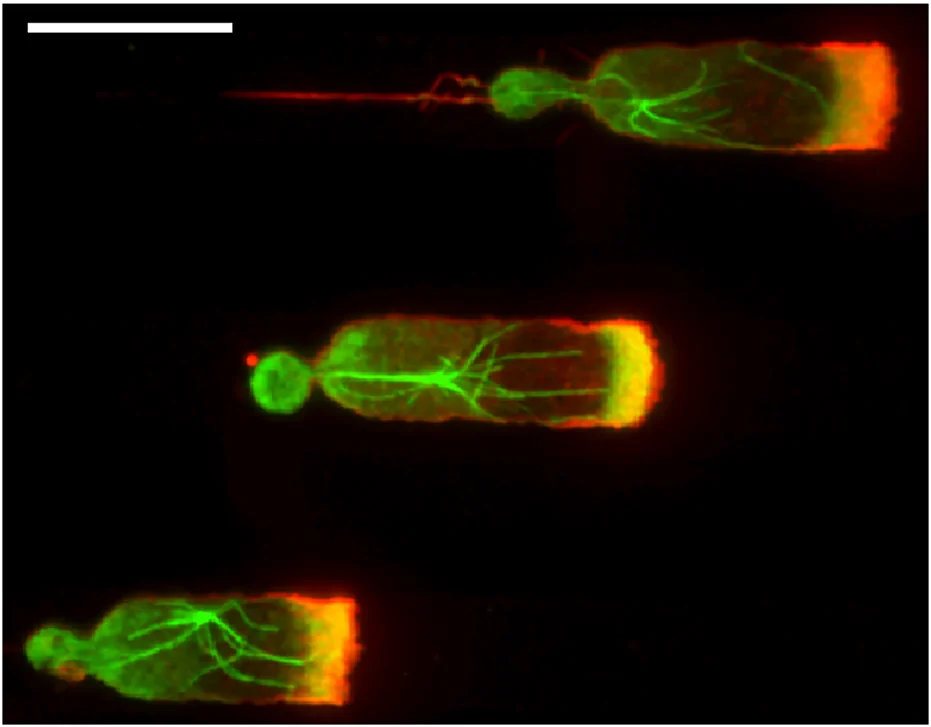
Crawling neutrophils. MTs are pseudo-colored in green, actin in red. Scale bar equals 10 µm.

In epithelial tumor cells, the concentration of GTP is between 0.1 and 1 mM (Kopra et al., 2023), while in neutrophils (Figure 5) it is about 130 pM (Stoehr and Smolen, 1990). There is roughly the same proportion of *KRas* mutations in colon cancer as in benign adenomas (Bos, 2018), which indicates that while a mutation in *Ras* is a prerequisite for malignant transformation, it is not sufficient. *Ras* mutations have been detected in tumors in remission and have been absent in recurrent disease, suggesting that RAS contributes at different stages (Bos, 1989). This indicates that genetic sequencing alone cannot determine the best regimen and that dynamic cellular readouts can contribute to elucidating neoplastic processes. In pancreatic cancer, a *KRas* mutation is present in 95% of the tumors (Simanshu et al., 2017) and tubulin inhibitors are not approved for treatment, yet there are cases in which MT targeting has been very successful (personal communications). We measured a decrease in the apoptotic peaks of DNA fragments from mono- and di-nucleosome-protected DNA in blood samples from pancreatic cancer and CRC patients (Matov, 2024a), indicative of a shift to another cell death pathway – perhaps linked to GPX4 and ferroptosis. During solid tumor pathogenesis, ferroptosis of hematopoietic cells occurs in parallel to tumorigenesis of epithelial cells (Dixon and Stockwell, 2014; Toyokuni et al., 2020). In CRC, GPX3 is underexpressed as early as adenoma stage (Barrett et al., 2013); GPX3 is involved in the detoxification of soluble reactive oxygen species (Dixon and Stockwell, 2014) and has an immunomodulatory role limiting the development of inflammatory tumorigenesis. It is produced mainly in the kidneys and secreted into the blood (Avissar et al., 1994), where it inactivates oxidative damage to maintain genomic integrity by reduction of DNA-damaging agents. In PC, we have shown that mesenchymal tumor cells with acquired resistance after treatment with tubulin inhibitors might be susceptible to ferroptotic drugs (Matov, 2024b). Unfortunately, in the treatment of all epithelial tumors (but receptor-positive BC), clinically the MT cytoskeleton is targeted only after all other options have been exhausted and the immune system is damaged, which prevents us from evaluating the effects of adjuvant and neoadjuvant therapy.

There are long-term side effects associated with the prolonged use of the traditional tubulin inhibitors, which are likely to induce blood cancers and/or neurodegeneration. However, novel tubulin inhibitors, used early in disease progression for a very limited time period, can be very impactful and it is likely that the treatment of pancreatic, prostate, lung, and renal tumors has been hampered by the lack of the right disease models. It is conceivable that pancreatic and colorectal tumors, for instance, develop as a consequence of ferroptotic damage in neutrophils, which are selectively damaged by oncogenic KRAS. Treatment with ROCK inhibitors, such as Y-27632 (Matov, 2024e), has been shown to sensitize pancreatic tumors to doxorubicin (Barcelo et al., 2023). It could be also tested whether inhibitors of Rac1 (Wittmann et al., 2003; Wittmann et al., 2004) and other members of the Rho GTPase family, like Cdc42 and RhoA (Watanabe et al., 2005; Wittmann and Waterman-Storer, 2001; Wittmann and Waterman-Storer, 2004), would sensitize tumors to tubulin inhibitors and whether the mechanism of action is linked to tumor neutrophils.

#### ROCK inhibitors susceptibility

ROCK regulates myosin light chain phosphorylation – and as a result, actin activity and cellular contractility (Matov, 2024f; Matov, 2024j; Matov, 2025b; Narumiya et al., 2000). In PC, we showed that its inhibition reduces the ability of prostate epithelial cells to divide without affecting the primary prostate tumor cells (Matov, 2024e), which are terminally differentiated (Af Hällström et al., 2014). Inhibition of the RhoA-ROCK-myosin pathway promotes acquired drug resistance and the resulting attenuation of p53-induced apoptosis mediates the survival of drug-tolerant persister cells (Wang V. E. et al., 2017). MT dynamics also regulate the activity of p53 by virtue of mediating its translocation to the nucleus by cytoplasmic dynein (Giannakakou et al., 2000b). Modulation of MT dynamics could increase the level of nuclear accumulation of p53 (Giannakakou et al., 2002), which, in turn, could counter the effects of Rho inhibition and may result in increased cell death (Wang V. E. et al., 2017).

Further, the clinical utilization of ROCK inhibitors (Wolff et al., 2024) can benefit from an evaluation of the precise effects of such compounds on the cytoskeleton (Guan et al., 2023). Our analysis indicates that treatment with blebbistatin (Straight et al., 2003), which replicates the effects of a ROCK inhibitor, leads to the formation of a smaller retrograde F-actin network in migrating cells and attenuates the anterograde F-actin flow (Matov, 2024j; Matov, 2025b). The upregulation of Rho/ROCK in cell lines predisposes tumor cells to necroptosis (Zhu et al., 2021), which suggests targeting approaches involving the modulation of MT and FA dynamics. Quantitative live-cell image analysis of MT, actin, and FA dynamics will, therefore, elucidate mechanisms of drug resistance to ROCK inhibitors in the context of describing and anticipating the emerging mechanisms of acquired drug resistance in patient-derived cells and inform therapy.

#### Susceptibility to microtubule-targeting agents

During patient treatment, different MT-associated proteins are activated depending on the drug dose administered and consequently, different resistance mechanisms may be triggered (Matov, 2024e; Matov, 2024h). The effects of tubulin inhibitors change nonlinearly with a dose increase. We have demonstrated that while a high dose of nocodazole depolymerizes MTs, a low dose, which could lead to no side effects, increases MT polymerization rates (Thoma et al., 2010). Further, our new measurements demonstrate that while a high dose of paclitaxel stabilizes MTs, a low dose similarly increases MT polymerization rates (Matov, 2024h). High tubulin inhibitor doses, which disrupt the MT network, would result in decreased nuclear accumulation of p53 (Lloyd, 2002) and persistent drug resistance. There are instances in which tubulin inhibitors could help the tumor cells progress during cell division by dampening MT dynamics and, thus, reducing chromosomal instability (Ertych et al., 2014). Our new measurements indicate the treatment with paclitaxel with up to 33 nM increases the MT catastrophe frequencies, but a treatment with 100 nM reduces the frequencies of MT catastrophe (Matov, 2025a). We have previously demonstrated that while nocodazole or TSA treatment alone do not affect the rates of MT depolymerization, the combined treatment of nocodazole and TSA attenuates the MT depolymerization rates (Matov et al., 2010). Our new measurements indicate that nocodazole treatment increases the times of MT pausing and the catastrophe frequencies, while the histone deacetylase inhibitor TSA decreases the frequencies of MT catastrophe (Matov, 2025a). Our computational results demonstrate multiple non-canonical functions of MT-targeting drugs and indicate that solely the rates of MT polymerization are very often insufficient to dissect their mechanism of action.

#### PARP inhibitors susceptibility

In CRC, *BRAF(V600E)* mutant tumors can successfully be targeted by combination therapy of B-RAF and EGFR inhibitors (Prahallad et al., 2012). However, 20% of colon tumors exhibit a *BRAF*-like genetic signature without a *BRAF(V600E)* mutation (Tian et al., 2013). For these tumors, MT-destabilizing agents appear to have a clinical utility (Linn et al., 1994; Vecchione et al., 2016). Analysis of BC cell lines demonstrated that receptor-positive cells are resistant to vinca alkaloids (Matov, 2024h), but respond to MT-stabilizing agents. Further, receptor TNBC is universally resistant, but could be sensitized to MT-stabilizing agents by targeting the particular aspects of MT dynamics and spindle regulation that render the cancer cell aggressive (Matov et al., 2015). Selective inhibition of KIF2C/MCAK in tumor cells, which regulates MT depolymerization rates (Matov, 2024h), can inhibit DNA repair (Zhu et al., 2020) and that could sensitize BRCA1/2-deficient tumors (Matov, 2024e) to treatment with PARP inhibitors. We derived a PC organoid from micro-metastasis at a retroperitoneal lymph node (Supplementary Figure S2) for which whole genome sequencing showed homozygous mutations in *PTEN* (exon2, c. G194>C, p. C65S) and *BRCA2* (exon14, c. T7397>C, p. V2466A) as well as heterozygous *KIF4B* (exon1, c. G1739>T, p. R580L) and *Rap1GAP* (exon8, c. G511>A, p. A171T) mutations. KIF4B is a protein that is important in the spindle midzone (Bieling et al., 2010); it suppresses MTs’ dynamic instability by dampening both polymerization and depolymerization rates at their plus ends (Yildiz, 2025), and mutations could lead to the formation of larger spindles. In this context, such tumors could be treated with a combination of olaparib and a vinca alkaloid.

#### PLK1 inhibitors susceptibility

**FIGURE 6:**
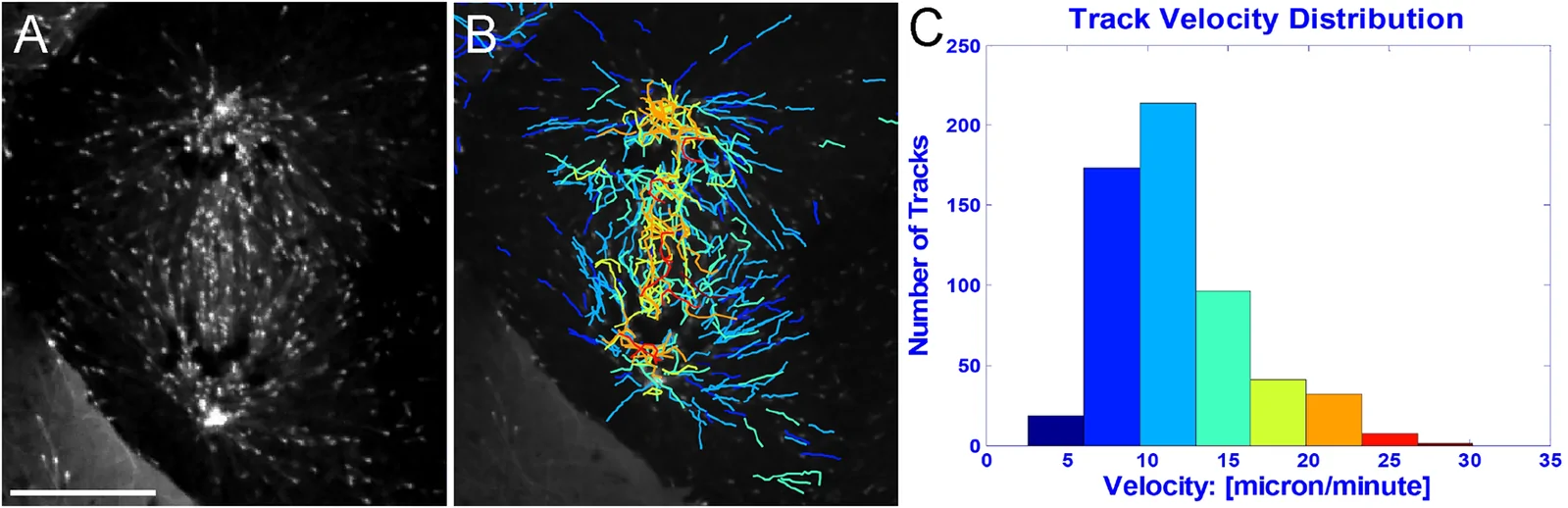
Mitotic spindle and MT dynamics in a dividing epithelial cell during early anaphase. **(A)** MT ends are labeled with EB1ΔC-2xEGFP and imaged for 2 minutes with an acquisition rate of an image every 2 seconds (Brennan et al., 2008), and **(B)** computationally tracked (Yang et al., 2005). The color-coding represents EB1 speeds and colder colors correspond to lower speeds, and warmer colors correspond to faster speeds. Scale bar equals 20 µm. **(C)** Histogram of EB1 speed probability density function of the trajectories shown on **(B)**. The average speed is 11.9 µm/min ± 4.3. The maximal EB1 comet speed is about 30 µm/min. The speed distribution is unimodal and positively skewed.

During cell division, chromosome segregation is achieved through the integration of two processes – anaphase A, during which chromosomes are drawn to the spindle poles, and anaphase B, during which the entire spindle apparatus elongates. Inhibition of polo-like kinase 1 (PLK1) with a small molecule added during the onset of anaphase prevents the anaphase B spindle elongation while not influencing anaphase A (Brennan et al., 2007). Existing models for anaphase B emphasize the role of MT-sliding kinesin motors acting at the spindle midzone to drive the poles further apart. We tested the hypothesis that PLK1 was regulating the activity of a kinesin by depleting an array of spindle-associated motors by RNA-interference. The removal of individual motors (for instance, KIF4A) or combinations thereof failed to reproduce the PLK1 inhibitor-induced short spindle effect (Brennan et al., 2007). This is consistent with studies of spindle length regulation that have shown little dependence on spindle motors, but sensitivity to factors influencing MT dynamics (Milunović-Jevtić et al., 2014; Milunović-Jevtić et al., 2013) and reeling-in forces generated by spindle pole-associated MT depolymerases that contribute to the mechanics of flux (Cameron et al., 2006; Gaetz and Kapoor, 2004; Rogers et al., 2005). Therefore, we tested whether PLK1 inhibition may block anaphase B by altering MT polymerization dynamics within the spindle. Both kymograph and computational EB1 detection (Vid. 2) and tracking (Figure 6) show a significant decrease in MT growth rates in the presence of PLK1 inhibitor, suggesting a role for PLK1 in regulating MT dynamics. Approximately 30% of TNBCs exhibit functional loss of the retinoblastoma (RB) tumor suppressor (Witkiewicz and Knudsen, 2014), which makes such tumors uniquely vulnerable and susceptible to drugs that target chromosome segregation.

Analysis of clinical samples from RB-deficient BC demonstrate that these tumor cells express high levels of DNA replication and G2/M factors, and overexpress the specific targets of PLK1 inhibition (Witkiewicz et al., 2018). In dividing cells, PLK1 inhibition has no effect on spindle midzone MT depolymerization kinetics during anaphase (Brennan et al., 2008). However, it reduces the rates of MT polymerization during early anaphase (Figure 6), which may result in a reduction in the average MT length, causing the formation of shorter spindles and leading to more aggressive disease (Matov, 2024e). This effect on the regulation of mitosis can be reversed by tubulin inhibitors (Brennan et al., 2008) that stabilize MTs – in this scenario, at their minus ends.

### Patient-derived cultures

#### Cancer subtypes

In PC, the known molecular subtypes are TMPRSS2–ERG fusion, SPOP mutation, SPINK1 overexpression, CHD1 loss and mutations in FOXA1, PIK3R1 and in DNA repair and chromatin modifier pathways, loss of PTEN, p53 and RB tumor suppressor pathway functions. Organoids derived from each of the seven arms (subtypes) can be analyzed to study drug efficacy and accumulate a large population of novel signatures from a small number of patients, for instance three per arm, for statistical analysis. From the list of most common mutations, it becomes clear that for some tumors MT regulation would be very affected. There are cellular mechanisms that can lead to a complete response in each tumor subtype and the regimen that triggers it can be identified. We have worked on the establishment of patient-derived organoids and derived cultures from intraductal primary tumors (Supplementary Figure S3) and other adenocarcinoma tissues, which, unfortunately, when cultured are outgrown by the normal epithelial cells. Prostate epithelium contains progenitor cells, which differentiate in a reversible process and early PC (Gleason score 3) is a histological disease without any common molecular driver (Network, 2015). Organoid cultures from such tumors, while initially displaying tumor histology (Supplementary Figure S4), revert to normal physiology within months. We also derived organoid cultures from distant metastatic tumor liver biopsy (Supplementary Figure S5) and micro-metastasis at the retroperitoneal lymph nodes (Supplementary Figure S2). MT end labeling allowed to visualize (Supplementary Figure S6) and analyze (Matov, 2024h) polymerization dynamics in metastatic cells. Our objective has been to conduct measurements over an extended period of time to obtain statistically representative results and, in such a scenario, to minimize photo damage and image cells under x-y-z isotropic resolution (of 1 µm), we have performed initial imaging using lattice light-sheet microscopy (Supplementary Figure S7 and Video S3).

#### Anticipating resistance

If we assume that exceptional responders can be identified for each molecular cancer subtype, we could next attempt to sensitize each tumor to treatment with its corresponding regimen by modulating the cytoskeleton. For instance, vinblastine treatment induced a complete and long-lasting remission in a late stage, drug resistant metastatic colorectal tumor after only a few doses (Linn et al., 1994). About two decades later, genetic sequencing showed the tumor to have a *BRAF*-like signature for which a vulnerability was identified, making RanBP2/Nup358 essential for survival (Vecchione et al., 2016) in cells with reduced MT nucleation at the kinetochores. The response of such tumors might not be uniform and the chances of inducing remission could be improved by a particular sensitizing step, for example by targeting TPX2 (Rohrberg et al., 2020). *BRAF*-like tumors carrying a *KRas* mutation, but not a *BRAF(V600E)* mutation are susceptible to vinorelbine (Vecchione et al., 2016), indicating an interplay between oncogenic *Ras* and MT dynamics. Mutation in *BRAF(V600E)* could be a target in TNBC (Wang L. et al., 2022; Zheng et al., 2016) and our analysis showed no effects on abnormal oscillations of the mitotic spindle after treatment with a B-RAF inhibitor alone or in combination with an EGFR inhibitor or *Myc*-knockdown (Matov, 2024h; Matov et al., 2015). This suggests the need to combine a B-RAF inhibitor with compounds affecting MT polymerization dynamics, that is, targeting the MT polymerase CKAP5/XMAP215 (Conte et al., 2003), CLIP170 (Hu et al., 2022) or MAP4, which are upregulated in TNBC (Wang W. et al., 2022).

Based on our differential expression analysis of the RNA-Seq. data from (Van de Wetering et al., 2015), MAP1B is often upregulated in primary CRC, which suggests that a subset of tumors might be susceptible to MT targeting. MAP1B is overexpressed also in receptor TNBC, but not in other molecular subtypes, and is a prognostic and a therapeutic target (Inoue et al., 2024). We computationally tracked the motion of EB1 comets in primary CRC organoids from patient #9 (Van de Wetering et al., 2015) and identified mitotic spindles exhibiting rapid rotational movements (Matov, 2024h). Further, our differential expression analysis of the RNA-Seq. data from (Van de Wetering et al., 2015) indicated that proteins acting at the MT minus ends and spindle poles (DLGAP5, CENP-E, TPX2, KIF2C, are HMMR) are upregulated and MAP2 is often downregulated. Taken together, our new results suggest resistance to MT-stabilizing agents in CRC and targeting with MT-destabilizing agents (see examples in BC cells in (Matov, 2024h)).

In diffuse gastric cancer cells with different genetic profiles, we identified distinct mechanisms of response to treatment with docetaxel by texture analysis in immunofluorescence image with labeled α-tubulin (Matov, 2024j). For the docetaxel-sensitive HER2-negative SNU1 cells characterized by a *p53* mutation and CDH1 deficiency, the changes include shifts in the values of four cell edge parameters. First, the fraction of above an intensity threshold pixels along the cell edge increased, indicating drug-induced MT bundling and, thus, drug-target engagement. Further, a decrease in the homogeneity in the direction of the cell edge (which identifies patterns containing edges oriented predominantly along a particular direction) indicated a drug-induced rounding of the cells and the MT network. Similarly, a decrease in the ratio of the largest to the next largest value (or bin) in the histogram of the cell edge direction and in the cell edge direction difference (distinguishing MT patterns in which there are parallel edges) indicate a drug-induced disruption of the intricate MT network. Five parameters, four of which distinct from the changes induced in SNU1 cells, were affected in docetaxel-sensitive AZ521 cells, derived from the HuTu80 cell line, which is characterized by a *p53* mutation. We measured changes in four texture parameters related to the convex closure of the MT cytoskeleton after treatment with docetaxel. The length of the morphological skeleton of the MT network increased after drug treatment – indicating MT hyperstabilization – whereas the ratio of the morphological skeleton length to the area of the convex closure of the skeleton of the MT network decreased, indicating spreading of the cells. Further, the fraction of the MT network convex closure pixels above an intensity threshold as well as the roundness of the convex closure of the MT network increased – and the increase in these two parameters indicates MT bundling. The docetaxel-sensitive TMK1 cells harbor *p53* mutations and *p15* (*INK4b* or *MTS2*) rearrangement, and we measured statistically significant shifts in the values of 11 parameters, seven of which distinct from those changed in SNU1 and AZ521 cells. Five of the seven distinct parameters were related to drug-induced changes in the distribution of MTs within the cells. We measured an increase in the numbers of bright MT objects (MT areas), a decrease in the values for the Euler number (which distinguishes between reticular and mesh-like patterns), a decrease in the numbers of above-threshold pixels per MT area, an increase in the variance of the number of bright pixels per MT area, and a decrease of the variance of the MT areas distance to the cell center of fluorescence – all five changes indicate MT bundling after docetaxel treatment. The full list of parameters and their changes, or lack thereof, after docetaxel treatment are available in Matov (2024i).

It is difficult to translate these studies to the clinic and in the cases when drugs are approved, resistance is unpredictable. *Ex vivo* analysis of patient-derived cells after fluorescent labeling of tubulin, actin, and other cellular proteins could indicate which treatment options are more likely to trigger resistance mechanisms. Patient cultures rapidly diverge from the original tumor when grown in the laboratory, however they are likely to represent relatively well the original tumor dynamics and kinetics during the first passages, that is, within days. At first, we imaged organoids 80 h after lentiviral transduction (Supplementary Video S4), that is, about 4 days after surgery (Supplementary Video S5). We optimized the lentiviral infection protocols and were able to efficiently label organoid cells after using 40-fold less viral particles and measured fluorescent MTs overnight after transduction, that is, about 14 h after patient tissue resection. The analysis of patient cells is a viable option to answer outstanding questions in the biology of tumors, for instance during immunotherapy, which can only be addressed by cell biological approaches (Mitchison, 2021). Mechanism of acquired resistance to drugs that modulate the function of p53, such as milademetan (Arnhold et al., 2018; DiNardo et al., 2016), can be elucidated with our computational approach.

### Microtubule regulation during neurodegeneration

Beyond cancer, in Alzheimer’s disease, the overexpression of galectin-3 induces the formation of tau aggregates (Boza-Serrano et al., 2022) called neurofibrillary tangles, which leads to MT destabilization and depolymerization, resulting in neuronal death. Targeting galectin-3 (TrueBinding, 2023) can reverse these processes and improve memory in Alzheimer’s disease patients. To perform patient stratification and anticipate drug resistance, an *ex vivo* analysis of MT dynamics before and after treatment with TB006 can be performed in induced pluripotent stem cell (iPSC)-derived patient axons (Matov, 2024j). In Alzheimer’s disease, targeting the MT-associated protein tau is impactful and there are pronounced changes in mitochondrion morphology (Matov, 2024j), leading to the accumulation of reactive oxygen species (Ježek et al., 2018). Drugs that modulate mitochondrion dynamics have been experimentally tested in iPSC-derived neurons of patients with ALS and Alzheimer’s disease (Saporta et al., 2011). Drug treatments of iPSC-derived patient neurons induced further changes in the morphology and dynamics of mitochondria along MTs (Matov, 2024j). Mitochondrion dynamics can emerge as a novel target for developing next-generation immunotherapy agents for cancer (Baldwin and Gattinoni, 2022; Saha et al., 2022). Novel compounds that reduce the time mitochondria pause have applications in the treatment of neurodegenerative diseases and quantitative live-cell image analysis of mitochondria in patient-derived neuronal cells can be utilized for functional testing of putative compounds to improve the process of drug discovery.

Our aim is to contribute to the field of oncology by identifying proteins whose targeting can sensitize patient tumors and enhance the effects of immunotherapy (Luo et al., 2021), such as the MT minus end-directed motor (cytoplasmic) dynein (Gros et al., 2021) and KIF2C/MCAK, which exerts an oncogenic role in non-small cell lung cancer and is negatively regulated by microRNA-325 (Gan et al., 2019). However, there is crosstalk between MT regulation in cancer and neurodegeneration, and Parkin, for instance, is a MT regulator (Cappelletti et al., 2015), and tubulin inhibitors affect neuronal cells as well. We analyzed lysosomal trafficking in mouse astrocytes in Parkinson’s disease to test the efficacy of putative compounds (Matov, 2024j). Our analysis demonstrated that the *LRRK2-G2019S* mutations lead to lysosome motion away from the nucleus and faster than normal lysosome speeds of over 12 μm/min with an increased overall displacement of about 71 µm (Matov, 2024j). These new results suggest that there is dysregulation of MT-associated proteins and offer a quantitative strategy to inform therapy.

## Discussion

We demonstrate that tubulin inhibitors induce a variety of effects in cells and that their mechanism of action depends on the particularities of the regulation in tumor cells. With the computational analysis of different parameters describing MT homeostasis, it becomes clear that the canonical effects of tubulin inhibitors on the MT cytoskeleton and cellular regulation are representative for only the particular cases and types of cells they have been established in. Successful clinical treatment with tubulin inhibitors requires quantitative live-cell image analysis of patient-derived cells before and after *ex vivo* treatments. For precision medicine, sequencing of the patient tumor alone – while indicative of the particularities of the disease – cannot determine which molecular mechanisms prevail after treatment with a specific drug combination. Computational analysis of the MT and actin cytoskeleton using the quantitative assay we propose will facilitate the evaluation of an optimal modulation of the cytoskeleton in patient tumors. We relate drug resistance to the ability of a drug regimen to modulate cytoskeletal dynamics in the particular parameters affected by a tumor (Matov, 2025a) and the analysis we perform can elucidate strategies for overcoming drug resistance in tumor types currently considered resistant to therapy. In addition, our approach to drug resistance could be applied during drug development to identify novel compounds with specific functions.

Further, our approach will facilitate the creation of a medical digital twin (Björnsson et al., 2019; Casola, 2023; Eddy and Schlessinger, 2003; Laubenbacher et al., 2024; Sadée et al., 2025). A digital twin can be broken down into the following five essential components: physical object (patient), data connection, model (patient-in-silico), interface, and twin synchronization. The understanding that the availability of the full information allows us to make correct, autonomous decisions, in particular in regard to medical decisions, has been formalized since the 18th century (Kant, 1781; Kant, 1797). According to a report from the FDA, medication is deemed ineffective for 38%-75% of patients with common diseases (US Food Drug Administration, 2013), reflecting that the healthcare system has been relying on a small number of biomarkers of limited sensitivity or specificity. A medical digital twin focuses on the patient’s health, as described by multimodal data – including, but not limited to, laboratory tests, imaging, sequencing, and the electronic health records (EHRs).

In cases with localized medical issues, the patient becomes the specific organ until a broader context is required. Patient-derived organoids can serve as organ and disease models. Novel technologies that merge AI approaches and mechanistic models offer enormous promise in generating a high-fidelity patient-in-silico model. The interface allows the clinical team to establish the effect of different therapies, query the patient-in-silico, and subsequently integrate its findings into the EHR. The findings are evaluated by uncertainty quantification (Chan and Elsheikh, 2018; Elsheikh et al., 2012; Sumner et al., 2012), which tracks the accumulation of different errors from data acquisition to modelling. Synchronization, based on liquid biopsy and body fluids testing, takes place when recording a substantial deviation from the baseline or measuring the effects of patient treatment. For treatments of diseases such as cancer, twin synchronization might involve updating the medical digital twin after each chemotherapy cycle.

Novel drugs can be evaluated with our computational approach during drug development in combination with a benchmark dose gene expression analysis (EFSA Scientific Committee et al., 2022). The use of gene expression signatures to classify compounds, identify efficacy or toxicity, and differentiate close analogs depends on the sensitivity of the method to identify modulated genes. Ligation-based targeted whole transcriptome expression profiling assays are able to determine whether previously unreported compound-responsive genes could be identified and incorporated into a compound-specific signature – for example, related to the activity of the histone deacetylase inhibitor TSA (Yeakley et al., 2017), which affects multiple parameters of MT dynamics (Matov, 2025a). Our quantitative computer vision measurements in living cells can be considered together with transcriptomics datasets and concentration-response modeling (Harrill et al., 2021). This approach can evaluate the transcriptional biological pathway altering concentrations (Judson et al., 2011) and the toxicokinetics of novel compounds. Dose-response curves indicate when a drug concentration induces drug efficacy or triggers toxicity (Ramaiahgari et al., 2019) based on gene expression and the metabolism induced. The analysis will elucidate the specific changes induced by the drug doses applied to patient cells *ex vivo* and relate the cytoskeletal dynamics metrics we compute to pharmacogenomics during drug discovery. This way, we can identify dependencies between each parameter of MT and F-actin dynamics, and the metabolic changes in patient cells. It will allow us to dissect the differences in drug action between drugs that bind to the same domain, for examples taxanes or vinca alkaloids.

Publications from the Clevers lab have shown that a single adult tissue stem cell, when cultured using a comprehensive list of key medium components that mimic the growth environment *in vivo*, can form organoids (Sato and Clevers, 2013; Sato et al., 2009). Since then, work in Utrecht and by others has shown that, under the right conditions, organoids derived from colon (Van de Wetering et al., 2015), prostate (Gao et al., 2014) and other patient tumor tissues (Lee et al., 2018; Sachs et al., 2018; Sachs et al., 2019; Tiriac et al., 2018; Vlachogiannis et al., 2018) can be propagated in 3D tissue culture. In drug-naïve PC, it seems that the lack of an appropriate tumor-associated stromal culture medium component (Olumi et al., 1999) presents a challenge. In our experience, paracrine signaling is key to organoid growth. Cultures that lose the cancer-associated fibroblasts, seize growth and become quiescent. That result suggests the need to optimize the culture medium and Matrigel formulation (Matov, 2025a). The most common metastatic site in PC is the bone (84%) and the liver is most commonly the secondary site (39%) (Gandaglia et al., 2014). Neither organoid culture nor murine models provide the required environment for patient cells from bone metastasis. To provide a more suitable environment, we propose to culture cells derived from vertebral (L2 vertebral body) metastases on hFOB 1.19 cells, which mimic the human bone, in a specialized microfluidics device (Matov, 2024b; Zhang et al., 2015). The bone marrow culture medium consists of RPMI with L-glutamine, patient plasma, CaCl2, sodium succinate, hydrocortisone, and heparin (Zhang et al., 2015), and one should allow for 48 h of metabolic recovery after the biopsy before an *ex vivo* drug treatment (since, for instance, freshly resected and sheared tissue requires 2-12 h of recovery in PC media to reach a peak in metabolic activity (Keshari et al., 2013; Maund et al., 2014)). After plating patient cells in such microfluidics, about 70% of them survive (Zhang et al., 2014). To increase the rates of patient cell survival after plating further, additional media components could be added both in the context of mimicking the bone marrow (Aleman et al., 2019) and improving fibroblast activation (Tran et al., 2013; Tran et al., 2012). The utilization of a culture system that closely recapitulates the tumor microenvironment in the human bones will allow us to improve and validate our *ex vivo* analysis results.

We propose the utilization of organoid analytics to generate embeddings (Vaswani et al., 2017) for a generative transformer network in which the tokens are the texture metrics (Matov, 2024i) and growth kinetics (Matov, 2024j) of the organoids. The embeddings will also include the parameters of MT dynamics before and after treatment with panels of drugs. We will retrain a transformer network with a new set of tokens – with shape descriptors rather than words and with growth and killing curves (that is, sigmoid curves) rather than sentences of human speech, that is, we will retrain a LLM and a foundation model with organoid morphology data. This will allow us to train the models to classify each organoid as sensitive or resistant and additionally identify subclasses with different mechanisms of drug resistance. Further, after training generative transformer networks with organoid texture as well as live-cell dynamics datasets, we will be able to model disease progression too.

Our interest is the identification of biomarkers of resistance to the available and putative tubulin inhibitors based on the analysis of live patient cells. As all models do not fully recapitulate the tumor environment *in vivo*, one may ask: What can I know? What ought I to do? What may I hope? (Kant, 1797). In this context, to understand the relation between the drug and its target, we worked on the establishment of cancer organoids expressing fluorescently labeled MT plus end tracking proteins, their imaging by spinning disk confocal (Supplementary Figure S6 and Videos S4, S5) and lattice light-sheet (Supplementary Figure S7 and Video S3) microscopy, and the quantification of MT dynamics in such patient-derived cultures. Another alternative to cell lines is the use of patient-derived xenografts (Matov, 2024e), also a challenge in PC, and imaging MT dynamics before and after drug treatment *in vivo* (Janssen et al., 2013).

Our objective is to establish patient MT dynamics as biomarkers of clinical sensitivity to tubulin inhibitors and MT-targeting drugs in solid tumor oncology. Our hypothesis is that there exist tumor-associated alterations of MT dynamics in patient tumors that can be exploited by modulating the corresponding MT-regulating genes and selecting the most effective drug regimen. Our methodology can also be applied to the characterization of the activity of patient immune cells and could contribute to calibrating cell therapy, as it relates to MTs (Matov, 2024g), and to improving its success rate in treating solid tumors.

## Materials and methods

### Sample processing

Small RNA were extracted from de-identified urine samples (50 mL) by Norgen Biotek Corp (El-Mogy et al., 2018).

Retroperitoneal lymph node organoid whole genome sequencing (736 ng DNA) was done at the UCSF Core Facilities.

### Data and image analysis

All programs for hierarchical clustering analysis and graphical and image representation of microRNAs were developed in R: https://github.com/amatov/DiseaseDetectionUrine. The Kullback-Leibler divergence (Kullback and Leibler, 1951) method used for biomarker selection is described and validated in (Han et al., 2016). The computer code is in Python and is available for download at: https://github.com/amatov/FragmentomicsSubclinicalDisease. The Matlab and C/C++ EB1 comet analysis software package is available at: https://github.com/amatov/ClusterTrackTubuline.

To perform automated extraction of line kymograph (Matov, 2025b) figures, Matlab code is available at: https://github.com/amatov/CurvedSpeckleKymograph. See the included image for an example; the program requires a user-defined selection of at least three points (shown as red asterisks on the example image) to create a spline (Hall and Meyer, 1976) based on cubic Hermite interpolation (Hermite, 1864) along the spindle length and generate the kymograph. The user may change the number of pixel slices, that is, the kymograph width; the default number is five. The Matlab code for obtaining the size of spindle poles by solving systems of linear Diophantine equations (Heger, 1858) for the pair-wise line intersections formed by the MT end trajectories head-to-tail is available at: https://github.com/amatov/DyneinFunctionAnalysis.

**FIGURE 7:**
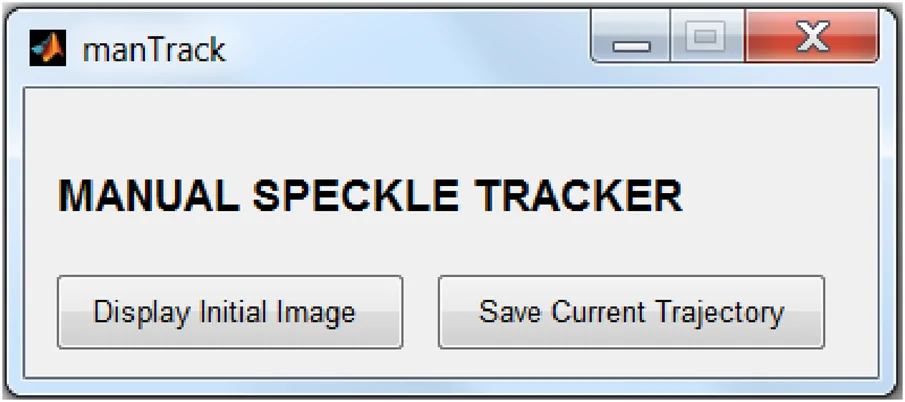
Manual tracking GUI. The software allows users to select an image sequence and annotate speckles as trajectories via a GUI.

The software package for manual tracking and validation (Figure 7) is written in Matlab and available for download at: https://github.com/amatov/FeatureTrackingValidation. To analyze manual tracking bias resulting from the limited sensitivity of the human vision to dim speckles, we present the human operator (or user) with randomly selected speckles for manual tracking. Our software presents the same lists of speckles to the different users (as well as repeatedly asking the same user to track some of the speckles), thus allowing us to estimate the intra-user and inter-user reproducibility. Both metrics indicated a low reproducibility of the results (Danuser, 2009), which came as a surprise, particularly in the case of intra-user variability, since when the program prompted the same user to track the same speckle again – even if that was minutes after the initial tracking selection – the results differed. The inter-user differences were at times drastic, highlighting the high level of difficulty of validating speckle motion data (see also (Vallotton et al., 2003) in this regard).

To detect fluorescent speckles via a scale space-based (Lindeberg, 1993) approach, the images were filtered with a band-pass Gaussian filter (Weierstrass, 1885) with a mask five by five pixels (and pixel size in object space of half the resolution limit or smaller, as per Nyquist (Shannon, 1940)) and ratio of the cut-off filters of 1.1, established heuristically (Jungius, 1622; Matov, 2024c; Matov et al., 2011). The high frequency cut-off (σ_1_) was matched with the limit of the optical transfer function (Fourier, 1822) beyond which the collected signal is white noise and the kernel was computationally fitted to the Bessel function (Bernoulli, 1728) forming the point-spread function of the imaging system. The code for fitting a Gaussian to a Bessel function (as well as simulation of speckle fusing and detection based on the angular resolution limit (Sparrow, 1916) for different signal-to-noise ratios (Arnold and Espenschied, 1923)) is available here: https://github.com/amatov/GeneralizedSparrowCriterion. This way, we matched the optical resolution limit of the collection frequency spectrum based on the dye emission wavelength and the diffraction limit of the microscope objective based on the Rayleigh distance (Rayleigh, 1891). The lower limit (σ_2_) relates to the computational clipping of image background, that is, non-specific signal forming larger shapes in the body of the biological cell and the cut-off was empirically obtained from the available imaging datasets to minimize the false positive feature selections (Matov et al., 2011). To segment image background (and foreground, respectively), we applied mean pixel intensity thresholding (Bernsen, 1986) and Sobel edge detection (Sobel and Feldman, 1968) to the images filtered with a Gaussian filter with a standard deviation σ_1_ (to remove high frequency noise) followed by morphological reconstruction of the segmented areas. We then obtained the intensities of all image background local maxima (I_n_) in the (non-speckle) background/noise image and computed their expected value E [I_n_] and standard deviation Std (I_n_). Next, using the difference of Gaussians (Wilson and Giese, 1977) images for each time frame, after computationally clipping the negative pixel values and a logical multiplication with the image foreground mask, we obtained the image coordinates of all image foreground local maxima (candidate speckles). Assuming a Gaussian distribution of the local maxima values, we performed a statistical significance test (Student’s *t*-test) for each image foreground local maximum (I_max_) against the noise level E [I_n_] + Q Std (I_n_), where the quantile Q was set at 1.96 to approximate a 95% confidence level based on the central limit theorem (Laplace, 1810). The areas of the confirmed speckles in the difference of Gaussian images can be viewed in Supplementary Videos S6, S7 (image registration was done based on cross-correlation analysis during which the translation is corrected via evaluating the covariance matrix by computing the convolution of image pairs (Mitchison et al., 2004)), Supplementary Videos S8, S9. We used the obtained through these steps difference of Gaussians images for recursive accumulation over the full time-lapse image sequences of fading speckle streak patterns in which speckles with long lifetimes are visible as bright streaks (see Supplementary Videos S10, S11). Such speckle streak images allow us to generate grid vectors for an orientation sensitive texture filter indicating the direction of local speckle flow for each image area (Supplementary Figure S8). We performed this computation based on the Jacobian matrix of the speckle streak images. The Matlab code for scale space-based speckle detection and the orientation-sensitive texture filter is available at: https://github.com/amatov/TextureFlowAnalysis.

### Bioinformatics analysis

Sequence reads were trimmed using Trimmomatic 0.36 (Bolger et al., 2014). We used hg38 genome as reference and aligned the reads with BWA-MEM version 0.7.15 (Li and Durbin, 2010). For variant calling, we utilized SAMtool (Li et al., 2009), BCFtools (Li, 2011), Ensembl Variant Effect Predictor v86 (McLaren et al., 2016), VCFtools (Danecek et al., 2011), and ANNOVAR (Wang et al., 2010). To run the analysis, we installed a virtual machine (Oracle VM Virtual Box 5.0.6) and Ubuntu 12.04.5 on a Windows server with Intel Xeon E5-1620 processor with 4 dual cores and a solid-state drive. For the alignment step, we connected to a high performance computing cluster (Mount Moran, 2016).

### Statistical analysis

Statistical tests were performed in Matlab. We performed Kolmogorov-Smirnoff statistics (Kolmogorov, 1933) to compare higher-order moments of the underlying MT parameter per-track distributions and permutation *t*-test to extract information on shifts in median values of polymerization parameters over a population of cells. In brief, for each condition, multiple videos containing one or two cells were compared. Before merging the data for further statistical analysis, we determined the video-to-video variation in the distributions of MT parameters using the Kolmogorov-Smirnov test (Kolmogorov, 1933). Distributions were subsampled to 400 values to avoid hypersensitivity of the Kolmogorov-Smirnov statistics. Videos passing the consistency test were included in the final datasets. Statistical comparison of MT dynamics between different conditions was accomplished in two steps. First, we examined whether the different conditions would profoundly change the distribution of each parameter, which would indicate an entirely different state of MT regulation between conditions. To test this possibility, we aligned the distributions of compared conditions at their respective per-track mean values and then applied the nonparametric Kolmogorov-Smirnov test to determine whether the two distributions are identical. The distributions were randomly subsampled to 400 values. The subsampling was repeated 200 times, and the final p-values were derived by averaging. In a second step, we compared the shifts in parameter mean values between different conditions. To achieve this, we calculated the per-cell means for each parameter. Then we tested normality of the distribution of the means and applied a test to define the significance of parameter changes between conditions (mean ± sem of cell-to-cell variation). This test assumes that the cell-to-cell variation is the single biggest source of variation in the mean value of a condition. None of the parameters of MT dynamics followed a Gaussian distribution. To determine the significance of differences in the mean values between parameters under different experimental conditions, we applied a non-parametric permutation *t*-test with 400 repetitions (Hesterberg et al., 2005), which compares the bootstrapped distributions of a condition 1 versus condition 2. This test does not make any assumption about the characteristic of the tested distribution and, thus, is appropriate for application to distributions that are far from being normally distributed. In brief, for each condition 400 values are bootstrap-sampled from the data. In agreement with the central limit theorem (Laplace, 1810), the two bootstrapped distributions always follow a Gaussian distribution and, thus, could be analyzed for differences using a regular Student’s *t-*test.

### Microscopy imaging

Organoids were imaged using transmitted light microscopy at 4× magnification and phase contrast microscopy at 20× magnification on a Nikon Eclipse Ti system with camera Photometrics CoolSnap HQ2.

MT polymerization dynamics in BC cells were visualized by lentivirus-mediated low level expression of EB1ΔC-2xERFP, a marker of growing MT plus ends that does not interfere with endogenous EB1 binding partners.

We imaged EB1ΔC-2xEGFP-expressing organoids (Koo et al., 2013) by time-lapse spinning disk confocal microscopy using a long working distance 60× magnification water immersion 1.45 NA objective. We acquired images at half a second intervals for a minute to collect datasets without photobleaching (Gierke and Wittmann, 2012).

PC organoids and BC cell line cells embedded in Matrigel were imaged using lattice light-sheet microscopy (Chen et al., 2014) in collaboration with the UCSF Biological Imaging Development Center.

### Cell culture

The BC cell line cells were grown as 3D cultures in Matrigel (Corning) in DMEM (Cellgro, Mediatech) supplemented with 10% FBS (Atlanta Biologicals), incubated at 37 °C in 5% CO_2_.

Primary and retroperitoneal lymph node metastatic prostate tumor and sternum metastatic rectal tumor tissues were dissociated to single cells using modified protocols from the Witte lab (Goldstein et al., 2011). Organoids were seeded as single cells in three 30 µL Matrigel drops in 6-well plates. Organoid medium was prepared according to modified protocols from the Clevers lab (van de Wetering et al., 2015) and the Chen lab (Gao et al., 2014). We expressed fluorescently tagged MT markers in the organoids by using lentivirus-mediated transduction (Koo et al., 2013) and had best results infecting organoids in an exponential growth phase. To optimize imaging conditions, blasticidin selection was tuned such that only 20%-30% of organoid cells are GFP-labeled (Matov, 2025a). This way, we achieved better contrast of imaging EB1 comet and MT dynamics.

To obtain a single cell suspension, tissues were mechanically disrupted and digested with 5 mg/mL collagenase in advanced DMEM/F12 tissue culture medium for several hours (between 2 and 12 h, depending on the biopsy or resection performed). If this step yielded too much contamination with non-epithelial cells, for instance during processing of primary prostate tumors, the protocol incorporated additional washes and red blood cell lysis (Goldstein et al., 2011). Single cells were then counted using a hemocytometer to estimate the number of tumor cells in the sample, and seeded in growth factor-reduced Matrigel drops overlaid with PC medium (Gao et al., 2014). With radical prostatectomy specimens, we had good success with seeding 3,000 cells per 30 μL Matrigel drop, but for metastatic samples organoid seeding could reliably be accomplished with significantly less cells, in the hundreds. To derive organoids from patient CTCs, liquid biopsy samples of 40 mL peripheral blood would be collected, processed, and plated in a Matrigel-collagen-fibronectin matrix to form organoids similarly to the metastatic BC organoids we cultured from mouse CTCs (Matov, 2024e).

To transduce organoids, we modified protocols from the Clevers lab (Koo et al., 2013) to adapt to the specifics of prostate organoid culture (such as the significant differences in proliferation rates in comparison to colon and rectal organoids). We found out empirically that cells in mid-size organoids (60-100 µm in diameter) infect at much better rates than trypsinized single organoid cells, small organoids, or large organoids. These were the steps we followed to express EB1ΔC-2xEGFP in organoids: (1) Add dispase (1 mg/mL) to each well to dissolve the Matrigel at room temperature for 1 h. (2) Spin down (at 1,000 rpm for 4 min) and mix organoids with 10 µl of viral particles (enough for 1 well with three Matrigel drops of 30-40 µL with organoids containing 1-2 million cells), 10 mg/mL Y27632 ROCK inhibitor, and 10 mg/mL polybrene for 30 min. (3) Spin the organoids with viral particles for 1 h at 600 g. (4) Leave the organoids for a 6-h incubation. (5) Spin down and plate in Matrigel. (6) 1 h later, add medium. We used 0.5 mg/mL blasticidin for only one round of medium (3 days), because an increase of the density of labeled cells in the organoids reduced our ability to image MT ends with good contrast (Supplementary Video S12).

Docetaxel, ixabepilone, vinorelbine, and vincristine were obtained from Sigma-Aldrich in powder form. Paclitaxel and eribulin (in intravenous solution) were a gift from Linda Vahdat. To optimize the drug regimens, we performed titration experiments (0.1 nM, 0.2 nM, 0.5 nM, 1 nM, 10 nM, and 100 nM) with the six tubulin inhibitors to obtain the drug concentration at which the cells from the TNBC cell line Hs578-T, the TNBC cell line MDA-MB-231, the ER-positive cell line MCF-7, and HER2-positive cell line SK-BR-3 remain viable for over 168 h after treatment. BI 2536 at a concentration of 250 nM was used for PLK1 inhibition.

## Data Availability

The datasets presented in this study can be found in online repositories. The names of the repository/repositories and accession number(s) can be found below: https://www.ncbi.nlm.nih.gov/, SRX28551163. Lung cancer microRNA datasets can be found here: https://github.com/amatov/DiseaseDetectionUrine/blob/main/LungCancer_miRs.RData.

## Glossary

AMP-PNP: adenylyl imidodiphosphate
ATPase: adenosine triphosphatase
BC: breast cancer
BRCA1/2: breast cancer type 1/2 susceptibility protein
CLASP2: cytoplasmic linker-associated protein, 2
CLIP170: cytoplasmic linker protein, 170
CRC: colorectal cancer
EB1: microtubule end binding protein, 1
EB1ΔC: EB1 construct truncated at amino acid 248 that does not interact with other proteins at the microtubule end
EGFP: enhanced green fluorescent protein (2xEGFP indicates two molecules)
EGFR: epidermal growth factor receptor
EHR: electronic health record
ER: estrogen receptor
FA: focal adhesion
FSM: fluorescent speckle microscopy
F-actin: filamentous actin
GAP: GTPase-activating protein
GDP: guanosine diphosphate
GPX3/4: glutathione peroxidase, 3/4
GSK3β: glycogen synthase kinase 3, beta isoform
GTPase: guanosine triphosphatase
GTP: guanosine triphosphate
GUI: graphical user interface
HDL: high-density lipoproteins
HER2: receptor tyrosine-protein kinase, erbB-2
HIF1α: hypoxia-inducible factor 1, alpha subunit
iPSC: induced pluripotent stem cell
KIF11: kinesin-5 protein (homologs–Eg5 in *Xenopus* and KLP61F in *Drosophila*)
LLM: large language model
MAP1B: microtubule-associated protein, 1B
MAP2: microtubule-associated protein, 2
MAP4: microtubule-associated protein, 4
MT: microtubule
**NAD** ^**+**^: nicotinamide adenine dinucleotide, oxidized
NP: nanoparticle
Nup: nucleoporin
PARP: poly (ADP-ribose) polymerase (ADP–adenosine diphosphate)
PC: prostate cancer
PLK1: polo-like kinase, 1
pVHL: von Hippel-Lindau protein (pVHL30/pVHL19 refer to the long/short isoform)
RanBP2: Ran binding protein, 2
Rap1GAP: Rap1 GTPase-activating protein
RB: retinoblastoma
ROCK: Rho-associated protein kinase
Skp2: S-phase kinase associated protein, 2
SR-B1: scavenger receptor, class B, type 1
TMPRSS2: transmembrane protease, serine 2
TNBC: triple-negative breast cancer
TSA: trichostatin A

## References

[B1] Adams M. Matov A. Yarar D. Gupton S. Danuser G. Waterman-Storer C. M. (2004). Signal analysis of total Internal Reflection Fluorescent Speckle Microscopy (TIR-FSM) and widefield epi-fluorescence FSM of the actin cytoskeleton and focal adhesions in living cells. J. Microsc. 216, 138–152. 10.1111/j.0022-2720.2004.01408.x 15516225

[B2] Af Hällström T. M. Zhao H. Tian J. Rantanen V. Reese S. W. Nolley R. (2014). A tissue graft model of DNA damage response in the normal and malignant human prostate. J. Urology 191, 842–849. 10.1016/j.juro.2013.09.007 24035881 PMC4009951

[B3] Ahuja R. K. M. T. M. Orlin J. B. (1993). Network flows: theory, algorithms and optimization. New Jersey: Prentice-Hall, Inc., 846.

[B4] Airy G. B. (1835). On the diffraction of an object-glass with circular aperture. Trans. Camb. Philosophical Soc. 5, 283–291.

[B5] Albrethsen J. Angeletti R. H. Horwitz S. B. Yang C. P. (2014). Proteomics of cancer cell lines resistant to microtubule-stabilizing agents. Mol. Cancer Ther. 13, 260–269. 10.1158/1535-7163.MCT-13-0471 24252851 PMC3947109

[B6] Aleman J. George S. K. Herberg S. Devarasetty M. Porada C. D. Skardal A. (2019). Deconstructed microfluidic bone marrow On-A-Chip to Study normal and malignant hemopoietic cell-niche interactions. Small 15, e1902971. 10.1002/smll.201902971 31464364 PMC8011350

[B7] Applegate K. T. Besson S. Matov A. Bagonis M. H. Jaqaman K. Danuser G. (2011). plusTipTracker: quantitative image analysis software for the measurement of microtubule dynamics. J. Struct. Biol. 176, 168–184. 10.1016/j.jsb.2011.07.009 21821130 PMC3298692

[B8] Arnhold V. Schmelz K. Proba J. Winkler A. Wünschel J. Toedling J. (2018). Reactivating TP53 signaling by the novel MDM2 inhibitor DS-3032b as a therapeutic option for high-risk neuroblastoma. Oncotarget 9, 2304–2319. 10.18632/oncotarget.23409 29416773 PMC5788641

[B9] Arnold H. D. Espenschied L. (1923). Transatlantic radio telephony, Annual Convention of the A. I. E. E. 2, 116–144.

[B10] Avissar N. Ornt D. B. Yagil Y. Horowitz S. Watkins R. H. Kerl E. A. (1994). Human kidney proximal tubules are the main source of plasma glutathione peroxidase. Am. J. Physiology 266, C367–C375. 10.1152/ajpcell.1994.266.2.C367 8141250

[B11] Bailey C. L. Kelly P. Casey P. J. (2009). Activation of Rap1 promotes prostate cancer metastasis. Cancer Res. 69, 4962–4968. 10.1158/0008-5472.CAN-08-4269 19470770 PMC4195850

[B12] Baldwin J. G. Gattinoni L. (2022). Cancer cells hijack T-cell mitochondria. Nat. Nanotechnol. 17, 3–4. 10.1038/s41565-021-01006-y 34795442

[B13] Bao M. Pan S. Yang W. Chen S. Shan Y. Shi H. (2018). Serum miR-10a-5p and miR-196a-5p as non-invasive biomarkers in non-small cell lung cancer. Int. J. Clin. Exp. Pathology 11, 773–780. 31938164 PMC6958018

[B14] Barcelo J. Samain R. Sanz-Moreno V. (2023). Preclinical to clinical utility of ROCK inhibitors in cancer. Trends Cancer 9, 250–263. 10.1016/j.trecan.2022.12.001 36599733

[B15] Barrett C. W. Ning W. Chen X. Smith J. J. Washington M. K. Hill K. E. (2013). Tumor suppressor function of the plasma glutathione peroxidase gpx3 in colitis-associated carcinoma. Cancer Res. 73, 1245–1255. 10.1158/0008-5472.CAN-12-3150 23221387 PMC3563732

[B16] Barry R. E. Krek W. (2004). The von Hippel–Lindau tumour suppressor: a multi-faceted inhibitor of tumourigenesis. Trends Mol. Med. 10, 466–472. 10.1016/j.molmed.2004.07.008 15350900

[B17] Basseville A. Bates S. Fojo T. (2015). Pancreatic cancer: targeting KRAS and the vitamin D receptor *via* microtubules. Nat. Rev. Clin. Oncol. 12, 442–444. 10.1038/nrclinonc.2015.125 26169922

[B18] Berges R. Baeza-Kallee N. Tabouret E. Chinot O. Petit M. Kruczynski A. (2014). End-binding 1 protein overexpression correlates with glioblastoma progression and sensitizes to Vinca-alkaloids *in vitro* and *in vivo* . Oncotarget 5, 12769–12787. 10.18632/oncotarget.2646 25473893 PMC4350359

[B19] Bernoulli D. (1728). Observationes de seriebus recurrentibus. Novi Comment. Acad. Sci. Imp. Petropolitanae III, 85–100.

[B20] Bernsen J. (1986). Dynamic thresholding of grey-level images. In Proceedings of the 6th International Conference on Pattern Recognition (Berlin, Germany), 1251–1255.

[B21] Bery N. Miller A. Rabbitts T. (2020). A potent KRAS macromolecule degrader specifically targeting tumours with mutant KRAS. Nat. Commun. 11, 3233. 10.1038/s41467-020-17022-w 32591521 PMC7319959

[B22] Bieling P. Telley I. A. Surrey T. (2010). A minimal midzone protein module controls Formation and length of Antiparallel Microtubule Overlaps. Cell 142, 420–432. 10.1016/j.cell.2010.06.033 20691901

[B23] Bischoff F. R. Klebe C. Kretschmer J. Wittinghofer A. Ponstingl H. (1994). RanGAP1 induces GTPase activity of nuclear Ras-related ran. Proc. Natl. Acad. Sci. U. S. A. 91, 2587–2591. 10.1073/pnas.91.7.2587 8146159 PMC43414

[B24] Björnsson B. Borrebaeck C. Elander N. Gasslander T. Gawel D. R. Gustafsson M. (2019). Digital twins to personalize medicine. Genome Med. 12, 4. 10.1186/s13073-019-0701-3 31892363 PMC6938608

[B25] Bolger A. M. Lohse M. Usadel B. (2014). Trimmomatic: a flexible trimmer for Illumina sequence data. Bioinformatics 30, 2114–2120. 10.1093/bioinformatics/btu170 24695404 PMC4103590

[B26] Bond M. J. Chu L. Nalawansha D. A. Li K. Crews C. M. (2020). Targeted degradation of oncogenic KRAS(G12C) by VHL-Recruiting PROTACs. ACS Central Sci. 6, 1367–1375. 10.1021/acscentsci.0c00411 32875077 PMC7453568

[B27] Bond C. Hugelier S. Xing J. Sorokina E. M. Lakadamyali M. (2024). Multiplexed DNA-PAINT imaging of the heterogeneity of late Endosome/Lysosome protein composition. bioRxiv, 2024.03.18.585634. 10.1101/2024.03.18.585634

[B28] Borisy G. G. Taylor E. W. (1967). The mechanism of action of colchicine. Binding of colchincine-3H to cellular protein. J. Cell Biol. 34, 525–533. 10.1083/jcb.34.2.525 6068183 PMC2107313

[B29] Bos J. L. (1989). Ras oncogenes in human cancer: a review. Cancer Res. 49, 4682–4689. 2547513

[B30] Bos J. L. (2018). From ras to rap and back, a journey of 35 years. Cold Spring Harb. Perspect. Med. 8, a031468. 10.1101/cshperspect.a031468 28778969 PMC5793741

[B31] Boza-Serrano A. Vrillon A. Minta K. Paulus A. Camprubí-Ferrer L. Garcia M. (2022). Galectin-3 is elevated in CSF and is associated with Aβ deposits and tau aggregates in brain tissue in Alzheimer's disease. Acta Neuropathol. 144, 843–859. 10.1007/s00401-022-02469-6 35895141 PMC9547798

[B32] Brady S. T. (1985). A novel brain ATPase with properties expected for the fast axonal transport motor. Nature 317, 73–75. 10.1038/317073a0 2412134

[B33] Braun A. Dang K. Buslig F. Baird M. A. Davidson M. W. Waterman C. M. (2014). Rac1 and Aurora A regulate MCAK to polarize microtubule growth in migrating endothelial cells. J. Cell Biol. 206, 97–112. 10.1083/jcb.201401063 25002679 PMC4085700

[B34] Brennan I. M. Peters U. Kapoor T. M. Straight A. F. (2007). Polo-like kinase controls vertebrate spindle elongation and cytokinesis. PLoS One 2, e409. 10.1371/journal.pone.0000409 17476331 PMC1853238

[B35] Brennan I. Matov A. Danuser G. Straight A. (2008). Polo-like kinase regulation of Anaphase Spindle elongation. Mol. Biol. Cell 19.

[B36] Brouhard G. J. Rice L. M. (2018). Microtubule dynamics: an interplay of biochemistry and mechanics. Nat. Rev. Mol. Cell Biol. 19, 451–463. 10.1038/s41580-018-0009-y 29674711 PMC6019280

[B37] Buart S. Terry S. Diop M. K. Dessen P. Couvé S. Abdou A. (2021). The Most common VHL point mutation R167Q in hereditary VHL disease interferes with cell plasticity regulation. Cancers 13, 3897. 10.3390/cancers13153897 34359798 PMC8345752

[B38] Burakov A. Vorobjev I. Semenova I. Cowan A. Carson J. Wu Y. (2021). Persistent growth of microtubules at low density. Mol. Biol. Cell 32, 435–445. 10.1091/mbc.E20-08-0546 33439670 PMC8098851

[B39] Cameron L. A. Yang G. Cimini D. Canman J. C. Evgenieva O. K. Khodjakov A. (2006). Kinesin 5-independent poleward flux of kinetochore microtubules in PtK1 cells. J. Cell Biol. 173, 173–179. 10.1083/jcb.200601075 16636143 PMC2063808

[B40] Cappelletti G. Casagrande F. Calogero A. De Gregorio C. Pezzoli G. Cartelli D. (2015). Linking microtubules to Parkinson's disease: the case of parkin. Biochem. Soc. Trans. 43, 292–296. 10.1042/BST20150007 25849932

[B41] Casola L. (2023). Opportunities and challenges for digital twins in biomedical research: proceedings of a Workshop**—**in brief. In National Academies of Sciences, Engineering, and Medicine; National Academy of Engineering; Division on Earth and Life Studies; Division on Engineering and Physical Sciences; Board on Life Sciences; Board on Atmospheric Sciences and Climate; Computer Science and Telecommunications Board; Board on Mathematical Sciences and Analytics.

[B42] Cassimeris L. (2002). The oncoprotein 18/stathmin family of microtubule destabilizers. Curr. Opin. Cell Biol. 14, 18–24. 10.1016/s0955-0674(01)00289-7 11792540

[B43] Cassimeris L. (2009). Microtubule assembly: Lattice GTP to the rescue. Curr. Biol. 19, R174–R176. 10.1016/j.cub.2008.12.035 19243696

[B44] Cassimeris L. U. Walker R. A. Pryer N. K. Salmon E. D. (1987). Dynamic instability of microtubules. BioEssays news Rev. Mol. Cell. Dev. Biol. 7, 149–154. 10.1002/bies.950070403 3318820

[B45] Chan S. Elsheikh A. H. (2018). A machine learning approach for efficient uncertainty quantification using multiscale methods. J. Comput. Phys. 354, 493–511. 10.1016/j.jcp.2017.10.034

[B46] Chen H. (2023). microRNA-Based cancer diagnosis and therapy. Int. J. Mol. Sci. 25, 230. 10.3390/ijms25010230 38203401 PMC10778828

[B47] Chen B. C. Legant W. R. Wang K. Shao L. Milkie D. E. Davidson M. W. (2014). Lattice light-sheet microscopy: imaging molecules to embryos at high spatiotemporal resolution. Science (New York, N.Y.) 346, 1257998. 10.1126/science.1257998 25342811 PMC4336192

[B48] Chen X. Kang R. Kroemer G. Tang D. (2021). Broadening horizons: the role of ferroptosis in cancer. Nat. Rev. Clin. Oncol. 18, 280–296. 10.1038/s41571-020-00462-0 33514910

[B49] Ciciarello M. Mangiacasale R. Thibier C. Guarguaglini G. Marchetti E. Di Fiore B. (2004). Importin beta is transported to spindle poles during mitosis and regulates Ran-dependent spindle assembly factors in mammalian cells. J. Cell Sci. 117, 6511–6522. 10.1242/jcs.01569 15572412

[B50] Cohen-Sharir Y. McFarland J. M. Abdusamad M. Marquis C. Bernhard S. V. Kazachkova M. (2021). Aneuploidy renders cancer cells vulnerable to mitotic checkpoint inhibition. Nature 590, 486–491. 10.1038/s41586-020-03114-6 33505028 PMC8262644

[B52] Conte N. Delaval B. Ginestier C. Ferrand A. Isnardon D. Larroque C. (2003). TACC1–chTOG–Aurora A protein complex in breast cancer. Oncogene 22, 8102–8116. 10.1038/sj.onc.1206972 14603251

[B53] Couchman J. R. Rees D. A. (1979). The behaviour of fibroblasts migrating from chick heart explants: changes in adhesion, locomotion and growth, and in the distribution of actomyosin and fibronectin. J. Cell Sci. 39, 149–165. 10.1242/jcs.39.1.149 575139

[B54] Cuevas-Navarro A. Van R. Cheng A. Urisman A. Castel P. McCormick F. (2021). The RAS GTPase RIT1 compromises mitotic fidelity through spindle assembly checkpoint suppression. Curr. Biol. CB 31, 3915–3924.e9. 10.1016/j.cub.2021.06.030 34237269 PMC8440430

[B55] Czaniecki C. Ryan T. Stykel M. G. Drolet J. Heide J. Hallam R. (2019). Axonal pathology in hPSC-based models of Parkinson's disease results from loss of Nrf2 transcriptional activity at the Map1b gene locus. Proc. Natl. Acad. Sci. U. S. A. 116, 14280–14289. 10.1073/pnas.1900576116 31235589 PMC6628831

[B56] Danecek P. Auton A. Abecasis G. Albers C. A. Banks E. DePristo M. A. (2011). The variant call format and VCFtools. Bioinforma. Oxf. Engl. 27, 2156–2158. 10.1093/bioinformatics/btr330 PMC3137218 21653522

[B57] Danuser G. (2009). Testing the lamella hypothesis: the next steps on the agenda. J. Cell Sci. 122, 1959–1962. 10.1242/jcs.054379 19494124 PMC3656555

[B58] Devred F. Tsvetkov P. O. Barbier P. Allegro D. Horwitz S. B. Makarov A. A. (2008). Stathmin/Op18 is a novel mediator of vinblastine activity. FEBS Lett. 582, 2484–2488. 10.1016/j.febslet.2008.06.035 18588888 PMC2837597

[B59] Dietz R. (1972). An assembly hypothesis of chromosome movement and the changes of the spindle length during anaphase I in spermatocytes of Pales ferruginea. Chromosoma 38, 11–76. 10.1007/BF00319955 4559639

[B60] Dimitrov A. Quesnoit M. Moutel S. Cantaloube I. Poüs C. Perez F. (2008). Detection of GTP-tubulin conformation *in vivo* reveals a role for GTP remnants in microtubule rescues. Science 322, 1353–1356. 10.1126/science.1165401 18927356

[B61] DiNardo C. D. Rosenthal J. Andreeff M. Zernovak O. Kumar P. Gajee R. (2016). Phase 1 dose escalation Study of MDM2 inhibitor DS-3032b in patients with hematological malignancies - preliminary results. Blood 128, 593. 10.1182/blood.v128.22.593.593

[B62] Dinarello C. A. Chusid M. J. Fauci A. S. Gallin J. I. Dale D. C. Wolff S. M. (1976). Effect of prophylactic colchicine therapy on leukocyte function in patients with familial Mediterranean fever. Arthritis Rheumatism 19, 618–622. 10.1002/art.1780190315 779797

[B63] Dixon S. J. Stockwell B. R. (2014). The role of iron and reactive oxygen species in cell death. Nat. Chem. Biol. 10, 9–17. 10.1038/nchembio.1416 24346035

[B64] Eddy D. M. Schlessinger L. (2003). Archimedes: a trial-validated model of diabetes. Diabetes Care 26, 3093–3101. 10.2337/diacare.26.11.3093 14578245

[B51] EFSA Scientific Committee More S. J. Bampidis V. Benford D. Bragard C. Halldorsson T. I. (2022). Guidance on the use of the benchmark dose approach in risk assessment, EFSA Scientific Committee, EFSA Journal. 20:e07584. 10.2903/j.efsa.2022.7584 PMC9593753 36304832

[B65] El-Mogy M. Lam B. Haj-Ahmad T. A. McGowan S. Yu D. Nosal L. (2018). Diversity and signature of small RNA in different bodily fluids using next generation sequencing. BMC Genomics 19, 408. 10.1186/s12864-018-4785-8 29843592 PMC5975555

[B66] Elsheikh A. H. Jackson M. D. Laforce T. C. (2012). Bayesian reservoir history matching considering model and parameter uncertainties. Math. Geosci. 44, 515–543. 10.1007/s11004-012-9397-2

[B67] Ertych N. Stolz A. Stenzinger A. Weichert W. Kaulfuss S. Burfeind P. (2014). Increased microtubule assembly rates influence chromosomal instability in colorectal cancer cells. Nat. Cell Biol. 16, 779–791. 10.1038/ncb2994 24976383 PMC4389786

[B68] Espinosa C. E. S. Singh K. Zhang Z. Sullivan K. Gitego N. Rigby M. (2025). Abstract 4378: BBO-11818, an orally bioavailable, highly potent and non-covalent pan-KRAS inhibitor demonstrates robust anti-tumor activity in KRAS-mutant preclinical models. Cancer Res. 85, 4378. 10.1158/1538-7445.am2025-4378

[B69] Fifre A. Sponne I. Koziel V. Kriem B. Yen Potin F. T. Bihain B. E. (2006). Microtubule-associated protein MAP1A, MAP1B, and MAP2 proteolysis during soluble amyloid beta-peptide-induced neuronal apoptosis. Synergistic involvement of calpain and caspase-3. J. Biol. Chem. 281, 229–240. 10.1074/jbc.M507378200 16234245

[B70] Fourier J. B. J. (1822). Théorie Analytique de la Chaleur. Paris: Chez Firmin Didot, Pere et Fils.

[B71] Gaetz J. Kapoor T. M. (2004). Dynein/dynactin regulate metaphase spindle length by targeting depolymerizing activities to spindle poles. J. Cell Biol. 166, 465–471. 10.1083/jcb.200404015 15314063 PMC1401226

[B72] Gaglio T. Dionne M. A. Compton D. A. (1997). Mitotic spindle poles are organized by structural and motor proteins in addition to centrosomes. J. Cell Biol. 138, 1055–1066. 10.1083/jcb.138.5.1055 9281583 PMC2136753

[B73] Galigniana M. D. Morishima Y. Gallay P. A. Pratt W. B. (2004). Cyclophilin-A is bound through its peptidylprolyl isomerase domain to the cytoplasmic dynein motor protein complex. J. Biol. Chem. 279, 55754–55759. 10.1074/jbc.M406259200 15496417

[B74] Galletti G. Matov A. Beltran H. Fontugne J. Miguel Mosquera J. Cheung C. (2014). ERG induces taxane resistance in castration-resistant prostate cancer. Nat. Commun. 5, 5548. 10.1038/ncomms6548 25420520 PMC4244604

[B75] Gan H. Lin L. Hu N. Yang Y. Gao Y. Pei Y. (2019). KIF2C exerts an oncogenic role in nonsmall cell lung cancer and is negatively regulated by miR-325-3p. Cell Biochem. Funct. 37, 424–431. 10.1002/cbf.3420 31328811

[B76] Gandaglia G. Abdollah F. Schiffmann J. Trudeau V. Shariat S. F. Kim S. P. (2014). Distribution of metastatic sites in patients with prostate cancer: a population-based analysis. Prostate 74, 210–216. 10.1002/pros.22742 24132735

[B77] Gao D. Vela I. Sboner A. Iaquinta P. J. Karthaus W. R. Gopalan A. (2014). Organoid cultures derived from patients with advanced prostate cancer. Cell 159, 176–187. 10.1016/j.cell.2014.08.016 25201530 PMC4237931

[B78] Garcez P. P. Diaz-Alonso J. Crespo-Enriquez I. Castro D. Bell D. Guillemot F. (2015). Cenpj/CPAP regulates progenitor divisions and neuronal migration in the cerebral cortex downstream of Ascl1. Nat. Commun. 6, 6474. 10.1038/ncomms7474 25753651 PMC4366522

[B79] Garcia-Bermudez J. Birsoy K. (2021). A mitochondrial gatekeeper that helps cells escape death by ferroptosis. Nature 593, 514–515. 10.1038/d41586-021-01203-8 33981062

[B80] Gard D. L. Kirschner M. W. (1987). A microtubule-associated protein from Xenopus eggs that specifically promotes assembly at the plus-end. J. Cell Biol. 105, 2203–2215. 10.1083/jcb.105.5.2203 2890645 PMC2114854

[B81] Gatlin J. C. Matov A. Groen A. C. Needleman D. J. Maresca T. J. Danuser G. (2009). Spindle fusion requires dynein-mediated sliding of oppositely oriented microtubules. Curr. Biol. 19, 287–296. 10.1016/j.cub.2009.01.055 19230671 PMC2709244

[B82] Gatlin J. C. Matov A. Danuser G. Mitchison T. J. Salmon E. D. (2010). Directly probing the mechanical properties of the spindle and its matrix. J. Cell Biol. 188, 481–489. 10.1083/jcb.200907110 20176922 PMC2828919

[B83] Genbacev O. Krtolica A. Kaelin W. Fisher S. J. (2001). Human cytotrophoblast expression of the von Hippel-Lindau protein is downregulated during uterine invasion *in situ* and upregulated by hypoxia *in vitro* . Dev. Biol. 233, 526–536. 10.1006/dbio.2001.0231 11336512

[B84] Giannakakou P. Sackett D. L. Kang Y. K. Zhan Z. Buters J. T. Fojo T. (1997). Paclitaxel-resistant human ovarian cancer cells have mutant beta-tubulins that exhibit impaired paclitaxel-driven polymerization. J. Biol. Chem. 272, 17118–17125. 10.1074/jbc.272.27.17118 9202030

[B85] Giannakakou P. Gussio R. Nogales E. Downing K. H. Zaharevitz D. Bollbuck B. (2000a). A common pharmacophore for epothilone and taxanes: molecular basis for drug resistance conferred by tubulin mutations in human cancer cells. Proc. Natl. Acad. Sci. U. S. A. 97, 2904–2909. 10.1073/pnas.040546297 10688884 PMC16028

[B86] Giannakakou P. Sackett D. L. Ward Y. Webster K. R. Blagosklonny M. V. Fojo T. (2000b). p53 is associated with cellular microtubules and is transported to the nucleus by dynein. Nat. Cell Biol. 2, 709–717. 10.1038/35036335 11025661

[B87] Giannakakou P. Nakano M. Nicolaou K. C. O'Brate A. Yu J. Blagosklonny M. V. (2002). Enhanced microtubule-dependent trafficking and p53 nuclear accumulation by suppression of microtubule dynamics. Proc. Natl. Acad. Sci. U. S. A. 99, 10855–10860. 10.1073/pnas.132275599 12145320 PMC125062

[B88] Gierke S. Wittmann T. (2012). EB1-recruited microtubule +TIP complexes coordinate protrusion dynamics during 3D epithelial remodeling. Curr. Biol. CB 22, 753–762. 10.1016/j.cub.2012.02.069 22483942 PMC3350573

[B89] Gijzen L. Yousef Yengej F. A. Schutgens F. Vormann M. K. Ammerlaan C. M. E. Nicolas A. (2021). Culture and analysis of kidney tubuloids and perfused tubuloid cells-on-a-chip. Nat. Protoc. 16, 2023–2050. 10.1038/s41596-020-00479-w 33674788

[B90] Goldstein A. S. Drake J. M. Burnes D. L. Finley D. S. Zhang H. Reiter R. E. (2011). Purification and direct transformation of epithelial progenitor cells from primary human prostate. Nat. Protoc. 6, 656–667. 10.1038/nprot.2011.317 21527922 PMC3092477

[B91] Gonçalves A. Braguer D. Kamath K. Martello L. Briand C. Horwitz S. (2001). Resistance to Taxol in lung cancer cells associated with increased microtubule dynamics. Proc. Natl. Acad. Sci. U. S. A. 98, 11737–11742. 10.1073/pnas.191388598 11562465 PMC58799

[B92] Green Y. S. Ferreira dos Santos M. C. Fuja D. G. Reichert E. C. Campos A. R. Cowman S. J. (2022). ISCA2 inhibition decreases HIF and induces ferroptosis in clear cell renal carcinoma. Oncogene 41, 4709–4723. 10.1038/s41388-022-02460-1 36097192 PMC9568429

[B93] Groen A. (2008). Chromatin dependent microtubule assembly during meiosis. Harvard University. Advisor: Timothy Mitchison.

[B94] Groen A. C. Maresca T. J. Gatlin J. C. Salmon E. D. Mitchison T. J. (2009). Functional overlap of microtubule assembly factors in chromatin-promoted spindle assembly. Mol. Biol. Cell 20, 2766–2773. 10.1091/mbc.e09-01-0043 19369413 PMC2688555

[B95] Gros O. J. Damstra H. G. J. Kapitein L. C. Akhmanova A. Berger F. (2021). Dynein self-organizes while translocating the centrosome in T-cells. Mol. Biol. Cell 32, 855–868. 10.1091/mbc.E20-10-0668 33689395 PMC8108531

[B96] Guan G. Cannon R. D. Coates D. E. Mei L. (2023). Effect of the Rho-Kinase/ROCK signaling pathway on cytoskeleton components. Genes 14, 272. 10.3390/genes14020272 36833199 PMC9957420

[B97] Gudimchuk N. B. McIntosh J. R. (2021). Regulation of microtubule dynamics, mechanics and function through the growing tip. Nat. Rev. Mol. Cell Biol. 22, 777–795. 10.1038/s41580-021-00399-x 34408299

[B98] Haber M. Burkhart C. A. Regl D. L. Madafiglio J. Norris M. D. Horwitz S. B. (1995). Altered expression of M beta 2, the class II beta-tubulin isotype, in a murine J774.2 cell line with a high level of taxol resistance. J. Biol. Chem. 270, 31269–31275. 10.1074/jbc.270.52.31269 8537394

[B99] Hajka D. Budziak B. Pietras Ł. Duda P. McCubrey J. A. Gizak A. (2021). GSK3 as a regulator of cytoskeleton architecture: consequences for health and disease. Cells 10, 2092. 10.3390/cells10082092 34440861 PMC8393567

[B100] Hall C. A. Meyer W. W. (1976). Optimal error bounds for cubic spline interpolation. J. Approx. Theory 16, 105–122. 10.1016/0021-9045(76)90040-x

[B101] Hamaguchi Y. Toriyama M. Sakai H. Hiramoto Y. (1987). Redistribution of fluorescently labeled tubulin in the mitotic apparatus of sand dollar eggs and the effects of taxol. Cell Struct. Funct. 12, 43–52. 10.1247/csf.12.43 2882862

[B102] Han Y. Jiao J. Weissman T. (2016). Minimax rate-optimal estimation of KL divergence between discrete distributions. Int. Symposium Inf. Theory its Appl. 2016, 256–260. 29457152 PMC5812299

[B103] Hangauer M. J. Viswanathan V. S. Ryan M. J. Bole D. Eaton J. K. Matov A. (2017). Drug-tolerant persister cancer cells are vulnerable to GPX4 inhibition. Nature 551, 247–250. 10.1038/nature24297 29088702 PMC5933935

[B104] Harkcom W. T. Ghosh A. K. Sung M. S. Matov A. Brown K. D. Giannakakou P. (2014). NAD+ and SIRT3 control microtubule dynamics and reduce susceptibility to antimicrotubule agents. Proc. Natl. Acad. Sci. U. S. A. 111, E2443–E2452. 10.1073/pnas.1404269111 24889606 PMC4066477

[B105] Harrill J. A. Everett L. J. Haggard D. E. Sheffield T. Bundy J. L. Willis C. M. (2021). High-Throughput transcriptomics platform for screening environmental chemicals. Toxicol. Sci. Official J. Soc. Toxicol. 181, 68–89. 10.1093/toxsci/kfab009 PMC10194851 33538836

[B106] He J. Qiu Z. Fan J. Xie X. Sheng Q. Sui X. (2024). Drug tolerant persister cell plasticity in cancer: a revolutionary strategy for more effective anticancer therapies. Signal Transduct. Target. Ther. 9, 209. 10.1038/s41392-024-01891-4 39138145 PMC11322379

[B107] Heath I. B. (1980). Behavior of kinetochores during mitosis in the fungus Saprolegnia ferax. J. Cell Biol. 84, 531–546. 10.1083/jcb.84.3.531 7358791 PMC2110583

[B108] Heger I. (1858). Mathematisch-naturwissenschaftliche classe. Denkschr. Kais. Akad. Wiss. 14, 1–122.

[B109] Henrich S. E. McMahon K. M. Plebanek M. P. Calvert A. E. Feliciano T. J. Parrish S. (2020). Prostate cancer extracellular vesicles mediate intercellular communication with bone marrow cells and promote metastasis in a cholesterol-dependent manner. J. Extracell. Vesicles 10, e12042. 10.1002/jev2.12042 33408816 PMC7775568

[B110] Hermite C. (1864). Sur un nouveau développement en série de fonctions. Comptes rendus l'Académie Sci. 58 (93–100), 266–273.

[B111] Hesterberg T. C. Moore D. S. Monaghan S. Clipson A. Epstein R. (2005). Bootstrap methods and permutation tests. In Introduction to the Practice of Statistics (W. H. Freeman).

[B112] Höing S. Yeh T. Y. Baumann M. Martinez N. E. Habenberger P. Kremer L. (2018). Dynarrestin, a novel inhibitor of Cytoplasmic dynein. Cell Chem. Biol. 25, 357–369.e6. 10.1016/j.chembiol.2017.12.014 29396292 PMC8543760

[B113] Holland P. W. Welsch R. E. (1977). Robust regression using iteratively reweighted least-squares. Commun. Statistics Theory Methods 6, 813–827. 10.1080/03610927708827533

[B114] Hong D. S. Fakih M. G. Strickler J. H. Desai J. Durm G. A. Shapiro G. I. (2020). KRAS(G12C) inhibition with Sotorasib in advanced solid tumors. N. Engl. J. Med. 383, 1207–1217. 10.1056/NEJMoa1917239 32955176 PMC7571518

[B115] Houghtaling B. R. Yang G. Matov A. Danuser G. Kapoor T. M. (2009). Op18 reveals the contribution of non-kinetochore microtubules to the dynamic Organization of the vertebrate meiotic spindle. Proc. Natl. Acad. Sci. U. S. A. 106, 15338–15343. 10.1073/pnas.0902317106 19706424 PMC2741252

[B116] Hu Y. Xie Q. Wu X. Liu W. Li D. Li C. (2022). Tension of plus-end tracking protein Clip170 confers directionality and aggressiveness during breast cancer migration. Cell Death and Dis. 13, 856. 10.1038/s41419-022-05306-6 PMC9547975 36209218

[B117] Hyman A. A. Salser S. Drechsel D. N. Unwin N. Mitchison T. J. (1992). Role of GTP hydrolysis in microtubule dynamics: information from a slowly hydrolyzable analogue, GMPCPP. Mol. Biol. Cell 3, 1155–1167. 10.1091/mbc.3.10.1155 1421572 PMC275679

[B118] Inoué S. (1959). Motility of cilia and the mechanism of mitosis. Rev. Mod. Phys. 31, 402–408. 10.1103/revmodphys.31.402

[B119] Inoué S. Ritter H. Jr. (1975). Dynamics of mitotic spindle organization and function. Soc. General Physiologists Ser. 30, 3–30. 1103302

[B120] Inoué S. Salmon E. D. (1995). Force generation by microtubule assembly/disassembly in mitosis and related movements. Mol. Biol. Cell 6, 1619–1640. 10.1091/mbc.6.12.1619 8590794 PMC301321

[B121] Inoue H. Kanda T. Hayashi G. Munenaga R. Yoshida M. Hasegawa K. (2024). A MAP1B–cortactin–Tks5 axis regulates TNBC invasion and tumorigenesis. J. Cell Biol. 223, e202303102. 10.1083/jcb.202303102 38353696 PMC10866687

[B122] Jallepalli P. V. Lengauer C. (2001). Chromosome segregation and cancer: cutting through the mystery. Nat. Rev. Cancer 1, 109–117. 10.1038/35101065 11905802

[B123] Jänne P. A. Bigot F. Papadopoulos K. Eberst L. Sommerhalder D. Lebellec L. (2023). Abstract PR014: preliminary safety and anti-tumor activity of RMC-6291, a first-in-class, tri-complex KRASG12C(ON) inhibitor, in patients with or without prior KRASG12C(OFF) inhibitor treatment. Mol. Cancer Ther. 22, PR014. 10.1158/1535-7163.targ-23-pr014

[B124] Janssen A. Beerling E. Medema R. van Rheenen J. (2013). Intravital FRET imaging of tumor cell viability and mitosis during chemotherapy. PLoS One 8, e64029. 10.1371/journal.pone.0064029 23691140 PMC3654962

[B128] Ježek J. Cooper K. F. Strich R. (2018). Reactive oxygen species and mitochondrial dynamics: the Yin and Yang of mitochondrial dysfunction and cancer progression. Antioxidants Basel, Switz. 7, 13. 10.3390/antiox7010013 29337889 PMC5789323

[B129] Jiang W. Zheng L. Yan Q. Chen L. Wang X. (2019). MiR-532-3p inhibits metastasis and proliferation of non-small cell lung cancer by targeting FOXP3. J. B.U.ON, Official J. Balkan Union Oncol. 24, 2287–2293. 31983096

[B130] Jiang W. Lin M. Wang Z. (2020). Dioscin: a new potential inhibitor of Skp2 for cancer therapy. eBioMedicine 51, 102593. 10.1016/j.ebiom.2019.12.002 31901855 PMC6948171

[B131] Jordan M. A. Wilson L. (2004). Microtubules as a target for anticancer drugs. Nat. Rev. Cancer. 4, 253–265. 10.1038/nrc1317 15057285

[B132] Joseph J. Liu S. T. Jablonski S. A. Yen T. J. Dasso M. (2004). The RanGAP1-RanBP2 complex is essential for microtubule-kinetochore interactions *in vivo* . Curr. Biol. CB 14, 611–617. 10.1016/j.cub.2004.03.031 15062103

[B133] Judson R. S. Kavlock R. J. Setzer R. W. Hubal E. A. Martin M. T. Knudsen T. B. (2011). Estimating toxicity-related biological pathway altering doses for high-throughput chemical risk assessment. Chem. Res. Toxicol. 24, 451–462. 10.1021/tx100428e 21384849

[B134] Jungius J. (1622). Heuretica. Soc. Ereun.

[B135] Jurj A. Fontana B. Varani G. Calin G. A. (2024). Small molecules targeting microRNAs: new opportunities and challenges in precision cancer therapy. Trends Cancer 10, 809–824. 10.1016/j.trecan.2024.06.006 39107162 PMC11961049

[B136] Kaelin W. G. Jr. Ratcliffe P. J. (2008). Oxygen sensing by metazoans: the central role of the HIF hydroxylase pathway. Mol. Cell 30, 393–402. 10.1016/j.molcel.2008.04.009 18498744

[B137] Kanakkanthara A. Miller J. H. (2012). MicroRNAs: novel mediators of resistance to microtubule-targeting agents. Cancer Treat. Rev. 39, 161–170. 10.1016/j.ctrv.2012.07.005 22902296

[B138] Kanakkanthara A. Wilmes A. O'Brate A. Escuin D. Chan A. Gjyrezi A. (2011). Peloruside- and laulimalide-resistant human ovarian carcinoma cells have betaI-tubulin mutations and altered expression of betaII- and betaIII-tubulin isotypes. Mol. Cancer Ther. 10, 1419–1429. 21653684 10.1158/1535-7163.MCT-10-1057 PMC3158586

[B139] Kant I. (1781). Critik der reinen Vernunft. Königsberg.

[B140] Kant I. (1797). Die Metaphysik der Sitten. Königsberg.

[B141] Kavallaris M. (2010). Microtubules and resistance to tubulin-binding agents. Nat. Rev. Cancer 10, 194–204. 10.1038/nrc2803 20147901

[B142] Kazi A. Xiang S. Yang H. Delitto D. Trevino J. Jiang R. H. Y. (2018). GSK3 suppression upregulates β-catenin and c-Myc to abrogate KRas-dependent tumors. Nat. Commun. 9, 5154. 10.1038/s41467-018-07644-6 30514931 PMC6279809

[B143] Keshari K. R. Sriram R. Van Criekinge M. Wilson D. M. Wang Z. J. Vigneron D. B. (2013). Metabolic reprogramming and validation of hyperpolarized 13C lactate as a prostate cancer biomarker using a human prostate tissue slice culture bioreactor. Prostate 73, 1171–1181. 10.1002/pros.22665 23532911 PMC3976546

[B144] Kim T. Croce C. M. (2023). MicroRNA: trends in clinical trials of cancer diagnosis and therapy strategies. Exp. and Mol. Med. 55, 1314–1321. 10.1038/s12276-023-01050-9 37430087 PMC10394030

[B145] Kingwell K. (2018). Anticancer drugs: flipping the switch for selective GSK3 inhibition. Nat. Rev. Drug Discov. 17, 314. 10.1038/nrd.2018.63 29700491

[B146] Kirschner M. W. (1978). Microtubule assembly and nucleation. Int. Rev. Cytol. 54, 1–71. 10.1016/s0074-7696(08)60164-3 391755

[B147] Kirschner M. Mitchison T. (1986). Beyond self-assembly: from microtubules to morphogenesis. Cell 45, 329–342. 10.1016/0092-8674(86)90318-1 3516413

[B148] Kitayama H. Sugimoto Y. Matsuzaki T. Ikawa Y. Noda M. (1989). A ras-related gene with transformation suppressor activity. Cell 56, 77–84. 10.1016/0092-8674(89)90985-9 2642744

[B149] Klein E. (1878). Observations on the structure of cells and nuclei. J. Cell Sci. s2-18, 315–339. 10.1242/jcs.s2-18.71.315

[B150] Kolmogorov A. N. (1933). Sulla determinazione empírica di uma legge di distribuzione. G. dell’Istituto Ital. degli Attuari 4, 83–91.

[B151] Komarova Y. De Groot C. O. Grigoriev I. Gouveia S. M. Munteanu E. L. Schober J. M. (2009). Mammalian end binding proteins control persistent microtubule growth. J. Cell Biol. 184, 691–706. 10.1083/jcb.200807179 19255245 PMC2686402

[B152] Koo B. K. Sasselli V. Clevers H. (2013). Retroviral gene expression control in primary organoid cultures. Curr. Protoc. Stem Cell Biol. 27–5A.6.8. 10.1002/9780470151808.sc05a06s27 24510288

[B153] Kopra K. Mahran R. Yli-Hollo T. Tabata S. Vuorinen E. Fujii Y. (2023). Homogeneous luminescent quantitation of cellular guanosine and adenosine triphosphates (GTP and ATP) using QT-LucGTP&ATP assay. Anal. Bioanal. Chem. 415, 6689–6700. 10.1007/s00216-023-04944-9 37714971 PMC10598090

[B154] Koshland D. E. Mitchison T. J. Kirschner M. W. (1988). Polewards chromosome movement driven by microtubule depolymerization *in vitro* . Nature 331, 499–504. 10.1038/331499a0 3340202

[B155] Kullback S. Leibler R. A. (1951). On information and sufficiency. Ann. Math. Statistics 79-86, 78.

[B156] Kumar P. Lyle K. S. Gierke S. Matov A. Danuser G. Wittmann T. (2009). GSK3beta phosphorylation modulates CLASP-microtubule association and lamella microtubule attachment. J. Cell Biol. 184, 895–908. 10.1083/jcb.200901042 19289791 PMC2699158

[B157] Kuo Y. W. Howard J. (2021). Cutting, amplifying, and aligning microtubules with severing enzymes. Trends Cell Biol. 31, 50–61. 10.1016/j.tcb.2020.10.004 33183955 PMC7749064

[B158] Kwon M. Bagonis M. Danuser G. Pellman D. (2015). Direct microtubule-binding by Myosin-10 orients centrosomes toward retraction fibers and subcortical actin clouds. Dev. Cell 34, 323–337. 10.1016/j.devcel.2015.06.013 26235048 PMC4672950

[B159] Laks D. R. Oses-Prieto J. A. Alvarado A. G. Nakashima J. Chand S. Azzam D. B. (2018). A molecular cascade modulates MAP1B and confers resistance to mTOR inhibition in human glioblastoma. Neuro-Oncology 20, 764–775. 10.1093/neuonc/nox215 29136244 PMC5961175

[B160] Laplace P.-S. (1810). Memoire sur les approximations des formules qui sont fonctions de tres grands nombres et sur leur application aux probabilites. Mémoires l'Académie R. Sci., 353–415.

[B161] Laubenbacher R. Mehrad B. Shmulevich I. Trayanova N. (2024). Digital twins in medicine. Nat. Comput. Sci. 4, 184–191. 10.1038/s43588-024-00607-6 38532133 PMC11102043

[B162] Le Grand M. Rovini A. Bourgarel-Rey V. Honore S. Bastonero S. Braguer D. (2014). ROS-mediated EB1 phosphorylation through Akt/GSK3β pathway: implication in cancer cell response to microtubule-targeting agents. Oncotarget 5, 3408–3423. 10.18632/oncotarget.1982 24930764 PMC4102819

[B163] Lee S. H. Hu W. Matulay J. T. Silva M. V. Owczarek T. B. Kim K. (2018). Tumor evolution and drug response in patient-derived organoid models of bladder cancer. Cell. 173, 515–528. 10.1016/j.cell.2018.03.017 29625057 PMC5890941

[B164] Li H. (2011). A statistical framework for SNP calling, mutation discovery, association mapping and population genetical parameter estimation from sequencing data. Bioinforma. Oxf. Engl. 27, 2987–2993. 10.1093/bioinformatics/btr509 21903627 PMC3198575

[B165] Li H. Durbin R. (2010). Fast and accurate long-read alignment with Burrows-Wheeler transform. Bioinforma. Oxf. Engl. 26, 589–595. 10.1093/bioinformatics/btp698 20080505 PMC2828108

[B166] Li W. Febbo P. (2013). Abstract A35: Docetaxel resistance mechanisms in prostate cancer: p38 MAPK signaling pathway and microtubule dynamics. Cancer Res. 72, A35. 10.1158/1538-7445.prca2012-a35

[B167] Li H. Handsaker B. Wysoker A. Fennell T. Ruan J. Homer N. (2009). The Sequence Alignment/Map format and SAMtools. Bioinforma. Oxf. Engl. 25, 2078–2079. 10.1093/bioinformatics/btp352 PMC2723002 19505943

[B168] Li X. Yao R. Yue L. Qiu W. Qi W. Liu S. (2014). FOXM1 mediates resistance to docetaxel in gastric cancer *via* up-regulating Stathmin. J. Cell Mol. Med. 18, 811–823. 10.1111/jcmm.12216 24628949 PMC4119387

[B169] Linda W. (2005). Microtubule-depolymerizing kinesins. Curr. Opin. Cell Biol. 17, 82–88. 10.1016/j.ceb.2004.12.003 15661523

[B170] Lindeberg T. (1993). Detecting salient blob-like image structures and their scales with a scale-space primal sketch: a method for focus-of-attention. Int. J. Comput. Vis. 11, 283–318. 10.1007/bf01469346

[B171] Linn S. C. Giaccone G. Pinedo H. M. (1994). Complete remission of metastatic colorectal cancer: a pitfall in a multidrug resistance reversal trial. Lancet London, Engl. 343, 1648–1649. 10.1016/s0140-6736(94)93107-0 7911960

[B172] Lloyd D. (2002). Alteration of microtubule function increases p53-dependent apoptosis. Trends Pharmacol. Sci. 23, 500–501. 10.1016/s0165-6147(02)02123-5

[B173] Long J. B. Bagonis M. Lowery L. A. Lee H. Danuser G. Van Vactor D. (2013). Multiparametric analysis of CLASP-interacting protein functions during interphase microtubule dynamics. Mol. Cell. Biol. 33, 1528–1545. 10.1128/MCB.01442-12 23382075 PMC3624261

[B174] Luo J. Hu Q. Gou M. Liu X. Qin Y. Zhu J. (2021). Expression of microtubule-associated proteins in relation to prognosis and efficacy of immunotherapy in non-small cell lung cancer. Front. Oncol. 11, 680402. 10.3389/fonc.2021.680402 34660263 PMC8517487

[B175] Lyle K. Kumar P. Wittmann T. (2009a). SnapShot: Microtubule regulators I. Cell 136 (380), 380, 380.e1–380.e381. 10.1016/j.cell.2009.01.010 19167337 PMC4019432

[B176] Lyle K. Kumar P. Wittmann T. (2009b). SnapShot: Microtubule regulators II. Cell 136 (566), 566, 566.e1–566.e561. 10.1016/j.cell.2009.01.011 19203588 PMC4019436

[B177] Maciag A. E. Stice J. P. Wang B. Sharma A. K. Chan A. H. Lin K. (2025). Discovery of BBO-8520, a first-in-class direct and covalent dual inhibitor of GTP-Bound (ON) and GDP-Bound (OFF) KRASG12C. Cancer Discov. 15, 578–594. 10.1158/2159-8290.CD-24-0840 39642212 PMC11873722

[B178] Maddox P. Straight A. Coughlin P. Mitchison T. J. Salmon E. D. (2003). Direct observation of microtubule dynamics at kinetochores in Xenopus extract spindles: implications for spindle mechanics. J. Cell Biol. 162, 377–382. 10.1083/jcb.200301088 12900391 PMC2172681

[B179] Malesinski S. Tsvetkov P. O. Kruczynski A. Peyrot V. Devred F. (2015). Stathmin potentiates vinflunine and inhibits Paclitaxel activity. PLoS One 10, e0128704. 10.1371/journal.pone.0128704 26030092 PMC4451147

[B180] Manning A. L. Ganem N. J. Bakhoum S. F. Wagenbach M. Wordeman L. Compton D. A. (2007). The kinesin-13 proteins Kif2a, Kif2b, and Kif2c/MCAK have distinct roles during mitosis in human cells. Mol. Biol. Cell 18, 2970–2979. 10.1091/mbc.e07-02-0110 17538014 PMC1949365

[B181] Mao C. Liu X. Zhang Y. Lei G. Yan Y. Lee H. (2021). DHODH-mediated ferroptosis defence is a targetable vulnerability in cancer. Nature 593, 586–590. 10.1038/s41586-021-03539-7 33981038 PMC8895686

[B182] Maqbool M. Bekele F. Fekadu G. (2022). Treatment strategies against triple-negative breast cancer: an updated review. Breast Cancer (Dove Med. Press) 14, 15–24. 10.2147/BCTT.S348060 35046722 PMC8760999

[B183] Martin S. K. Kamelgarn M. Kyprianou N. (2014). Cytoskeleton targeting value in prostate cancer treatment. Am. J. Clin. Exp. Urol. 2, 15–26. 25374905 PMC4219288

[B184] Martino M. T. D. Tagliaferri P. Tassone P. (2025). MicroRNA in cancer therapy: breakthroughs and challenges in early clinical applications. J. Exp. and Clin. Cancer Res. CR 44, 126. 10.1186/s13046-025-03391-x 40259326 PMC12010629

[B185] Matov A. (2006). Evidence-based approach to personalized medicine. Scripps Res.

[B186] Matov A. (2009). Analyses of microtubule dynamics as quantitative readout of cytoskeletal processes, Scripps Res. 1–217.

[B187] Matov A. (2024a). Analysis of genome-wide cell-free DNA fragment length distributions in colorectal cancer. medRxiv:2024.24310568.

[B188] Matov A. (2024b). Analysis of multiple myeloma drug efficacy. medRxiv:2024.24311450.

[B189] Matov A. (2024c). Analysis of unstructured high-density crowded scenes for crowd monitoring. arXiv:2408.11836.

[B190] Matov A. (2024d). Colorectal cancer DNA fragmentation patterns as image datasets. SSRN:2024.4909309.

[B191] Matov A. (2024e). Computational analysis of treatment resistant cancer cells. medRxiv:2024.24312813.

[B192] Matov A. (2024f). Measurements of contractility in actin convergence zone and KIF11-Inhibited mitotic spindles. bioRxiv:2024.601275.

[B193] Matov A. (2024g). Microtubule regulation in cancer cells *SSRN*:2024.5072927. 10.2139/ssrn.5072927 PMC13543818 42699073

[B194] Matov A. (2024h). Mitosis, cytoskeleton regulation, and drug resistance in receptor triple-negative breast cancer. arXiv:2407.19112. 10.48550/arXiv.2407.19112

[B195] Matov A. (2024i). Quantitative microscopy in medicine. medRxiv:2024.24311304.

[B196] Matov A. (2024j). Quantitative video microscopy in medicine. SSRN:2024.4909311.

[B197] Matov A. (2024k). Real-time image analysis software suitable for resource-constrained computing. arXiv:2407.15735. 10.48550/arXiv.2407.15735

[B198] Matov A. (2024l). Urinary biomarkers for lung cancer detection. medRxiv:2024.24311186. 10.1016/j.jlb.2026.100456 PMC13084665 42004687

[B199] Matov A. (2025a). Microtubule dysregulation in prostate cancer metastasis to the vertebrae *SSRN*:2025.5112427.

[B200] Matov A. (2025b). Quantitative cell division and migration in medicine. *SSRN*:2025.5130515.

[B204] Matov A. Applegate K. Kumar P. Thoma C. R. Krek W. Danuser G. (2010). Analysis of microtubule dynamic instability using a plus end growth marker. Nat. Methods 7, 761–768. 10.1038/nmeth.1493 20729842 PMC3032800

[B201] Matov A. Danuser G. (2004). Tracking faint features in fluorescent speckle microscopy to probe microtubule flux in mitotic spindles. Scripps Res.

[B208] Matov A. de Rooij J. Gatlin J. C. Rohrberg J. Ahmad N. Aggarwal R. (2016). Investigating microtubule growth dynamics in patient-derived metastatic prostate cancer cells. Cancer Res. 76, 243. 10.1158/1538-7445.am2016-243

[B205] Matov A. Edvall M. M. Yang G. Danuser G. (2011). Optimal-flow minimum-cost correspondence assignment in particle flow tracking. Computer Vision and Image Understanding 115, 531–540. 10.1016/j.cviu.2011.01.001 21720496 PMC3123713

[B206] Matov A. Kimovski D. Galletti G. Chan N. Pera B. Harkcom W. T. (2013). Computational analysis of microtubule dynamics for personalized cancer therapy. Cancer Res. 73, 2888. 10.1158/1538-7445.am2013-2888

[B207] Matov A. Rohrberg J. Goga A. Wittmann T. (2015). Investigating microtubule growth dynamics and spindle positioning regulation in breast cancer cells. Mol. Biol. Cell 26.

[B203] Matov A. Thoma C. Krek W. Danuser G. (2006). Von hippel-lindau protein induced variation of microtubule dynamic instability probed by automated GFPlabeled EB3 spot detection and tracking. Mol. Biol. Cell 17.

[B202] Matov A. Yang G. Danuser G. (2005). Analysis of the movement of microtubule plus-end tracking protein EB1. Mol. Biol. Cell 16.

[B209] Maund S. L. Nolley R. Peehl D. M. (2014). Optimization and comprehensive characterization of a faithful tissue culture model of the benign and malignant human prostate. Laboratory investigation; A J. Tech. Methods Pathology 94, 208–221. 10.1038/labinvest.2013.141 24296879 PMC3946793

[B210] Maxwell P. H. Pugh C. W. Ratcliffe P. J. (2001). The pVHL-hIF-1 system. A key mediator of oxygen homeostasis. Adv. Exp. Med. Biol. 502, 365–376. 10.1007/978-1-4757-3401-0_24 11950150

[B211] Mayer T. U. Kapoor T. M. Haggarty S. J. King R. W. Schreiber S. L. Mitchison T. J. (1999). Small molecule inhibitor of mitotic spindle bipolarity identified in a phenotype-based screen. Science 286, 971–974. 10.1126/science.286.5441.971 10542155

[B212] McGrail D. J. Khambhati N. N. Qi M. X. Patel K. S. Ravikumar N. Brandenburg C. P. (2015). Alterations in ovarian cancer cell adhesion drive taxol resistance by increasing microtubule dynamics in a FAK-dependent manner. Sci. Rep. 5, 9529. 10.1038/srep09529 25886093 PMC4400875

[B213] McLaren W. Gil L. Hunt S. E. Riat H. S. Ritchie G. R. Thormann A. (2016). The ensembl variant effect predictor. Genome Biol. 17, 122. 10.1186/s13059-016-0974-4 27268795 PMC4893825

[B214] Mertens S. Huismans M. A. Verissimo C. S. Ponsioen B. Overmeer R. Proost N. (2023). Drug-repurposing screen on patient-derived organoids identifies therapy-induced vulnerability in KRAS-mutant colon cancer. Cell Rep. 42, 112324. 10.1016/j.celrep.2023.112324 37000626

[B125] Milunović-Jevtić A. M. Tripathi A. Groen A. C. Ishihara K. Marina N. Mitchison T. J. (2013). Investigating microtubule growth dynamics in discrete cytoplasmic volumes. Mol. Biol. Cell 24.

[B126] Milunović-Jevtić A. M. Matov A. Hazel J. W. Groen A. C. Ishihara K. Mitchison T. J. (2014). Microtubule dynamics are modulated by changes in cytoplasmic volume: a possible mechanism for mitotic spindle scaling. Mol. Biol. Cell 25.

[B127] Milunović-Jevtić A. M. Jevtić P. Matov A. Tripathi A. Krutkramelis K. Levy D. L. (2015). Cell size-dependent changes in microtubule dynamics: a possible mechanism for mitotic spindle scaling during development. Mol. Biol. Cell 26.

[B215] Mimori-Kiyosue Y. Grigoriev I. Lansbergen G. Sasaki H. Matsui C. Severin F. (2005). CLASP1 and CLASP2 bind to EB1 and regulate microtubule plus-end dynamics at the cell cortex, J. Cell Biol. 168:141–153. 10.1083/jcb.200405094 15631994 PMC2171674

[B216] Mistry S. J. Atweh G. F. (2006). Therapeutic interactions between stathmin inhibition and chemotherapeutic agents in prostate cancer. Mol. Cancer Ther. 5, 3248–3257. 10.1158/1535-7163.MCT-06-0227 17172428

[B217] Mitchison T. J. (1989). Polewards microtubule flux in the mitotic spindle - evidence from photoactivation of fluorescence. J. Cell Biol. 109, 637–652. 10.1083/jcb.109.2.637 2760109 PMC2115701

[B218] Mitchison T. J. (2012). The proliferation rate paradox in antimitotic chemotherapy. Mol. Biol. Cell 23, 1–6. 10.1091/mbc.E10-04-0335 22210845 PMC3248889

[B219] Mitchison T. J. (2021). So many ways to naturally kill a cancer cell. BMC Biol. 19, 149. 10.1186/s12915-021-01092-3 34325682 PMC8323262

[B220] Mitchison T. Kirschner M. (1984). Dynamic instability of microtubule growth. Nature 312, 237–242. 10.1038/312237a0 6504138

[B221] Mitchison T. J. Salmon E. D. (1992). Poleward kinetochore fiber movement occurs during both metaphase and anaphase-A in newt lung cell mitosis. J. Cell Biol. 119, 569–582. 10.1083/jcb.119.3.569 1400593 PMC2289668

[B222] Mitchison T. J. Maddox P. Groen A. Cameron L. Perlman Z. Ohi R. (2004). Bipolarization and poleward flux correlate during Xenopus extract spindle assembly. Mol. Biol. Cell 15, 5603–5615. 10.1091/mbc.e04-05-0440 15385629 PMC532038

[B223] Mota M. (1956). A new hypothesis of the anaphase movement. In Proceedings of the International Genetics Symposia, 113–116. Cytologia

[B224] Mount Moran (2016). Advanced research computing Center. Laramie: University of Wyoming. 10.15786/M2FY47

[B225] Mukhtar E. Adhami V. M. Mukhtar H. (2014). Targeting microtubules by natural agents for cancer therapy. Mol. Cancer Ther. 13, 275–284. 10.1158/1535-7163.MCT-13-0791 24435445 PMC3946048

[B353] Murphy R. F. Velliste M. Porreca G. (2003). Robust numerical features for description and classification of subcellular location patterns in fluorescence microscope images. Journal of VLSI Signal Processing 35 (3), 311–321.

[B354] Nabi S. U. Khan A. Siddiqui E. M. Rehman M. U. Alshahrani S. Arafah A. (2022). Mechanisms of mitochondrial malfunction in Alzheimer–s disease: new therapeutic hope. Oxid. Med. Cell. Longev. 4759963. 10.1155/2022/4759963 PMC9124149 35607703

[B226] Narumiya S. Ishizaki T. Uehata M. (2000). Use and properties of ROCK-specific inhibitor Y-27632. Methods Enzym. 325, 273–284. 10.1016/s0076-6879(00)25449-9 11036610

[B227] Network C. G. A. R. (2015). The molecular taxonomy of primary prostate cancer. Cell 163, 1011–1025. 10.1016/j.cell.2015.10.025 26544944 PMC4695400

[B228] Ngan V. K. Bellman K. Panda D. Hill B. T. Jordan M. A. Wilson L. (2000). Novel actions of the antitumor drugs vinflunine and vinorelbine on microtubules. Cancer Res. 60, 5045–5051. 11016627

[B229] Nissley D. V. Stephen A. G. Yi M. McCormick F. (2024). Progress in targeting KRAS directly. Methods Mol. Biol. Clift. N.J. 2797, 1–12. 10.1007/978-1-0716-3822-4_1 38570448

[B230] Nussinov R. Jang H. Zhang M. Tsai C. J. Sablina A. A. (2020). The mystery of Rap1 suppression of oncogenic ras. Trends Cancer 6, 369–379. 10.1016/j.trecan.2020.02.002 32249186 PMC7211489

[B231] O'Rourke B. Yang C. P. Sharp D. Horwitz S. B. (2014). Eribulin disrupts EB1-microtubule plus-tip complex formation. Cell Cycle (*Georgetown, Tex.*) 13, 3218–3221. 10.4161/15384101.2014.950143 25485501 PMC4614316

[B232] Oestergren G. (1945). Equilibrium of trivalents and the mechanism of chromosome movements. Hereditas 31, 498. 21021082

[B233] Olumi A. F. Grossfeld G. D. Hayward S. W. Carroll P. R. Tlsty T. D. Cunha G. R. (1999). Carcinoma-associated fibroblasts direct tumor progression of initiated human prostatic epithelium. Cancer Res. 59, 5002–5011. 10.1186/bcr138 10519415 PMC3300837

[B234] Orr G. A. Verdier-Pinard P. McDaid H. Horwitz S. B. (2003). Mechanisms of Taxol resistance related to microtubules. Oncogene 22, 7280–7295. 10.1038/sj.onc.1206934 14576838 PMC4039039

[B235] Pao Y.-S. Liao K.-J. Shiau Y.-C. Chao M.-H. Li M.-C. Lin L.-M. (2025). KIF2C promotes paclitaxel resistance by depolymerizing polyglutamylated microtubules. Dev. Cell. 60, 2097–2113.e8. 10.1016/j.devcel.2025.03.004 40157365

[B236] Pasapera A. M. Schneider I. C. Rericha E. Schlaepfer D. D. Waterman C. M. (2010). Myosin II activity regulates vinculin recruitment to focal adhesions through FAK-mediated paxillin phosphorylation. J. Cell Biol. 188, 877–890. 10.1083/jcb.200906012 20308429 PMC2845065

[B237] Patel V. P. Chu C. T. (2014). Decreased SIRT2 activity leads to altered microtubule dynamics in oxidatively-stressed neuronal cells: implications for Parkinson's disease. Exp. Neurol. 257, 170–181. 10.1016/j.expneurol.2014.04.024 24792244 PMC4141566

[B238] Petry S. Groen A. C. Ishihara K. Mitchison T. J. Vale R. D. (2013). Branching microtubule nucleation in Xenopus egg extracts mediated by augmin and TPX2. Cell 152, 768–777. 10.1016/j.cell.2012.12.044 23415226 PMC3680348

[B239] Piehl M. Cassimeris L. (2003). Organization and dynamics of growing microtubule plus ends during early mitosis. Mol. Biol. Cell 14, 916–925. 10.1091/mbc.e02-09-0607 12631713 PMC151569

[B240] Plebanek M. P. Mutharasan R. K. Volpert O. Matov A. Gatlin J. C. Thaxton C. S. (2015). Nanoparticle targeting and cholesterol flux through scavenger receptor type B-1 inhibits cellular exosome uptake. Sci. Rep. 5, 15724. 10.1038/srep15724 26511855 PMC4625174

[B241] Ponti P. Vallotton P. Matov A. Waterman-Storer C. M. Salmon E. D. Danuser G. (2003). Computational fluorescence speckle microscopy. Biophysical J. 84, 439A. 10.1016/S0006-3495(03)70058-7 PMC1302894 12719263

[B242] Ponti A. Matov A. Adams M. Gupton S. Waterman-Storer C. M. Danuser G. (2005). Periodic patterns of actin turnover in lamellipodia and lamellae of migrating epithelial cells analyzed by quantitative fluorescent speckle microscopy. Biophys. J. 89, 3456–3469. 10.1529/biophysj.104.058701 16100274 PMC1366841

[B243] Prahallad A. Sun C. Huang S. Di Nicolantonio F. Salazar R. Zecchin D. (2012). Unresponsiveness of colon cancer to BRAF(V600E) inhibition through feedback activation of EGFR. Nature 483, 100–103. 10.1038/nature10868 22281684

[B244] Pu Y. Wang Z. Tao S. Yang E. J. Wu S. Ren G. (2025). Microtubule dynamics is a therapeutic vulnerability in VHL-deficient renal cell carcinoma. Int. J. Biol. Sci. 21, 3286–3305. 10.7150/ijbs.109642 40384876 PMC12080383

[B245] Purich D. L. Kristofferson D. (1984). Microtubule assembly: a review of progress, principles, and perspectives. Adv. Protein Chem. 36, 133–212. 10.1016/s0065-3233(08)60297-1 6382962

[B246] Ramaiahgari S. C. Auerbach S. S. Saddler T. O. Rice J. R. Dunlap P. E. Sipes N. S. (2019). The power of resolution: contextualized understanding of biological responses to liver injury chemicals using high-throughput transcriptomics and benchmark concentration modeling. Toxicol. Sci. 169, 553–566. 10.1093/toxsci/kfz065 30850835 PMC6542332

[B247] Ranganathan S. Benetatos C. A. Colarusso P. J. Dexter D. W. Hudes G. R. (1998). Altered beta-tubulin isotype expression in paclitaxel-resistant human prostate carcinoma cells. Br. J. Cancer 77, 562–566. 10.1038/bjc.1998.91 9484812 PMC2149944

[B248] Rao C. V. Kurkjian C. D. Yamada H. Y. (2012). Mitosis-targeting natural products for cancer prevention and therapy. Curr. Drug Targets 13, 1820–1830. 10.2174/138945012804545533 23140292

[B249] Rayleigh L. (1891). On pin-hole photography. Philosophical Mag. J. Sci. 31, 87–100.

[B250] Rieder C. L. Davison E. A. Jensen L. C. Cassimeris L. Salmon E. D. (1986). Oscillatory movements of monooriented chromosomes and their position relative to the spindle pole result from the ejection properties of the aster and half-spindle. J. Cell Biol. 103, 581–591. 10.1083/jcb.103.2.581 3733881 PMC2113830

[B251] Rink J. S. Lin A. Y. McMahon K. M. Calvert A. E. Yang S. Taxter T. (2021). Targeted reduction of cholesterol uptake in cholesterol-addicted lymphoma cells blocks turnover of oxidized lipids to cause ferroptosis. J. Biol. Chem. 296, 100100. 10.1074/jbc.RA120.014888 33208460 PMC7949030

[B252] Ritterhoff T. Das H. Hofhaus G. Schröder R. R. Flotho A. Melchior F. (2016). The RanBP2/RanGAP1*SUMO1/Ubc9 SUMO E3 ligase is a disassembly machine for Crm1-dependent nuclear export complexes. Nat. Commun. 7, 11482. 10.1038/ncomms11482 27160050 PMC4866044

[B253] Robert J. (1999). Multidrug resistance in oncology: diagnostic and therapeutic approaches. Eur. J. Clin. Investigation 29, 536–545. 10.1046/j.1365-2362.1999.00495.x 10354216

[B254] Rogers G. C. Rogers S. L. Sharp D. J. (2005). Spindle microtubules in flux. J. Cell Sci. 118, 1105–1116. 10.1242/jcs.02284 15764594

[B255] Rohrberg J. Van de Mark D. Amouzgar M. Lee J. V. Taileb M. Corella A. (2020). MYC dysregulates mitosis, revealing cancer vulnerabilities. Cell Rep. 30, 3368–3382. 10.1016/j.celrep.2020.02.041 32160543 PMC7085414

[B256] Rothenberg K. E. Chen Y. McDonald J. A. Fernandez-Gonzalez R. (2023). Rap1 coordinates cell-cell adhesion and cytoskeletal reorganization to drive collective cell migration *in vivo* . Curr. Biol. 33, 2587–2601.e5. 10.1016/j.cub.2023.05.009 37244252

[B257] Russo M. Chen M. Mariella E. Peng H. Rehman S. K. Sancho E. (2024). Cancer drug-tolerant persister cells: from biological questions to clinical opportunities. Nat. Rev. Cancer. 24, 694–717. 10.1038/s41568-024-00737-z 39223250 PMC12622869

[B258] Sachs N. Clevers H. (2014). Organoid cultures for the analysis of cancer phenotypes. Curr. Opin. Genet. Dev. 24, 68–73. 10.1016/j.gde.2013.11.012 24657539

[B259] Sachs N. de Ligt J. Kopper O. Gogola E. Bounova G. Weeber F. (2018). A living biobank of breast cancer organoids captures disease heterogeneity. Cell 172, 373–386. 10.1016/j.cell.2017.11.010 29224780

[B260] Sachs N. Papaspyropoulos A. Zomer-van Ommen D. D. Heo I. Böttinger L. Klay D. (2019). Long-term expanding human airway organoids for disease modeling. EMBO J. 38, e100300. 10.15252/embj.2018100300 30643021 PMC6376275

[B261] Sadée C. Testa S. Barba T. Hartmann K. Schuessler M. Thieme A. (2025). Medical digital twins: enabling precision medicine and medical artificial intelligence, Lancet, 7, 100864. 10.1016/j.landig.2025.02.004 PMC12412312 40518342

[B262] Saha T. Dash C. Jayabalan R. Khiste S. Kulkarni A. Kurmi K. (2022). Intercellular nanotubes mediate mitochondrial trafficking between cancer and immune cells. Nat. Nanotechnol. 17, 98–106. 10.1038/s41565-021-01000-4 34795441 PMC10071558

[B263] Salmon E. D. Leslie R. J. Saxton W. M. Karow M. L. McIntosh J. R. (1984). Spindle microtubule dynamics in sea urchin embryos: analysis using a fluorescein-labeled tubulin and measurements of fluorescence redistribution after laser photobleaching. J. Cell Biol. 99, 2165–2174. 10.1083/jcb.99.6.2165 6501418 PMC2113564

[B264] Sang B. Ye L. F. Hu F. Pourfarjam Y. Cuevas-Navarro A. Fan S. (2025). Mechanisms of resistance to active state selective tri-complex RAS inhibitors. bioRxiv 2025, 649345. 10.1101/2025.04.24.649345

[B265] Saporta M. A. Grskovic M. Dimos J. T. (2011). Induced pluripotent stem cells in the study of neurological diseases. Stem Cell Res. and Ther. 2, 37. 10.1186/scrt78 21936964 PMC3308034

[B266] Sato T. Clevers H. (2013). Growing self-organizing mini-guts from a single intestinal stem cell: mechanism and applications, Science, 340. New York, N.Y., 1190–1194. 10.1126/science.1234852 23744940

[B267] Sato T. Vries R. G. Snippert H. J. van de Wetering M. Barker N. Stange D. E. (2009). Single Lgr5 stem cells build crypt-villus structures *in vitro* without a mesenchymal niche. Nature 459, 262–265. 10.1038/nature07935 19329995

[B268] Schaede R. (1929). Kritische Untersuchungen über die Mechanik der Karyokinese. Planta 8, 383–397. 10.1007/bf01916754

[B269] Schulze C. J. Seamon K. J. Zhao Y. Yang Y. C. Cregg J. Kim D. (2023). Chemical remodeling of a cellular chaperone to target the active state of mutant KRAS, Science, 381, 794–799. 10.1126/science.adg9652 37590355 PMC10474815

[B270] Sethi J. K. Jain R. (1987). Finding Trajectories of Feature points in a Monocular image sequence. IEEE Trans. Pattern Anal. Mach. Intell. 9, 56–73. 10.1109/tpami.1987.4767872 21869377

[B271] Shah M. Rahman A. Theoret M. R. Pazdur R. (2021). The drug-dosing conundrum in oncology - when less is more. N. Engl. J. Med. 385, 1445–1447. 10.1056/NEJMp2109826 34623789

[B272] Shannon C. E. (1940). Communication in the presence of noise. Proc. Institute of Radio Engineers 37, 10–21. 10.1109/jrproc.1949.232969

[B273] Simanshu D. K. Nissley D. V. McCormick F. (2017). RAS proteins and their regulators in human disease. Cell 170, 17–33. 10.1016/j.cell.2017.06.009 28666118 PMC5555610

[B274] Simanshu D. K. Xu R. Stice J. P. Czyzyk D. J. Feng S. Denson J.-P. (2025). BBO-10203 inhibits tumor growth without inducing hyperglycemia by blocking RAS-PI3Kα interaction. *Science* eadq2004 389, 409–415. 10.1126/science.adq2004 40504949

[B275] Singhal A. Li B. T. O'Reilly E. M. (2024). Targeting KRAS in cancer. Nat. Med. 30, 969–983. 10.1038/s41591-024-02903-0 38637634 PMC11845254

[B276] Sinkevicius K. Stice J. Riegler E. Feng S. Zhang C. Czyzyk D. J. (2025). Abstract PS7-04: BBO-10203, a first-in-class, orally bioavailable, selective blocker of the PI3Kα:RAS interaction inhibits tumor growth alone and in combination with standard of care therapies in breast cancer models without inducing hyperglycemia. Clin. Cancer Res. 31, PS7-04. 10.1158/1557-3265.sabcs24-ps7-04

[B277] Sironi L. Solon J. Conrad C. Mayer T. U. Brunner D. Ellenberg J. (2011). Automatic quantification of microtubule dynamics enables RNAi-screening of new mitotic spindle regulators. Cytoskelet. Hob. N.J. 68, 266–278. 10.1002/cm.20510 21491614

[B278] Slep K. C. Vale R. D. (2007). Structural basis of microtubule plus end tracking by XMAP215, CLIP-170, and EB1. Mol. Cell 27, 976–991. 10.1016/j.molcel.2007.07.023 17889670 PMC2052927

[B279] Smole Z. Thoma C. R. Applegate K. T. Duda M. Gutbrodt K. L. Danuser G. (2014). Tumor suppressor NF2/Merlin is a microtubule stabilizer. Cancer Res. 74, 353–362. 10.1158/0008-5472.CAN-13-1334 24282279 PMC3929585

[B280] Sobel I. Feldman G. (1968). A 3x3 isotropic gradient operator for image processing. Stanf. Artif. Intell. Proj.

[B281] Solomon B. J. Akerley W. L. Ganju V. Zhang J. Adjei A. Johnson M. L. (2025). Onkoras-101: a phase 1a/1b open-label study evaluating the safety, tolerability, pharmacokinetics, and efficacy of BBO-8520 in subjects with advanced KRASG12C mutant non-small-cell lung cancer. J. Clin. Oncol. 43. 10.1200/jco.2025.43.16_suppl.tps8649

[B282] Spanjaard E. Smal I. Angelopoulos N. Verlaan I. Matov A. Meijering E. (2015). Quantitative imaging of focal adhesion dynamics and their regulation by HGF and Rap1 signaling. Exp. Cell Res. 330, 382–397. 10.1016/j.yexcr.2014.10.012 25447308

[B283] Sparrow C. M. (1916). On spectroscopic resolving power. Astrophysical J. 44, 76. 10.1086/142271

[B284] Staller P. Sulitkova J. Lisztwan J. Moch H. Oakeley E. J. Krek W. (2003). Chemokine receptor CXCR4 downregulated by von Hippel-Lindau tumour suppressor pVHL. Nature 425, 307–311. 10.1038/nature01874 13679920

[B285] Stephen A. G. Esposito D. Bagni R. K. McCormick F. (2014). Dragging ras back in the ring. Cancer Cell 25, 272–281. 10.1016/j.ccr.2014.02.017 24651010

[B286] Stoehr S. J. Smolen J. E. (1990). Human neutrophils contain a protein kinase C-like enzyme that utilizes guanosine triphosphate as a phosphate donor. Cofactor requirements, kinetics, and endogenous acceptor proteins. Blood 75, 479–487. 10.1182/blood.v75.2.479.bloodjournal752479 2295003

[B287] Straight A. F. Cheung A. Limouze J. Chen I. Westwood N. J. Sellers J. R. (2003). Dissecting temporal and spatial control of cytokinesis with a myosin II inhibitor. Science 299, 1743–1747. 10.1126/science.1081412 12637748

[B288] Sumner T. Shephard E. Bogle I. D. (2012). A methodology for global-sensitivity analysis of time-dependent outputs in systems biology modelling, J. R. Soc., 9. 2156–2166. 10.1098/rsif.2011.0891 22491976 PMC3405739

[B289] Swerling P. (1959). First order error propagation in a stagewise smoothing procedure for satellite obvervations. J. Astronautical Sci. 6.

[B290] Tegha-Dunghu J. Bausch E. Neumann B. Wuensche A. Walter T. Ellenberg J. (2014). MAP1S controls microtubule stability throughout the cell cycle in human cells. J. Cell Sci. 127, 5007–5013. 10.1242/jcs.136457 25300793

[B291] Therman E. Timonen S. (1950). Multipolar spindles in human. Cancer Cells. 36, 393–405. 10.1111/j.1601-5223.1950.tb03385.x

[B292] Thoma C. R. Matov A. Gutbrodt K. L. Hoerner K. R. Krek W. Danuser G. (2010). Quantitative image analysis identifies pVHL as a key regulator of microtubule dynamic instability. J. Cell Biol. 190, 991–1003. 10.1083/jcb.201006059 20855504 PMC3101603

[B293] Thomas G. Girre C. Scherrmann J. M. Francheteau P. Steimer J. L. (1989). Zero-order absorption and linear disposition of oral colchicine in healthy volunteers. Eur. J. Clin. Pharmacol. 37, 79–84. 10.1007/BF00609430 2591469

[B294] Thomas G. E. Sreeja J. S. Gireesh K. K. Gupta H. Manna T. K. (2015). +TIP EB1 downregulates paclitaxelinduced proliferation inhibition and apoptosis in breast cancer cells through inhibition of paclitaxel binding on microtubules. Int. J. Oncol. 46, 133–146. 10.3892/ijo.2014.2701 25310526

[B295] Tian S. Simon I. Moreno V. Roepman P. Tabernero J. Snel M. (2013). A combined oncogenic pathway signature of BRAF, KRAS and PI3KCA mutation improves colorectal cancer classification and cetuximab treatment prediction. Gut 62, 540–549. 10.1136/gutjnl-2012-302423 22798500 PMC3596735

[B296] Tiriac H. Belleau P. Engle D. D. Plenker D. Deschênes A. Somerville T. D. D. (2018). Organoid profiling identifies common responders to chemotherapy in pancreatic cancer. Cancer Discov. 8, 1112–1129. 10.1158/2159-8290.CD-18-0349 29853643 PMC6125219

[B297] Tolg C. Hamilton S. R. Morningstar L. Zhang J. Zhang S. Esguerra K. V. (2010). RHAMM promotes interphase microtubule instability and mitotic spindle integrity through MEK1/ERK1/2 activity. J. Biol. Chem. 285, 26461–26474. 10.1074/jbc.M110.121491 20558733 PMC2924079

[B298] Tournebize R. Andersen S. S. L. Verde F. Doree M. Karsenti E. Hyman A. A. (1997). Distinct roles of PP1 and PP2A-like phosphatases in control of microtubule dynamics during mitosis. Embo J. 16, 5537–5549. 10.1093/emboj/16.18.5537 9312013 PMC1170186

[B299] Toyokuni S. Yanatori I. Kong Y. Zheng H. Motooka Y. Jiang L. (2020). Ferroptosis at the crossroads of infection, aging and cancer. Cancer Sci. 111, 2665–2671. 10.1111/cas.14496 32437084 PMC7419040

[B300] Tran A. Matov A. Sung M. Brown L. Hassane D. C. Giannakakou P. (2012). Microtubules regulate tumor microenvironment remodeling, in Understanding cancer: genomes to devices symposium, New York Academy of Medicine.

[B301] Tran A. Kita K. Matov A. Hassane D. C. Brown L. M. Giannakakou P. (2013). Abstract 5162: Microtubule stabilization alters tumor secretome and fibroblast activation. Cancer Res. 73, 5162. 10.1158/1538-7445.am2013-5162

[B302] Tripathi B. K. Hirsh N. H. Qian X. Durkin M. E. Wang D. Papageorge A. G. (2024). The pro-oncogenic noncanonical activity of a RAS GTP:RanGAP1 complex facilitates nuclear protein export. Nat. Cancer 5, 1902–1918. 10.1038/s43018-024-00847-5 39528835 PMC11663792

[B303] TrueBinding I. (2023). Expanded access with TB006 treatment in adults with Alzheimer's Disease and related dementias. ClinicalTrials.gov.

[B304] Tsygankova O. M. Wang H. Meinkoth J. L. (2013). Tumor cell migration and invasion are enhanced by depletion of Rap1 GTPase-activating protein (Rap1GAP). J. Biol. Chem. 288, 24636–24646. 10.1074/jbc.M113.464594 23864657 PMC3750161

[B305] US Food Drug Administration (2013). Paving the way for personalized medicine: fda’s role in a new era of medical product development, in Silver Spring, MD: US Food and Drug Administration, 1–61.

[B306] Vale R. D. Reese T. S. Sheetz M. P. (1985). Identification of a novel force-generating protein, kinesin, involved in microtubule-based motility. Cell 42, 39–50. 10.1016/s0092-8674(85)80099-4 3926325 PMC2851632

[B307] Vallotton P. Ponti A. Waterman-Storer C. M. Salmon E. D. Danuser G. (2003). Recovery, visualization, and analysis of actin and tubulin polymer flow in live cells: a fluorescent speckle microscopy study. Biophys. J. 85, 1289–1306. 10.1016/S0006-3495(03)74564-0 12885672 PMC1303246

[B308] van de Wetering M. Francies H. E. Francis J. M. Bounova G. Iorio F. Pronk A. (2015). Prospective derivation of a living organoid biobank of colorectal cancer patients. Cell 161, 933–945. 10.1016/j.cell.2015.03.053 25957691 PMC6428276

[B309] Vaswani A. Shazeer N. Parmar N. Uszkoreit J. Jones L. Gomez A. N. (2017). Attention is all you need. In Proceedings of the 31st International Conference on Neural Information Processing Systems (Long Beach, California, USA: Curran Associates Inc.), 6000–6010.

[B310] Vecchione L. Gambino V. Raaijmakers J. Schlicker A. Fumagalli A. Russo M. (2016). A vulnerability of a subset of Colon cancers with potential clinical utility. Cell 165, 317–330. 10.1016/j.cell.2016.02.059 27058664

[B311] Vemu A. Szczesna E. Zehr E. A. Spector J. O. Grigorieff N. Deaconescu A. M. (2018). Severing enzymes amplify microtubule arrays through lattice GTP-tubulin incorporation, Science (1979). 361:eaau1504. 10.1126/science.aau1504 30139843 PMC6510489

[B312] Vlachogiannis G. Hedayat S. Vatsiou A. Jamin Y. Fernández-Mateos J. Khan K. (2018). Patient-derived organoids model treatment response of metastatic gastrointestinal cancers, Science (1979), 359:920–926. 10.1126/science.aao2774 29472484 PMC6112415

[B313] Vorobjev I. A. Rodionov V. I. Maly I. V. Borisy G. G. (1999). Contribution of plus and minus end pathways to microtubule turnover. J. Cell Sci. 112, 2277–2289. 10.1242/jcs.112.14.2277 10381384

[B314] Wan N. Zheng J. (2021). MicroRNA-891a-5p is a novel biomarker for non-small cell lung cancer and targets HOXA5 to regulate tumor cell biological function. Oncol. Lett. 22, 507. 10.3892/ol.2021.12768 33986868 PMC8114465

[B315] Wang K. Li M. Hakonarson H. (2010). ANNOVAR: functional annotation of genetic variants from high-throughput sequencing data. Nucleic Acids Res. 38, e164. 10.1093/nar/gkq603 20601685 PMC2938201

[B316] Wang D. Gu J. Wang T. Ding Z. (2014). OncomiRDB: a database for the experimentally verified oncogenic and tumor-suppressive microRNAs. Bioinforma. Oxf. Engl. 30, 2237–2238. 10.1093/bioinformatics/btu155 24651967

[B317] Wang V. E. Doench J. Root D. Bernards R. Settleman J. McCormick F. (2017a). Abstract 3182: cytoskeletal modulation results in increased tumor survival and drug resistance through attenuation of p53 dependent apoptosis. Cancer Res. 77, 3182. 10.1158/1538-7445.am2017-3182

[B318] Wang W. Cantos-Fernandes S. Lv Y. Kuerban H. Ahmad S. Wang C. (2017b). Insight into microtubule disassembly by kinesin-13s from the structure of Kif2C bound to tubulin. Nat. Commun. 8, 70. 10.1038/s41467-017-00091-9 28694425 PMC5503940

[B319] Wang L. Lu Q. Jiang K. Hong R. Wang S. Xu F. (2022a). BRAF V600E mutation in triple-negative breast cancer: a case report and literature review. Oncol. Res. Treat. 45, 54–61. 10.1159/000520453 34818649 PMC8985016

[B320] Wang W. Han D. Cai Q. Shen T. Dong B. Lewis M. T. (2022b). MAPK4 promotes triple negative breast cancer growth and reduces tumor sensitivity to PI3K blockade. Nat. Commun. 13, 245. 10.1038/s41467-021-27921-1 35017531 PMC8752662

[B321] Watanabe T. Noritake J. Kaibuchi K. (2005). Regulation of microtubules in cell migration. Trends Cell Biol. 15, 76–83. 10.1016/j.tcb.2004.12.006 15695094

[B322] Waterman-Storer C. M. Salmon E. D. (1998). How microtubules get fluorescent speckles. Biophys. J. 75, 2059–2069. 10.1016/S0006-3495(98)77648-9 9746548 PMC1299878

[B323] Weierstrass K. (1885). Über die analytische Darstellbarkeit sogenannter willkürlicher Functionen einer reellen Veränderlichen, in *Sitzungsberichte der Königlich Preußischen Akademie der Wissenschaften*. II.

[B324] Wilson H. R. Giese S. C. (1977). Threshold visibility of frequency gradient patterns. Vis. Res. 17, 1177–1190. 10.1016/0042-6989(77)90152-3 595381

[B325] Winograd-Katz S. E. Fässler R. Geiger B. Legate K. R. (2014). The integrin adhesome: from genes and proteins to human disease. Nat. Rev. Mol. Cell Biol. 15, 273–288. 10.1038/nrm3769 24651544

[B326] Witkiewicz A. K. Knudsen E. S. (2014). Retinoblastoma tumor suppressor pathway in breast cancer: prognosis, precision medicine, and therapeutic interventions. Breast Cancer Res. BCR 16, 207. 10.1186/bcr3652 25223380 PMC4076637

[B327] Witkiewicz A. K. Chung S. Brough R. Vail P. Franco J. Lord C. J. (2018). Targeting the vulnerability of RB tumor suppressor loss in triple-negative breast cancer. Cell Rep. 22, 1185–1199. 10.1016/j.celrep.2018.01.022 29386107 PMC5967622

[B328] Wittmann T. Waterman-Storer C. M. (2001). Cell motility: can Rho GTPases and microtubules point the way? J. Cell Sci. 114, 3795–3803. 10.1242/jcs.114.21.3795 11719546

[B329] Wittmann T. Waterman-Storer C. M. (2004). Dynamics in Migrating Cells: a new role for Rho GTPases, in Cell Motility: From Molecules to Organisms. Editors Anne Ridley M. P. Clark P.

[B330] Wittmann T. Waterman-Storer C. M. (2005). Spatial regulation of CLASP affinity for microtubules by Rac1 and GSK3 beta in migrating epithelial cells. J. Cell Biol. 171, 393. 10.1083/jcb.200412114 PMC2171640 15955847

[B331] Wittmann T. Bokoch G. M. Waterman-Storer C. M. (2003). Regulation of leading edge microtubule and actin dynamics downstream of Rac1. J. Cell Biol. 161, 845–851. 10.1083/jcb.200303082 12796474 PMC2172968

[B332] Wittmann T. Bokoch G. M. Waterman-Storer C. M. (2004). Regulation of microtubule destabilizing activity of Op18/Stathmin downstream of Rac1. J. Biol. Chem. 279, 6196–6203. 10.1074/jbc.M307261200 14645234

[B333] Wolff A. W. Peine J. Höfler J. Zurek G. Hemker C. Lingor P. (2024). SAFE-ROCK: a phase I trial of an oral application of the ROCK inhibitor fasudil to assess bioavailability, safety, and tolerability in healthy participants. CNS Drugs 38, 291–302. 10.1007/s40263-024-01070-7 38416402 PMC10980656

[B334] Wood L. (2022). Glycogen Synthase Kinase 3 (GSK3) inhibitor - Pipeline Insight, 2022. Clin. Trials, 60.

[B335] Wu X. Shen Q. T. Oristian D. S. Lu C. P. Zheng Q. Wang H. W. (2011). Skin stem cells orchestrate directional migration by regulating microtubule-ACF7 connections through GSK3β. Cell 144, 341–352. 10.1016/j.cell.2010.12.033 21295697 PMC3050560

[B336] Xie S. Ogden A. Aneja R. Zhou J. (2015). Microtubule-Binding proteins as promising biomarkers of Paclitaxel sensitivity in cancer chemotherapy. Med. Res. Rev. 36, 300–312. 10.1002/med.21378 26332739 PMC4778546

[B337] Yang G. Matov A. Danuser G. (2005). Reliable tracking of large scale dense antiparallel particle motion for fluorescence live cell imaging. In Proc. IEEE Computer Vision and Pattern Recognition (San Diego), 138–146.

[B338] Yang G. Houghtaling B. R. Gaetz J. Liu J. Z. Danuser G. Kapoor T. M. (2007). Architectural dynamics of the meiotic spindle revealed by single-fluorophore imaging. Nat. Cell Biol. 9, 1233–1242. 10.1038/ncb1643 17934454

[B339] Yang G. Cameron L. A. Maddox P. Salmon E. D. Danuser G. (2008). Regional variation of microtubule flux reveals microtubule Organization in the metaphase meiotic spindle. J. Cell Biol. 182, 631–639. 10.1083/jcb.200801105 18710922 PMC2518697

[B340] Yang Z. Su W. Wei X. Qu S. Zhao D. Zhou J. (2023). HIF-1α drives resistance to ferroptosis in solid tumors by promoting lactate production and activating SLC1A1. Cell Rep. 42, 112945. 10.1016/j.celrep.2023.112945 37542723

[B341] Ye A. A. Deretic J. Hoel C. M. Hinman A. W. Cimini D. Welburn J. P. (2015). Aurora A kinase contributes to a pole-based error correction pathway. Curr. Biol. 25, 1842–1851. 10.1016/j.cub.2015.06.021 26166783 PMC4509859

[B342] Yeakley J. M. Shepard P. J. Goyena D. E. VanSteenhouse H. C. McComb J. D. Seligmann B. E. (2017). A trichostatin A expression signature identified by TempO-Seq targeted whole transcriptome profiling. PLoS One 12, e0178302. 10.1371/journal.pone.0178302 28542535 PMC5444820

[B343] Yildiz A. (2025). Mechanism and regulation of kinesin motors. Nat. Rev. Mol. Cell Biol. 26, 86–103. 10.1038/s41580-024-00780-6 39394463

[B344] Yousef Yengej F. A. Jansen J. Rookmaaker M. B. Verhaar M. C. Clevers H. (2020). Kidney organoids and tubuloids. Cells 9, 1326. 10.3390/cells9061326 32466429 PMC7349753

[B345] Zhang D. Rogers G. C. Buster D. W. Sharp D. J. (2007). Three microtubule severing enzymes contribute to the “Pacman-flux” machinery that moves chromosomes. J. Cell Biol. 177, 231–242. 10.1083/jcb.200612011 17452528 PMC2064132

[B346] Zhang W. Lee W. Y. Siegel D. S. Tolias P. Zilberberg J. (2014). Patient-specific 3D microfluidic tissue model for multiple myeloma. Tissue Eng. Part C Methods 20, 663–670. 10.1089/ten.TEC.2013.0490 24294886

[B347] Zhang W. Gu Y. Sun Q. Siegel D. S. Tolias P. Yang Z. (2015). *Ex Vivo* maintenance of primary human multiple myeloma cells through the optimization of the osteoblastic niche. PLoS One 10, e0125995. 10.1371/journal.pone.0125995 25973790 PMC4431864

[B348] Zhang Y. Larraufie M. H. Musavi L. Akkiraju H. Brown L. M. Stockwell B. R. (2018). Design of small molecules that compete with nucleotide binding to an engineered oncogenic KRAS allele. Biochemistry 57, 1380–1389. 10.1021/acs.biochem.7b01113 29313669 PMC5960803

[B349] Zhao X. Lian X. Xie J. Liu G. (2023). Accumulated cholesterol protects tumours from elevated lipid peroxidation in the microenvironment. Redox Biol. 62, 102678. 10.1016/j.redox.2023.102678 36940607 PMC10036943

[B350] Zheng M. W. Zhang C. H. Chen K. Huang M. Li Y. P. Lin W. T. (2016). Preclinical evaluation of a novel orally available SRC/Raf/VEGFR2 inhibitor, SKLB646, in the treatment of triple-negative breast cancer. Mol. Cancer Ther. 15, 366–378. 10.1158/1535-7163.MCT-15-0501 26721945

[B351] Zhu S. Paydar M. Wang F. Li Y. Wang L. Barrette B. (2020). Kinesin Kif2C in regulation of DNA double strand break dynamics and repair. eLife 9, e53402. 10.7554/eLife.53402 31951198 PMC7012618

[B352] Zhu Y. Xie J. Shi J. (2021). Rac1/ROCK-driven membrane dynamics promote natural killer cell cytotoxicity *via* granzyme-induced necroptosis. BMC Biol. 19, 140. 10.1186/s12915-021-01068-3 34325694 PMC8323222

