# Supplementary material for: Modulation of the cytoskeleton for cancer therapy
Source: Front Cell Dev Biol. 2026 Aug 24;13:1681065. doi: 10.3389/fcell.2025.1681065 (PMC13548813; doi:10.3389/fcell.2025.1681065)
Supplement: Supplementary file 1 [file Supplementaryfile1.doc]

**SUPPLEMENTARY MATERIALS**

Figure S1 – Models of overlap of MT bundles.

Figure S2 – Metastatic prostate cancer organoids obtained from micro-metastasis at a retroperitoneal lymph node. Drug-naïve tumor.

Figure S3 – Intraductal prostate cancer organoids obtained from organ-confined disease.

Figure S4 – Primary prostate cancer organoids obtained from radical prostatectomy tissue.

Figure S5 – Metastatic prostate cancer organoids obtained from SU2C soft tissue (liver) distant metastasis.

Figure S6 – Metastatic prostate cancer organoids obtained from metastasis at a retroperitoneal lymph node. Prior patient treatment with androgen depravation therapy, bicalutamide, docetaxel, and carboplatin. Single cells provided from (Gao et al., 2014).

Figure S7 – Lattice light-sheet imaging of prostate cancer organoids.

Figure S8 – Scale space-based speckle detection and orientation-sensitive texture filter.

Video 1 – Real-time detection and tracking of MT ends (Matov, 2024b) in a 30-micron droplet. The angular orientation of the EB1 comets is shown on the histogram to the right in different shades of green. The speed distribution of the EB1 comets is shown on the lower histogram in red with faster comet speeds displayed in brighter red.

Video 2 – Anaphase, EB1 detection. MT ends are labeled with EB1ΔC-2xEGFP, imaged for two minutes during anaphase (with an acquisition rate of an image every two seconds), and computationally tracked (Yang et al., 2005).

Video 3 – Lattice light-sheet imaging of breast cancer EB1 comets in Matrigel. Breast cancer cells were cultured in 3D and plated on a custom rose-chamber in a Matrigel drop. EB1ΔC-2xEGFP labeled MT ends were imaged using lattice light-sheet microscopy.

Video 4 – Prostate organoid derived from radical prostatectomy with labeled MT ends four days after surgery.

Video 5 – Colon cancer organoid with labeled MT ends after 2 nM vinorelbine treatment.

Video 6 – Mitotic spindle (1^st^ example), scale space speckle detection. Speckles were detected (left panel – raw images, right panel – detections) via scale space-based detection (Matov, 2024a; Matov et al., 2011).

Video 7 – Mitotic spindle (1^st^ example, aligned), scale space speckle detection. Speckles were detected (left panel – raw images, right panel – detections) via scale space-based detection (Matov, 2024a; Matov et al., 2011).

Video 8 – Mitotic spindle (2^nd^ example), fluorescent speckles.

Video 9 – Mitotic spindle (2^nd^ example), scale space speckle detection. Speckles were detected via scale space-based detection (Matov, 2024a; Matov et al., 2011).

Video 10 – Mitotic spindle (1^st^ example), speckle streak patterns. Recursive accumulation over the full time-lapse image sequences of fading speckle streak patterns in which speckles with long lifetimes are visible as bright streaks.

Video 11 – Mitotic spindle (2^nd^ example), speckle streak patterns. Recursive accumulation over the full time-lapse image sequences of fading speckle streak patterns in which speckles with long lifetimes are visible as bright streaks.

Video 12 – Mitotic spindle in a prostate cancer organoid derived from a retroperitoneal lymph node with labeled MT ends.

**Figure S1. Models of overlap of MT bundles.** Two models of interpolar MT bundles organization. Individual MTs have their minus ends toward the two spindle poles and their plus ends toward the midzone. Molecular polar proteins link the overlapped MTs and depending on the way the bundles overlap, there will be a different number of Eg5/KIF11 molecular motor proteins connecting the opposing bundles. (A) MTs from overlapping bundles do not interdigitate. (B) MTs from overlapping bundles interdigitate.

**Figure S2. Metastatic prostate cancer organoids obtained from micro-metastasis at a retroperitoneal lymph node.** Organoids obtained from 100 mg tissue from radical prostatectomy. Day 174. Organoid whole genome sequencing showed homozygous mutations in *PTEN* and *BRCA2*. Transmitted light microscopy, 20x magnification. Scale bar equals 100 µm.

**Figure S3. Intraductal prostate cancer organoids obtained from organ-confined disease.** Organoids derived from 1 mg tissue (Gleason score 5+4, punch core biopsy) from radical prostatectomy. Ductal prostate cancer is rare and very aggressive. Phase contrast microscopy, 20x magnification. (A), (D-G), (I-L) have a scale bar (in black) that equals 100 µm; panel H has a scale bar (in black) that equals 50 µm. Organoids often formed ((E), (F), and (H)) at the very edge of the Matrigel drop, which we interpreted as a high metastatic potential.

**Figure S4.** P**rimary prostate cancer organoids obtained from radical prostatectomy tissue.** Day 20. (A) Hematoxylin and eosin stain (H&E) slide of the prostate tumor, the area with the green cross marks the source of organoid tissue. (B) High resolution image of the H&E slide from (A) of primary prostate tumor with Gleason Grade 5+4 and 80% tumor density; this tissue was used to establish the organoids. (C) Immunohistochemistry (IHC): ERG(-), we tested the paraffin block for ERG overexpression, it was negative. (D) Immunohistochemistry (IHC): PTEN(+), we tested the paraffin block for loss of PTEN, it was positive. Scale bars equal 100 µm. (E) Tumor histology of the organoid culture by pathology assessment. Scale bar equals 20 µm. (F) Phase contrast image of live-cell culture of the primary cancer organoid resembles lobular epithelium. Scale bar equals 50 µm.

**Figure S5. Metastatic prostate cancer organoids obtained from SU2C soft tissue (liver) distant metastasis.** Organoids derived from ½ mg tissue (biopsy needle core) from WCDT SU2C. (D-H) follow the same (larger) organoid. (H) and (I) are taken at the same time, but with different magnification. Images except in (I) are imaged at 20x using phase contrast microscopy. (I) shows 4x magnification using transmitted light microscopy. All images have a scale bar (in black) that equals 100 µm.

**Figure S6. Metastatic prostate cancer organoids obtained from metastasis at a retroperitoneal lymph node.** (A) Image of organoids 15 days after seeding of single cells. Transmitted light microscopy, 4x magnification. Scale bar equals 300 µm. (B) Image of an organoid overlaid with a widefield fluorescence image of the MT end marker after EFGP-EB1 lentiviral infection and blasticidin selection. Phase contrast microscopy, 20x magnification. Scale bar equals 100 µm. (C) Large organoid obtained after seeding benign prostate cells. Transmitted light microscopy, 20x magnification. Scale bar equals 200 µm.

**Figure S7. Lattice light-sheet imaging of prostate cancer organoids.** (A) Microscope setup. (B) Software controls. (C)

Custom rose chamber with a Matrigel drop with organoids. (D) De-convolved EB1-2xGFP image of an organoid cell. Scale bar equals 10 µm.

**Figure S8. Scale space-based speckle detection and orientation-sensitive texture filter.** (A) Raw image of mitotic spindle imaged by FSM. Widefield fluorescence microscopy. Scale bar equals 10 µm. (B) Scale space speckle detection. Speckles represent clusters of network-bound fluorophores. (C) Speckle streak patterns. (D) Vectors and color-coding for an orientation-sensitive texture filter indicating the direction of local speckle flow.

Gao, D., I. Vela, A. Sboner, P.J. Iaquinta, W.R. Karthaus, A. Gopalan, C. Dowling, J.N. Wanjala, E.A. Undvall, V.K. Arora, J. Wongvipat, M. Kossai, S. Ramazanoglu, L.P. Barboza, W. Di, Z. Cao, Q.F. Zhang, I. Sirota, L. Ran, T.Y. MacDonald, H. Beltran, J.M. Mosquera, K.A. Touijer, P.T. Scardino, V.P. Laudone, K.R. Curtis, D.E. Rathkopf, M.J. Morris, D.C. Danila, S.F. Slovin, S.B. Solomon, J.A. Eastham, P. Chi, B. Carver, M.A. Rubin, H.I. Scher, H. Clevers, C.L. Sawyers, and Y. Chen. 2014. Organoid cultures derived from patients with advanced prostate cancer. *Cell*. 159:176-187.

Matov, A. 2024a. Analysis of Unstructured High-Density Crowded Scenes for Crowd Monitoring. *arXiv*:2408.11836.

Matov, A. 2024b. Real-Time Image Analysis Software Suitable for Resource-Constrained Computing. *arXiv*:2407.15735.

Matov, A., M.M. Edvall, G. Yang, and G. Danuser. 2011. Optimal-Flow Minimum-Cost Correspondence Assignment in Particle Flow Tracking. *Computer vision and image understanding : CVIU*. 115:531-540.

Yang, G., A. Matov, and G. Danuser. 2005. Reliable tracking of large scale dense antiparallel particle motion for fluorescence live cell imaging. *In* Proc. IEEE Computer Vision and Pattern Recognition, San Diego. 138-146.
